# Supplementary material for: Succession of bacterial communities on carrion is independent of vertebrate scavengers
Source: PeerJ. 2020 Jun 10;8:e9307. doi: 10.7717/peerj.9307 (PMC7293191; doi:10.7717/peerj.9307)
Supplement: Dataset S1 — Interactive Krona plots, and the full count table showing the distribution of each OTU among all samples. [file peerj-08-9307-s001.zip › DS1_COW.fresh_stage_krona.html]

Javascript must be enabled to view this page.

members
magnitude
magnitudeUnassigned

COW.week\_1\_krona

0.999999891280103

0.999898287943103

0.000347085643000001

1.8148279e-05

1.8148279e-05

1.8148279e-05

1.8148279e-05

1.8148279e-05

9.0099867e-05

9.0099867e-05

9.0099867e-05

9.0099867e-05

9.0099867e-05

0.000238837497

0.000238837497

0.000238837497

0.000238837497

0.000238837497

0.0882780137870361

0.0160854538219993

0.0160854538219993

0.0102902491669998

0.000432043750000003

0.000389627116000002

1.2018048e-05

3.0398586e-05

0.008036549809

0.008029623957

3.984948e-06

2.940904e-06

1.838055e-05

1.4138884e-05

4.241666e-06

0.000559182260999998

0.000559182260999998

0.000187230695

0.000186366492

8.64203e-07

0.001056862102

0.000714916300999996

2.1021533e-05

0.000320924268

1.6259708e-05

1.6259708e-05

1.6259708e-05

0.00443133347700003

0.000833293723000001

5.900599e-05

9.95726e-05

0.00042410509

0.000250610043

0.00037247486

0.00037247486

0.000815019420000001

0.000815019420000001

0.000753853720000001

0.000743385090000001

2.29606e-06

8.17257e-06

8.4557904e-05

3.68787e-06

2.544995e-05

5.5420084e-05

0.001077117038

0.001076410094

7.06944e-07

0.000495016811999999

0.000495016811999999

0.00134761146999998

0.00134761146999998

9.190278e-06

0.000535332462000002

0.000803088729999998

0.0130039649429996

0.0129408781129996

1.032083e-05

1.032083e-05

1.032083e-05

0.001571554702

0.000329997241000002

0.000329997241000002

0.000632884911000001

0.000517244622

0.000115640289

0.000588878148

0.000588878148

1.8380512e-05

1.8380512e-05

1.41389e-06

1.41389e-06

0.000115341035

4.5328146e-05

4.5328146e-05

4.6993872e-05

4.6993872e-05

2.3019017e-05

2.3019017e-05

0.000101632302

1.48458e-05

1.48458e-05

6.519755e-06

6.519755e-06

4.24166e-06

4.24166e-06

7.6025087e-05

7.6025087e-05

9.0622539e-05

9.0622539e-05

9.0622539e-05

0.001845826431

0.001429496575

0.000984070071

0.000445426504

9.50624e-06

9.50624e-06

0.000406823616

0.00037893831

2.6156896e-05

1.72841e-06

0.0076482536740001

8.64203e-07

8.64203e-07

0.00512990682500005

2.544999e-05

1.7673608e-05

1.41389e-06

0.001479007141

0.003606362196

1.72841e-06

1.72841e-06

1.413889e-05

1.413889e-05

1.72841e-06

1.72841e-06

0.000299197863000002

0.000299197863000002

0.00220068907300001

0.000211032523

0.001968626289

4.241668e-06

1.6788593e-05

0.000817170664

0.000817170664

2.5937645e-05

0.000791233019

0.00065729429

4.321016e-06

4.321016e-06

0.000145024172

0.000107863434

3.7160738e-05

2.59261e-06

2.59261e-06

0.000186977439

0.000186977439

7.0697495e-05

6.7009625e-05

3.68787e-06

4.74691e-06

4.74691e-06

0.000242934648

0.000242934648

8.2861646e-05

8.2861646e-05

8.64203e-07

8.1997443e-05

3.3703924e-05

3.3703924e-05

6.913624e-06

6.913624e-06

2.67903e-05

2.67903e-05

2.9382906e-05

2.9382906e-05

2.9382906e-05

2.9382906e-05

0.0477710347100059

0.0477710347100059

0.0466968697160059

0.00546132285400021

3.0167774e-05

3.456813e-06

0.0053041171270002

0.000121852734

1.728406e-06

0.00108182249700001

2.296066e-06

3.5220574e-05

0.001033043063

6.31418e-06

4.948614e-06

0.0230598844349965

0.000326168917000001

0.001754707424

0.0209790080939976

0.000458134982000001

2.120832e-06

0.000456014150000001

4.3597495e-05

4.999638e-06

3.8597857e-05

0.001472220841

2.827778e-06

0.00016625086

0.001284633902

1.7360271e-05

1.14803e-06

0.00211333935000003

0.00211333935000003

0.000588714474000001

7.37575e-06

0.000581338724000001

1.3234161e-05

6.913629e-06

6.320532e-06

0.000257691438

5.6731958e-05

0.00020095948

6.362494e-06

6.362494e-06

0.00126105888599999

5.655556e-06

3.605411e-05

0.00120092636599999

1.8422854e-05

0.00498450061400003

0.00498450061400003

0.000889073167000001

5.185219e-06

0.000304891878

0.00057899607

0.000450631510000002

0.00011355218

0.000337079330000001

0.00321423806700003

0.000176769821

0.00303746824600002

0.000369029936000001

0.000326530188000001

4.2499748e-05

0.000152945052

0.000152945052

2.0130036e-05

1.6259726e-05

3.87031e-06

0.000798937426999996

0.000796357226999996

2.5802e-06

0.001033162266

6.2211102e-05

1.413888e-06

6.0797214e-05

0.000744115321999997

6.8775094e-05

0.000135733284

0.000539606943999998

5.7262452e-05

5.7262452e-05

1.3338706e-05

5.16041e-06

8.178296e-06

0.000156234684

0.000156234684

4.1002728e-05

4.1002728e-05

4.1002728e-05

0.0110741096129994

0.000134554136

1.792874e-06

1.792874e-06

1.792874e-06

9.105155e-05

9.89722e-06

9.89722e-06

6.9136272e-05

4.321019e-06

6.4815253e-05

9.897224e-06

9.897224e-06

2.120834e-06

2.120834e-06

4.1709712e-05

3.3226384e-05

3.3226384e-05

8.483328e-06

8.483328e-06

0.000211501811

0.000211501811

6.049423e-06

6.049423e-06

1.728406e-06

1.728406e-06

0.000203723982

2.438132e-06

0.000152725082

8.96437e-07

4.7664331e-05

0.0107280536659994

0.00480251546699992

0.00022864858

1.475148e-05

0.0002138971

8.96437e-07

8.96437e-07

0.00014382704

0.00014382704

1.2901e-06

1.2901e-06

2.950296e-05

2.581509e-05

3.68787e-06

0.00032822084

3.68787e-06

0.00032453297

0.000710105471000001

3.448097e-05

0.000640947385000001

3.4677116e-05

7.006962e-05

7.006962e-05

7.776382e-06

7.776382e-06

0.00328217803700001

0.0002129713

0.00049048706

0.00257782324000001

8.96437e-07

0.0057775878530001

0.00150141054399999

0.000190314165

0.00131109637899999

0.000165384793

0.000165384793

0.000101145278

7.06944e-07

0.000100438334

2.212727e-05

2.212727e-05

0.00398751996800001

0.000419710530000004

0.00356639554800005

1.41389e-06

3.6762532e-05

7.777826e-06

7.777826e-06

2.8277762e-05

2.8277762e-05

7.06944e-07

7.06944e-07

0.000109394944

2.21272e-05

2.21272e-05

8.7267744e-05

8.7267744e-05

1.79287e-06

1.79287e-06

1.79287e-06

0.000343450699000002

0.000343450699000002

0.000343450699000002

0.000343450699000002

0.000343450699000002

0.00021196961

8.96437e-07

8.96437e-07

8.96437e-07

8.96437e-07

8.96437e-07

8.239953e-06

8.239953e-06

8.239953e-06

8.239953e-06

8.239953e-06

0.00020283322

0.00020283322

0.00020283322

0.00020283322

0.00020283322

7.06944e-07

7.06944e-07

7.06944e-07

7.06944e-07

7.06944e-07

7.06944e-07

0.00033190839

3.68787e-06

3.68787e-06

3.68787e-06

3.68787e-06

3.68787e-06

0.00032822052

0.00032822052

0.00032822052

0.00032822052

0.00032822052

0.00461489491299995

0.00420104810799998

0.00417150565799998

0.00417150565799998

3.888195e-05

3.888195e-05

1.6527217e-05

8.64203e-07

1.5663014e-05

2.212722e-05

2.212722e-05

0.000254181243

0.000254181243

7.4637775e-05

7.4637775e-05

0.00366727762900001

7.9309376e-05

0.00358796825300001

9.7872624e-05

9.588472e-05

1.987904e-06

1.935161e-05

1.935161e-05

1.935161e-05

1.935161e-05

1.019084e-05

1.019084e-05

1.019084e-05

1.019084e-05

8.0368106e-05

1.7582988e-05

1.7582988e-05

1.7582988e-05

1.7582988e-05

1.0604162e-05

1.0604162e-05

1.0604162e-05

1.0604162e-05

5.2180956e-05

5.2180956e-05

5.2180956e-05

5.2180956e-05

0.000333478699

1.2725e-05

1.2725e-05

1.2725e-05

1.2725e-05

0.00024187018

0.00024187018

0.00024187018

0.00024187018

1.438219e-06

1.438219e-06

1.438219e-06

1.438219e-06

7.74453e-05

7.74453e-05

7.74453e-05

7.74453e-05

0.000895622865999998

0.000895622865999998

1.4847268e-05

7.777826e-06

7.777826e-06

7.777826e-06

7.069442e-06

7.069442e-06

7.069442e-06

4.431161e-06

4.431161e-06

4.431161e-06

4.431161e-06

0.000322777397

2.12083e-06

2.12083e-06

2.12083e-06

1.13111e-05

1.13111e-05

1.13111e-05

0.000164449391

0.000164449391

0.000164449391

1.728406e-06

1.728406e-06

1.728406e-06

5.3290429e-05

5.3290429e-05

5.3290429e-05

8.9877241e-05

8.9877241e-05

8.9877241e-05

0.000534479573999998

0.000534479573999998

0.000534479573999998

5.9383284e-05

3.534718e-06

0.000321849686000001

2.827774e-06

2.3329142e-05

9.897218e-06

8.7661058e-05

2.5996694e-05

1.9087466e-05

1.9087466e-05

1.9087466e-05

1.8380522e-05

7.06944e-07

0.001188610072

0.001187713635

0.000158097825

0.000157201388

0.000157201388

0.000157201388

8.96437e-07

8.96437e-07

8.96437e-07

0.001016183872

2.120834e-06

2.120834e-06

2.120834e-06

8.96437e-07

8.96437e-07

8.96437e-07

1.792874e-06

8.96437e-07

8.96437e-07

8.96437e-07

8.96437e-07

0.001011373727

8.96437e-07

8.96437e-07

0.00101047729

0.00101047729

1.3431938e-05

1.2724994e-05

1.2724994e-05

1.2724994e-05

7.06944e-07

7.06944e-07

7.06944e-07

8.96437e-07

8.96437e-07

8.96437e-07

8.96437e-07

8.96437e-07

1.6419818e-05

1.6419818e-05

1.6419818e-05

1.6419818e-05

1.6419818e-05

5.185218e-06

1.12346e-05

0.00224363826600007

0.00224363826600007

0.00224363826600007

0.00224363826600007

0.00224363826600007

0.00224363826600007

0.000287561132

0.000287561132

3.583382e-06

3.583382e-06

3.583382e-06

3.583382e-06

0.000103243734

6.4329297e-05

6.4329297e-05

6.4329297e-05

3.8050234e-05

3.8050234e-05

3.8050234e-05

8.64203e-07

8.64203e-07

8.64203e-07

0.000180734016

0.000180734016

4.1462997e-05

4.1462997e-05

5.74016e-07

5.74016e-07

0.000138697003

1.3938631e-05

0.000118395872

6.3625e-06

8.64203e-07

8.64203e-07

8.64203e-07

8.64203e-07

8.64203e-07

8.64203e-07

0.106516614702013

0.000325208634

0.000325208634

0.00018070622

0.00016226687

0.00016226687

1.843935e-05

1.843935e-05

4.6862098e-05

4.6862098e-05

4.6862098e-05

2.7570776e-05

2.7570776e-05

2.7570776e-05

7.006954e-05

7.37574e-06

7.37574e-06

6.26938e-05

6.26938e-05

0.104541983006012

0.001611918095

0.001611918095

0.001474039692

4.8464525e-05

0.001425575167

8.64203e-07

8.64203e-07

0.000136307256

2.96917e-05

0.000106615556

7.06944e-07

7.06944e-07

7.172459e-05

7.172459e-05

7.172459e-05

6.047854e-05

1.2901e-06

9.95595e-06

1.0370443e-05

1.0370443e-05

1.0370443e-05

1.0370443e-05

3.9347337e-05

3.9347337e-05

1.4723702e-05

1.4723702e-05

1.2901e-06

1.2901e-06

2.3333535e-05

4.321019e-06

1.9012516e-05

7.1300674e-05

7.1300674e-05

7.1300674e-05

7.1300674e-05

0.000467812571

0.000128051123

1.1233203e-05

1.1233203e-05

2.506951e-05

4.241666e-06

2.0827844e-05

9.174841e-05

3.97247e-05

5.202371e-05

0.000339761448

5.9006e-05

5.9006e-05

9.846494e-05

2.5802e-06

9.588474e-05

9.5856912e-05

8.96437e-07

7.77783e-06

8.7182645e-05

2.442777e-05

2.442777e-05

6.2005826e-05

4.2743076e-05

1.926275e-05

2.044467e-06

2.044467e-06

1.14803e-06

1.14803e-06

8.96437e-07

8.96437e-07

0.00011478623

0.00011478623

8.453912e-05

8.453912e-05

3.024711e-05

3.024711e-05

0.0650342466650225

0.0595472856570034

0.0513247990890027

3.68787e-06

0.000107849594

7.22259e-06

0.0166388779939992

0.00584095573400001

0.0223232811499972

0.006402924157

0.000304838614000001

4.4094122e-05

0.000255559272

5.18522e-06

0.00791764795399997

0.00791764795399997

3.87031e-06

3.87031e-06

3.87031e-06

0.00014573593

0.00012169982

0.00010326047

1.843935e-05

2.403611e-05

2.403611e-05

0.00530996131100021

0.00530996131100021

0.000340761167

4.948612e-06

0.00495726000000021

6.991532e-06

2.7393457e-05

2.7393457e-05

2.7393457e-05

0.001327154108

0.000629518130000001

3.68787e-06

3.68787e-06

0.00044143671

0.00044143671

0.00018439355

0.00018439355

2.212721e-05

2.212721e-05

2.212721e-05

0.000675508768000001

3.1721894e-05

6.362498e-06

2.5359396e-05

0.00014013915

1.843935e-05

0.0001216998

5.6397007e-05

2.3206107e-05

3.31909e-05

0.000417747747

0.00036141184

3.079307e-05

2.5542837e-05

2.950297e-05

2.950297e-05

0.000670422551000001

0.000670422551000001

0.000135467451

0.000135467451

0.0005349551

0.0005349551

0.000631269386000001

0.000631269386000001

0.00055925503

6.50297e-06

0.00055275206

1.14803e-06

1.14803e-06

3.2839596e-05

1.2901e-06

2.8969286e-05

2.58021e-06

1.14803e-06

1.14803e-06

3.68787e-05

3.68787e-05

8.64203e-06

8.64203e-06

8.64203e-06

8.64203e-06

0.0344809438590049

0.00266625202300008

0.00266625202300008

8.1307055e-05

3.975806e-06

0.00246767384200007

0.00011329532

0.0263434037419962

0.00252225643100006

8.64203e-07

0.00197588723800001

2.827778e-06

0.000542677211999998

0.00019914543

0.00019914543

0.0070464346280002

0.0070464346280002

0.00216541213900003

0.001557054814

0.000576987680999998

3.1369644e-05

1.484583e-05

1.2018054e-05

2.827776e-06

0.0131541919119996

3.76652e-06

4.2345936e-05

4.70178e-05

0.00424966464099999

0.00768290753499936

0.00112848948

1.10636e-05

1.10636e-05

0.000211471653

3.1694065e-05

0.000179777588

0.000917724101

1.413888e-06

0.000167918517

0.000748391696

0.000100858018

2.278091e-06

9.8579927e-05

2.474302e-05

2.474302e-05

2.474302e-05

0.00189151133499999

2.7570868e-05

2.82778e-06

1.413888e-06

2.33292e-05

2.12083e-06

2.12083e-06

1.9430079e-05

4.48219e-06

1.4947889e-05

0.000962759002999996

0.000669286176999996

1.722046e-06

1.69667e-05

3.534722e-06

0.000271249358

0.00020826727

4.948608e-06

0.000203318662

0.000671363284999995

0.000671363284999995

0.001188203058

0.001188203058

0.001188203058

0.000453864085000001

0.000453157141000001

0.000131562861

0.00032159428

7.06944e-07

7.06944e-07

3.87031e-06

3.87031e-06

3.87031e-06

4.6889705e-05

8.64203e-07

8.64203e-07

4.6025502e-05

4.6025502e-05

0.000973229410999999

7.2445526e-05

7.2445526e-05

0.000900783884999999

3.68787e-06

0.000171715748

0.000725380267

7.1711957e-05

4.1804147e-05

4.1804147e-05

6.30566e-06

6.30566e-06

2.360215e-05

2.360215e-05

0.000292199642000001

0.000292199642000001

2.120834e-06

5.330013e-05

0.000236778678

1.4797512e-05

1.4797512e-05

5.166146e-06

9.05735e-06

5.74016e-07

0.000413533843000002

0.000413533843000002

2.827778e-06

0.000386508330000002

2.4197735e-05

9.6734216e-05

8.483326e-06

8.483326e-06

4.8933454e-05

4.8933454e-05

3.9317436e-05

6.362492e-06

3.2954944e-05

6.638171e-05

5.53181e-05

5.53181e-05

5.53181e-05

5.53181e-05

1.106361e-05

1.106361e-05

1.106361e-05

1.106361e-05

0.000530790238000001

0.00010498246

0.00010498246

0.00010498246

3.860076e-05

6.63817e-05

0.000425807778000001

0.000425807778000001

0.000425807778000001

0.000356782189

6.9025589e-05

0.000144789034

0.000144789034

0.000144789034

0.000144789034

0.000144789034

2.2154794e-05

2.2154794e-05

2.2154794e-05

2.0740906e-05

2.0740906e-05

1.413888e-06

1.413888e-06

0.000855804326000001

0.000557765377000001

0.000557765377000001

0.000557765377000001

0.000557765377000001

0.000291661768

0.00014013887

0.00014013887

0.00014013887

8.64203e-07

8.64203e-07

8.64203e-07

8.96437e-07

8.96437e-07

8.96437e-07

1.161093e-05

1.161093e-05

1.161093e-05

4.7702304e-05

4.7702304e-05

4.7702304e-05

2.21272e-05

2.21272e-05

2.21272e-05

6.8321824e-05

6.8321824e-05

5.2433382e-05

1.5888442e-05

6.377181e-06

3.68787e-06

3.68787e-06

3.68787e-06

2.689311e-06

2.689311e-06

2.689311e-06

2.950296e-05

2.950296e-05

2.950296e-05

2.950296e-05

2.950296e-05

7.140133e-05

7.140133e-05

7.140133e-05

7.140133e-05

1.4845822e-05

1.4845822e-05

5.6555508e-05

3.534722e-06

4.241664e-06

4.8779122e-05

0.48337314485046

0.000113677023

0.000113677023

0.000113677023

8.4294104e-05

2.7031628e-05

5.7262476e-05

2.9382919e-05

2.592609e-06

2.679031e-05

0.0250201480659986

0.001092524887

8.96437e-07

8.96437e-07

8.96437e-07

2.985037e-06

2.985037e-06

2.985037e-06

0.000180939418

0.000133337416

0.000133337416

4.7602002e-05

2.43295e-05

2.0692292e-05

2.58021e-06

0.000907703995

5.900592e-05

5.900592e-05

8.64203e-07

8.64203e-07

0.000189697386

0.000186712349

8.64203e-07

2.120834e-06

0.000656272366000001

1.106361e-05

0.000609072806000001

1.769658e-05

1.843937e-05

1.86412e-06

1.86412e-06

4.7232278e-05

3.8174934e-05

3.8174934e-05

3.8174934e-05

9.057344e-06

9.057344e-06

9.057344e-06

0.00212235604200002

0.000228793506

0.000207585194

4.241662e-06

0.000149743469

6.45052e-06

9.506243e-06

3.76433e-05

2.1208312e-05

2.1208312e-05

0.00044089175

2.827778e-06

2.827778e-06

9.496572e-06

9.496572e-06

0.000381579076

6.4422206e-05

0.00031715687

4.6988324e-05

8.96437e-07

8.96437e-07

1.14803e-06

4.404742e-05

0.00058655469

0.00058655469

0.00058655469

7.37575e-06

7.37575e-06

7.37575e-06

0.000858740346

0.000810224

0.000810224

4.8516346e-05

4.8516346e-05

5.65555e-06

5.65555e-06

5.65555e-06

5.65555e-06

0.00454179603000013

2.43813e-06

1.2901e-06

1.2901e-06

1.14803e-06

1.14803e-06

0.000656966361999998

0.000162053358

4.108736e-06

0.000157944622

1.1220444e-05

1.1220444e-05

0.000222536186

0.000222536186

0.000261156374

5.5505131e-05

0.000205651243

0.000497727562

0.000497727562

0.000497727562

4.2219473e-05

1.106361e-05

1.106361e-05

3.1155863e-05

3.1155863e-05

0.00138615553299999

1.2018054e-05

1.2018054e-05

2.58021e-06

2.58021e-06

6.362494e-06

2.120832e-06

4.241662e-06

0.00125971315699999

0.00125971315699999

3.1812488e-05

3.1812488e-05

3.303772e-05

3.303772e-05

3.9924466e-05

3.410932e-06

3.6513534e-05

7.06944e-07

7.06944e-07

0.00149959842499999

0.00118507767399999

0.00118507767399999

0.000180071901

1.14803e-06

0.000137399267

4.1524604e-05

0.00013444885

6.830769e-05

4.948614e-06

1.3827256e-05

4.736529e-05

1.48458e-05

1.48458e-05

1.48458e-05

0.000289688286000001

1.57014e-05

1.57014e-05

0.000187787794

7.06944e-07

0.00018708085

8.6199092e-05

8.6199092e-05

9.5941922e-05

4.056661e-05

2.950299e-05

1.106362e-05

3.68787e-05

3.68787e-05

1.8496612e-05

1.8496612e-05

5.6214537e-05

5.6214537e-05

5.6214537e-05

0.00734718214599999

0.00734718214599999

8.64203e-07

8.64203e-07

0.00734542150599999

0.000550534616

5.74016e-07

0.00679172026399999

2.59261e-06

8.96437e-07

8.96437e-07

0.000259609991

2.120832e-06

2.120832e-06

2.120832e-06

0.00021758435

0.00021758435

0.00021758435

9.506239e-06

9.506239e-06

5.185219e-06

4.32102e-06

3.039857e-05

3.039857e-05

3.039857e-05

0.00938888085199944

0.00938888085199944

0.000112657585

7.9893207e-05

3.2764378e-05

0.00213147130800004

0.00213147130800004

1.9087488e-05

1.413888e-06

1.76736e-05

0.00491444188799994

5.16042e-06

0.00483824674599994

2.82778e-06

3.5687554e-05

3.2519388e-05

0.000581703804

5.6578808e-05

0.00021838122

0.000306743776000001

6.229568e-06

1.413888e-06

7.06944e-07

4.108736e-06

0.00161812879099999

0.00133707568499999

3.7753318e-05

0.000243299788

5.16042e-06

5.16042e-06

0.00021491029

0.00021491029

0.00021491029

0.00021491029

0.439953524959428

5.6969664e-05

5.6969664e-05

4.321013e-06

8.64203e-07

3.45681e-06

5.2648651e-05

5.2648651e-05

0.00840325514000014

0.00835399549300011

0.00200798590100001

0.00023715423

0.001770831671

0.00303325214599999

0.000182657096

0.00285059504999999

0.00330124016100002

0.00037424929

0.00292699087100001

1.1517285e-05

1.1517285e-05

4.9259647e-05

1.4691503e-05

1.4691503e-05

3.4568144e-05

2.6790309e-05

7.777835e-06

4.241664e-06

4.241664e-06

4.241664e-06

4.241664e-06

0.000221357964

0.000221357964

0.000221357964

6.6803244e-05

0.00015340669

1.14803e-06

0.0778653687739879

0.0693166085649836

0.000109304647

0.000109304647

0.069207303917984

0.001511248886

1.72205e-06

1.7421892e-05

0.0676219021889924

7.74062e-06

4.7268281e-05

3.2376627e-05

3.2376627e-05

3.2376627e-05

0.00177175671599998

0.00177175671599998

0.00103017211599999

0.000548589112

0.000188046878

2.12083e-06

2.82778e-06

0.00674462686600023

0.00538516331000022

2.592609e-06

0.000394003488000001

3.913702e-06

0.0049846535110002

0.00135946355599999

0.000579115123999998

4.241664e-06

0.000275792468000001

0.000499607356

7.06944e-07

0.00349980214700009

1.47515e-05

1.47515e-05

1.47515e-05

8.96437e-07

8.96437e-07

8.96437e-07

0.00348415421000009

7.06944e-07

7.06944e-07

8.48333e-06

8.48333e-06

0.000476828751000002

2.827776e-06

8.7203163e-05

0.000386797812000001

1.792874e-06

1.792874e-06

9.6144402e-05

2.827776e-06

9.3316626e-05

2.4972983e-05

2.2662656e-05

2.310327e-06

0.00018596611

0.00017292641

1.016962e-05

2.87008e-06

1.210607e-05

1.210607e-05

0.00236181915300005

0.00236181915300005

8.2332376e-05

3.1470462e-05

5.0861914e-05

0.000233001217

0.000199300181

3.3701036e-05

0.00113308769599999

0.00113308769599999

0.00113308769599999

0.00113308769599999

0.001881045728

0.001881045728

0.001435477139

0.001435477139

0.000148585184

3.0440896e-05

2.1208344e-05

9.6935944e-05

4.592126e-06

4.592126e-06

2.1935496e-05

7.77639e-06

1.4159106e-05

8.1009616e-05

5.74016e-07

8.04356e-05

6.377184e-06

6.377184e-06

5.0322935e-05

3.3910035e-05

1.64129e-05

0.000132746048

7.865269e-05

5.4093358e-05

0.0108247892509998

7.855744e-06

7.855744e-06

7.855744e-06

0.00927102326599985

9.03073e-06

9.03073e-06

7.5643092e-05

4.6658364e-05

4.94861e-06

2.4036118e-05

6.2535619e-05

6.2535619e-05

0.00912381382499987

1.0604164e-05

2.364141e-05

0.00908956825099987

6.049427e-05

6.049427e-05

6.049427e-05

0.000118059578

4.029576e-05

2.6863872e-05

1.3431888e-05

7.7763818e-05

7.7763818e-05

0.001364181865

0.000518567935999999

0.000518567935999999

9.7675968e-05

9.626208e-05

1.413888e-06

0.000357295701000001

0.000349402584000001

7.186173e-06

7.06944e-07

5.2889803e-05

5.2889803e-05

0.000337752457

0.000284447294

5.33051630000001e-05

3.174528e-06

3.174528e-06

3.174528e-06

0.32939529576132

0.0264501343769959

7.06944e-07

7.06944e-07

0.0264494274329959

1.67714e-05

0.001280656696

0.0152183195090002

7.069438e-06

0.000480721614

1.5552774e-05

0.000625174078999997

3.3300816e-05

0.00660063066700002

0.001902332114

2.5105769e-05

7.06944e-07

0.000243085613

0.302945161384305

4.163764e-06

4.163764e-06

0.280262536946251

2.1497952e-05

0.0184903861549988

0.0163693228809998

0.00437383407199999

2.3227542e-05

2.7734266e-05

0.235466136702056

9.178522e-06

0.005465249493

5.74016e-07

1.5395345e-05

5.2051126e-05

7.796606e-06

4.425452e-05

5.6367784e-05

5.6367784e-05

8.2282699e-05

1.7739229e-05

6.454347e-05

0.0224877590649972

0.016308376857

5.74016e-07

5.5373319e-05

1.8794732e-05

1.84394e-05

0.00608620074099951

2.58021e-06

2.58021e-06

2.58021e-06

2.58021e-06

9.506239e-06

6.913626e-06

6.913626e-06

6.913626e-06

2.592613e-06

2.592613e-06

2.592613e-06

8.96437e-07

8.96437e-07

8.96437e-07

8.96437e-07

0.00029129859

0.00029129859

0.00022760391

1.950891e-05

0.000208095

6.369468e-05

6.369468e-05

0.00589177203600019

0.00550925774100017

0.000107813646

2.827778e-06

0.000104985868

0.00154740393699999

3.3383639e-05

0.00151402029799999

3.53472e-06

3.53472e-06

0.00146650682299999

0.000999143451999998

0.000159401965

0.000307961406

2.592613e-06

8.64203e-07

1.72841e-06

0.000149101864

0.000148205427

8.96437e-07

0.00223230413800003

0.00223230413800003

0.000297100046000002

0.000297100046000002

0.000297100046000002

2.6492578e-05

2.6492578e-05

2.6492578e-05

5.8921671e-05

2.278091e-06

2.278091e-06

5.664358e-05

5.664358e-05

0.00036768397

3.073834e-06

2.36689e-06

5.74016e-07

1.792874e-06

7.06944e-07

7.06944e-07

0.000364610136

0.000364610136

0.000354287605

3.476637e-06

6.845894e-06

9.9413268e-05

9.9413268e-05

9.9413268e-05

7.06944e-07

9.8706324e-05

5.16042e-06

5.16042e-06

5.16042e-06

5.16042e-06

0.000381251959000001

0.000381251959000001

0.000381251959000001

0.000381251959000001

0.000381251959000001

0.0162717897059995

0.000502242923

0.000502242923

3.68787e-06

3.68787e-06

0.000498555053

5.378622e-06

0.000493176431

6.631342e-06

6.631342e-06

6.631342e-06

6.631342e-06

0.0154717216029997

0.00323488510600012

0.000381272620000002

0.000194816542

0.00012495197

1.34319e-05

4.8072208e-05

0.000403532646000002

6.362496e-06

0.000225515666

0.000171654484

1.14803e-06

1.14803e-06

9.496574e-06

9.496574e-06

0.000105618962

9.882646e-05

6.792502e-06

4.0295774e-05

4.0295774e-05

3.444096e-06

2.87008e-06

5.74016e-07

0.001119354968

0.00013346749

0.000979524977999997

6.3625e-06

0.001136479936

0.001136479936

2.68639e-05

2.68639e-05

7.3776e-06

7.3776e-06

0.000255767241

0.000255767241

0.000255767241

0.00700836760499998

4.552075e-05

4.552075e-05

0.00696284685499998

7.06944e-07

0.00696213991099998

0.00302393391800002

0.000698011650000002

0.000698011650000002

0.001386621508

0.001379245758

7.37575e-06

0.00052994648

0.00052994648

0.00040935428

7.37574e-06

0.00040197854

0.001948767733

8.96437e-07

8.96437e-07

5.7866772e-05

5.74016e-07

4.01811e-06

5.3274646e-05

0.000269172596

0.000269172596

0.000881066813000002

0.000881066813000002

0.000732389365

8.96437e-07

0.000731492928

7.37575e-06

7.37575e-06

0.000228135294

0.000228135294

2.689311e-06

2.689311e-06

0.000225445983

0.000225445983

6.3058544e-05

6.3058544e-05

6.3625e-06

6.3625e-06

1.413888e-06

1.413888e-06

5.655554e-06

5.655554e-06

4.3123632e-05

4.3123632e-05

6.50297e-06

6.50297e-06

5.74016e-07

5.74016e-07

5.74016e-07

5.74016e-07

5.74016e-07

0.00163217912099999

0.000169055429

4.5724943e-05

4.5724943e-05

4.5724943e-05

1.843934e-05

1.843934e-05

1.843934e-05

3.68787e-06

3.68787e-06

3.68787e-06

8.6400911e-05

7.74062e-06

7.74062e-06

7.8660291e-05

8.96437e-07

7.7763854e-05

6.45052e-06

6.45052e-06

6.45052e-06

7.777829e-06

7.777829e-06

7.777829e-06

5.74016e-07

5.74016e-07

5.74016e-07

0.000252787785

1.3075845e-05

1.3075845e-05

8.239943e-06

3.68787e-06

1.148032e-06

0.00023971194

0.00023971194

0.00023971194

0.000628306246999998

0.000628306246999998

5.0102269e-05

5.0102269e-05

0.000531957159999999

0.000531957159999999

7.74062e-06

7.74062e-06

3.8506198e-05

3.8506198e-05

3.6328768e-05

3.6328768e-05

3.6328768e-05

3.6328768e-05

7.1008966e-05

7.1008966e-05

7.1008966e-05

7.1008966e-05

4.5244456e-05

4.5244456e-05

4.5244456e-05

4.5244456e-05

0.000429447470000002

0.000289814402000001

2.120832e-06

2.120832e-06

6.5038828e-05

6.5038828e-05

0.000222654742

0.000222654742

0.000139633068

7.06944e-07

7.06944e-07

0.000138926124

5.8676314e-05

8.024981e-05

0.000784201174000001

0.000784201174000001

0.000784201174000001

7.6129844e-05

7.6129844e-05

3.5950628e-05

4.0179216e-05

0.000708071330000001

4.794232e-05

4.794232e-05

0.000660129010000001

0.000660129010000001

0.000933475336000001

0.00046467192

0.00046467192

0.00046467192

0.00046467192

0.00046467192

0.000178439214

2.58021e-06

2.58021e-06

2.58021e-06

2.58021e-06

0.000175859004

0.000175859004

2.0836932e-05

7.06944e-07

2.0129988e-05

2.8015263e-05

2.8015263e-05

0.000103389062

0.000103389062

1.2018054e-05

1.2018054e-05

1.1025677e-05

1.1025677e-05

5.74016e-07

5.74016e-07

0.000290364202

3.9588822e-05

3.9588822e-05

3.9588822e-05

3.9588822e-05

0.00025077538

3.68787e-06

3.68787e-06

3.68787e-06

3.68787e-06

3.68787e-06

3.68787e-06

0.00024339964

0.00024339964

0.00024339964

0.000348098249

5.74016e-07

5.74016e-07

5.74016e-07

5.74016e-07

5.74016e-07

0.00033928429

0.00033928429

0.00033928429

0.00033928429

0.00033928429

8.64203e-07

8.64203e-07

8.64203e-07

8.64203e-07

8.64203e-07

7.37574e-06

7.37574e-06

7.37574e-06

7.37574e-06

7.37574e-06

1.1311102e-05

1.1311102e-05

1.1311102e-05

1.1311102e-05

1.1311102e-05

1.1311102e-05

0.305031646386184

0.0317689262979971

1.950891e-05

1.950891e-05

1.950891e-05

1.950891e-05

3.553514e-06

3.553514e-06

3.553514e-06

3.553514e-06

0.0317458638739971

0.0064733474560001

0.000714827408999999

4.48534e-05

9.506233e-06

3.6296542e-05

0.000410982224

0.00021318901

0.000292395053

8.5315976e-05

0.00016866172

3.8417357e-05

0.000364067524000002

0.000148138841

1.83805e-05

0.000174219065

2.3329118e-05

0.000458271485

0.000458271485

0.000197666084

0.000197666084

1.728406e-06

1.728406e-06

6.0494193e-05

1.9876641e-05

4.0617552e-05

8.48333e-06

8.48333e-06

1.1234603e-05

1.1234603e-05

1.5555706e-05

1.5555706e-05

0.00306453559300005

7.37575e-06

0.001697458089

0.001339908536

1.9793218e-05

0.00019405466

4.32102e-06

0.00015430134

3.54323e-05

0.000289044968

3.62965e-05

0.000187933233

1.728406e-06

6.3086829e-05

1.7284109e-05

1.7284109e-05

0.000648091491000001

0.000538758474

0.000107540143

1.792874e-06

5.2716403e-05

8.64203e-07

5.18522e-05

1.4138834e-05

1.4138834e-05

6.049426e-06

1.728406e-06

4.32102e-06

6.2708179e-05

7.77783e-06

5.4930349e-05

0.000289847282000002

0.000289847282000002

1.6966652e-05

0.000272880630000001

0.001005218616

0.000398466977

8.483328e-06

6.441847e-06

0.000383541802

5.1791673e-05

5.1791673e-05

0.000137946399

4.9807799e-05

8.81386e-05

0.000200495207

1.3827246e-05

0.000101111745

8.5556216e-05

1.1234656e-05

3.456816e-06

7.77784e-06

0.000205283704

0.000171219114

3.406459e-05

0.012475284947

1.3827253e-05

4.321013e-06

9.50624e-06

0.004933289692

8.64203e-07

7.20631e-05

0.004704500899

0.00015586149

3.8603752e-05

8.23138e-06

3.0372372e-05

3.6761104e-05

7.06944e-07

3.605416e-05

0.00307292309800006

0.00307292309800006

2.5607289e-05

7.933645e-06

1.69667e-05

7.06944e-07

0.00435427275900002

0.000195684441

0.00415858831800001

2.9382925e-05

2.9382925e-05

2.9382925e-05

0.001751075995

1.728406e-06

8.64203e-07

8.64203e-07

0.001749347589

1.123464e-05

2.96917e-05

0.000226561448

8.4046734e-05

7.006956e-05

0.001327743507

0.00410939755200005

0.00010130092

1.72841e-06

3.68787e-06

9.588464e-05

7.77639e-06

7.77639e-06

0.00016759604

1.2098853e-05

1.20988e-05

0.000143398387

0.002298817133

0.00025361569

0.00204520144300001

1.079634e-05

1.72841e-06

9.06793e-06

8.64203e-07

8.64203e-07

0.000485866365

3.68787e-06

0.000482178495

8.64203e-06

8.64203e-06

3.6761002e-05

7.069438e-06

2.9691564e-05

4.321013e-06

4.321013e-06

0.000615601417999999

0.000615601417999999

1.1234649e-05

1.1234649e-05

5.1533348e-05

6.049426e-06

4.5483922e-05

1.0370443e-05

8.642033e-06

1.72841e-06

3.87031e-06

3.87031e-06

4.5796397e-05

4.5796397e-05

5.5147442e-05

1.1311108e-05

3.5508814e-05

1.41389e-06

6.91363e-06

1.555562e-05

5.18522e-06

1.03704e-05

0.000108805538

0.000104253465

4.552073e-06

1.5555703e-05

1.5555703e-05

1.3431944e-05

1.3431944e-05

3.9753304e-05

3.9753304e-05

0.00013626427

0.00013626427

0.00013626427

8.6883068e-05

2.0501368e-05

2.0501368e-05

6.63817e-05

6.63817e-05

1.3827303e-05

1.3827303e-05

1.3827303e-05

0.00202573725300001

2.29606e-06

2.29606e-06

0.001146606091

9.1605585e-05

1.1714068e-05

9.6364793e-05

0.000943779350999999

2.278091e-06

8.64203e-07

0.000741820883999998

8.7221394e-05

0.000654599489999999

5.145303e-05

1.413888e-06

5.0039142e-05

1.7996628e-05

1.7996628e-05

2.3460961e-05

6.991534e-06

1.6469427e-05

1.3431942e-05

9.190274e-06

4.241668e-06

2.8671657e-05

2.8671657e-05

8.642043e-06

8.642043e-06

8.642043e-06

0.00334095516400005

3.8174944e-05

2.969162e-05

8.483324e-06

0.00226116294400004

0.001843060229

0.000371382644000001

3.3995077e-05

1.2724994e-05

1.2901e-06

1.2901e-06

1.6461526e-05

9.89722e-06

6.564306e-06

2.4317044e-05

1.1311144e-05

1.30059e-05

9.1902706e-05

9.1902706e-05

0.000231118526

1.148032e-06

3.68787e-06

1.8380576e-05

0.000207902048

0.000676527373999999

0.000676527373999999

0.270753691256086

0.0745962779910027

1.4926624e-05

1.4926624e-05

7.148794e-06

7.77783e-06

0.00017894298

0.00017894298

7.06944e-07

0.000178236036

0.045317684473005

3.1837838e-05

3.1837838e-05

0.000220201136

0.000220201136

1.722046e-06

5.74016e-07

1.14803e-06

1.14803e-06

1.14803e-06

0.011332130577

0.000182569986

0.0059346401669999

0.00145106732200002

0.003763853102

0.00402852352500005

0.00402852352500005

0.012244415053

0.000366753766000001

0.0023503227390001

0.00952733854799998

0.002415473452

0.000264468879999999

9.1049763e-05

0.002059954809

0.000147562416

6.1409004e-05

8.6153412e-05

0.0114739148070001

0.001771124919

0.001253990219

0.008448799669

0.0028569593250001

0.00217961373300006

0.000675617185999998

1.728406e-06

0.000563796267999997

0.000563796267999997

0.00589540815099977

0.000393372710000002

0.000225068236

7.06944e-07

0.00016759753

6.18040120000001e-05

6.18040120000001e-05

0.00544023142899977

0.00543420712499977

6.024304e-06

0.000486486977999998

0.000486486977999998

0.000486486977999998

0.000600822190000001

0.000600822190000001

0.000600822190000001

0.0221020065949977

0.001251445688

0.001251445688

0.000458954044

7.06944e-07

0.0004582471

0.00221150796500001

0.00221150796500001

0.0101189641319994

0.00238180653300007

2.4342742e-05

7.06944e-07

0.007712107913

4.241664e-06

4.241664e-06

0.00805689310199967

0.00622208479499999

0.001825003655

7.06944e-07

9.097708e-06

0.195737065117982

0.0643655984770146

0.0253512964729984

1.2901e-06

0.00439038754200014

1.571147e-06

0.007222419258

0.013735628426

6.50297e-06

6.50297e-06

0.00763485630000021

0.00763485630000021

0.00232087296800005

5.0111558e-05

0.00227076141000005

0.00208968239900003

1.41389e-06

1.0332286e-05

1.41389e-06

1.72205e-06

0.00207480028300003

0.0269623873669982

0.0137896040479995

0.013172783319

0.045942704721008

0.045942704721008

0.045942704721008

0.000113330188

7.06944e-07

7.06944e-07

5.7572214e-05

4.97797e-06

4.1835738e-05

1.0758506e-05

4.1619054e-05

1.767362e-05

2.3945434e-05

1.3431976e-05

1.3431976e-05

0.00282156745600007

1.1234639e-05

1.1234639e-05

0.000568746112

1.6812344e-05

0.000551933768

0.000585583475999996

0.000585583475999996

0.001381168096

0.000132955294

0.000499574222

0.000748638580000001

3.6207288e-05

3.6207288e-05

0.000183809487

5.74016e-07

5.65555e-06

1.41911e-05

0.000163388821

4.3992156e-05

8.64203e-07

4.3127953e-05

1.722046e-06

1.722046e-06

9.104156e-06

7.37575e-06

1.728406e-06

0.0824152004519861

0.0519976403710009

0.0519342607540009

2.592613e-06

2.807405e-05

7.06944e-07

3.200601e-05

2.59261e-06

2.59261e-06

5.348153e-05

1.0151324e-05

4.3330206e-05

0.000883368138

2.7654503e-05

3.7975856e-05

0.000817737779

0.001515797413

0.001201494102

0.000314303311

0.027602067722992

0.027602067722992

0.000283731675

0.000100282606

0.000134316299

4.913277e-05

4.6046629e-05

4.6046629e-05

8.0305e-06

8.0305e-06

1.92836e-05

1.92836e-05

8.64203e-07

8.64203e-07

2.29606e-06

2.29606e-06

6.1168058e-05

6.1168058e-05

1.4719414e-05

4.6448644e-05

1.7495766e-05

1.7495766e-05

1.5640792e-05

1.854974e-06

0.000420348146999999

0.000420348146999999

0.000420348146999999

0.000420348146999999

0.000325175797

0.000325175797

0.000303570761

0.000303570761

4.4459237e-05

0.000259111524

2.1605036e-05

2.59261e-06

2.59261e-06

1.3827206e-05

1.3827206e-05

5.18522e-06

5.18522e-06

2.3735606e-05

2.3735606e-05

2.3735606e-05

2.3735606e-05

2.3735606e-05

0.00216011742900005

0.00216011742900005

0.00216011742900005

0.001651422035

2.827778e-06

0.000374239509000001

0.001274354748

1.9876706e-05

1.9876706e-05

1.2098853e-05

1.2098853e-05

0.000403059492

7.9884736e-05

0.000254438088

1.728406e-06

6.7008262e-05

1.413888e-06

1.413888e-06

1.1752192e-05

1.1752192e-05

6.0494263e-05

1.0370441e-05

5.0123822e-05

0.001286939407

2.581507e-05

2.581507e-05

2.581507e-05

2.581507e-05

2.581507e-05

5.74016e-07

5.74016e-07

5.74016e-07

5.74016e-07

5.74016e-07

8.96437e-07

8.96437e-07

8.96437e-07

8.96437e-07

8.96437e-07

0.000236920157

0.000236920157

0.000236920157

0.000236920157

0.000236920157

8.96437e-07

8.96437e-07

8.96437e-07

8.96437e-07

8.96437e-07

0.000281174887

0.000281174887

0.000281174887

0.00027659058

0.00027659058

4.584307e-06

4.584307e-06

6.3590247e-05

6.3590247e-05

6.3590247e-05

6.3590247e-05

6.3590247e-05

2.4623612e-05

2.4623612e-05

2.4623612e-05

2.4623612e-05

2.4623612e-05

7.346957e-06

3.87031e-06

3.87031e-06

3.87031e-06

3.87031e-06

3.476647e-06

3.476647e-06

3.476647e-06

3.476647e-06

0.000645101586999998

1.198228e-05

1.198228e-05

1.198228e-05

7.74062e-06

4.24166e-06

0.000633119306999998

0.000154477788

0.000154477788

0.000154477788

0.000270165815000001

0.000270165815000001

0.000270165815000001

5.74016e-07

5.74016e-07

5.74016e-07

8.64203e-07

8.64203e-07

8.64203e-07

0.00010694835

0.00010694835

0.00010694835

0.000100089135

0.000100089135

0.000100089135

7.4229072e-05

7.0694352e-05

7.0694352e-05

7.0694352e-05

7.0694352e-05

7.0694352e-05

3.53472e-06

3.53472e-06

3.53472e-06

3.53472e-06

3.53472e-06

6.15041e-05

3.534722e-06

3.534722e-06

3.534722e-06

3.534722e-06

3.534722e-06

5.7969378e-05

5.7969378e-05

5.7969378e-05

5.7969378e-05

5.7969378e-05

3.0416e-06

3.0416e-06

3.0416e-06

3.0416e-06

3.0416e-06

3.0416e-06

0.000100468957

8.96437e-07

8.96437e-07

8.96437e-07

8.96437e-07

8.96437e-07

9.957252e-05

9.957252e-05

9.957252e-05

9.957252e-05

9.957252e-05

0.00211942654300003

0.00211942654300003

0.00211942654300003

0.00211942654300003

0.00211942654300003

0.000359937536000001

0.000569106167999999

0.001190382839

0.000641067368999999

0.000183596177

4.1443816e-05

4.1443816e-05

4.1443816e-05

4.1443816e-05

0.000137721202

0.000137721202

2.6156964e-05

2.6156964e-05

0.000111564238

0.000111564238

8.96437e-07

8.96437e-07

8.96437e-07

8.96437e-07

3.534722e-06

7.06944e-07

7.06944e-07

7.06944e-07

2.827778e-06

2.827778e-06

2.827778e-06

0.000325244901

0.000325244901

0.00018858588

6.50297e-06

6.50297e-06

0.00018208291

0.00018208291

1.1996402e-05

1.1996402e-05

1.1996402e-05

9.137734e-06

9.137734e-06

8.96437e-07

8.241297e-06

3.53472e-06

3.53472e-06

3.53472e-06

0.000111990165

0.000111990165

0.000111990165

0.000131329854

0.000131329854

6.4568928e-05

5.0123734e-05

5.0123734e-05

7.069444e-06

7.069444e-06

7.37575e-06

7.37575e-06

6.3933146e-05

1.374009e-05

1.374009e-05

5.0193056e-05

5.0193056e-05

2.82778e-06

2.82778e-06

2.82778e-06

8.96437e-07

8.96437e-07

8.96437e-07

8.96437e-07

8.96437e-07

0.000124422122

7.06944e-07

7.06944e-07

7.06944e-07

7.06944e-07

7.06944e-07

9.897218e-06

9.897218e-06

9.897218e-06

9.897218e-06

9.897218e-06

0.00011381796

0.00011381796

0.00011381796

0.00011381796

0.00011381796

0.000101603337

0.000101603337

0.000101603337

0.000101603337

0.000101603337

0.000101603337

0.000101603337
