# Supplementary material for: Succession of bacterial communities on carrion is independent of vertebrate scavengers
Source: PeerJ. 2020 Jun 10;8:e9307. doi: 10.7717/peerj.9307 (PMC7293191; doi:10.7717/peerj.9307)
Supplement: Dataset S1 — Interactive Krona plots, and the full count table showing the distribution of each OTU among all samples. [file peerj-08-9307-s001.zip › DS4_COW.soil_krona.html]

Javascript must be enabled to view this page.

members
magnitude
magnitudeUnassigned

COW.soil\_krona

0.999999859829328

0.999554454219328

1.31738e-05

1.31738e-05

1.31738e-05

1.31738e-05

1.31738e-05

1.31738e-05

0.000332457829999999

1.831276e-05

1.831276e-05

1.831276e-05

1.831276e-05

1.831276e-05

2.412718e-05

2.412718e-05

2.412718e-05

2.412718e-05

2.412718e-05

0.00024840203

0.00024840203

0.00024840203

0.00024840203

0.00024840203

2.03148e-05

2.03148e-05

2.03148e-05

2.03148e-05

2.03148e-05

2.130106e-05

2.130106e-05

2.130106e-05

2.130106e-05

2.130106e-05

0.0146867268499997

4.940204e-05

4.940204e-05

6.77109e-06

6.77109e-06

5.57824e-06

1.19285e-06

4.263095e-05

4.263095e-05

4.263095e-05

8.36735e-06

8.36735e-06

8.36735e-06

8.36735e-06

8.36735e-06

1.360275e-05

1.360275e-05

1.360275e-05

1.360275e-05

1.360275e-05

0.00349775126000002

0.00349775126000002

0.00349775126000002

0.00349775126000002

0.00349775126000002

4.24056e-06

4.24056e-06

4.24056e-06

4.24056e-06

4.24056e-06

0.000905514189999997

0.000905514189999997

0.000905514189999997

0.000905514189999997

0.000905514189999997

0.00775735865000006

0.00775735865000006

0.00775735865000006

0.00775735865000006

0.00775735865000006

5.185001e-05

5.185001e-05

5.185001e-05

5.185001e-05

5.185001e-05

0.000351327199999999

0.000351327199999999

0.000351327199999999

0.000351327199999999

0.000351327199999999

1.39456e-06

1.39456e-06

1.39456e-06

1.39456e-06

1.39456e-06

0.00151547878

0.00151547878

0.000742316309999997

0.000742316309999997

0.000742316309999997

1.39456e-06

1.39456e-06

1.39456e-06

0.00028090979

0.00028090979

0.00028090979

0.000490858119999999

0.000490858119999999

0.000490858119999999

0.00014772902

0.00014772902

0.00014772902

0.00014772902

0.00014772902

9.59049e-06

9.59049e-06

9.59049e-06

9.59049e-06

9.59049e-06

5.345925e-05

5.345925e-05

5.345925e-05

5.345925e-05

5.345925e-05

0.00031966074

0.00031966074

0.00031966074

0.00031966074

0.00031966074

0.00042306778

1.203677e-05

1.203677e-05

1.203677e-05

1.203677e-05

1.203677e-05

0.00028409918

0.00028409918

0.00028409918

0.00028409918

0.00028409918

9.31296e-06

9.31296e-06

9.31296e-06

9.31296e-06

9.31296e-06

0.00011761887

0.00011761887

0.00011761887

0.00011761887

0.00011761887

4.829886e-05

8.19593e-06

8.19593e-06

8.19593e-06

8.19593e-06

8.19593e-06

4.010293e-05

4.010293e-05

4.010293e-05

4.010293e-05

4.010293e-05

0.148402550330021

0.00048899582

0.00043054933

3.536725e-05

3.536725e-05

3.536725e-05

0.00017136098

0.00017136098

0.00017136098

0.0001116211

0.0001116211

0.0001116211

0.0001122

0.0001122

0.0001122

3.66943e-06

3.66943e-06

3.66943e-06

3.66943e-06

5.477706e-05

3.924227e-05

3.924227e-05

3.924227e-05

6.97279e-06

6.97279e-06

6.97279e-06

8.562e-06

8.562e-06

8.562e-06

0.124422941040006

0.00165043083

0.00165043083

6.096351e-05

6.096351e-05

5.358836e-05

5.358836e-05

0.00153587896

0.00122505522

1.06014e-06

0.0003097636

0.117837250550004

0.117837250550004

6.96260500000001e-05

1.06014e-06

6.85659100000001e-05

2.32593e-06

2.32593e-06

0.00016079397

4.87708e-06

0.00015591689

0.000748895340000001

0.000573117290000001

0.00017577805

0.000569067029999999

2.53158e-06

0.00029745439

0.00026908106

3.18988e-05

1.22314e-06

1.463127e-05

1.604439e-05

0.00025832266

8.632358e-05

1.22314e-06

0.00017077594

1.775258e-05

1.775258e-05

0.105882397150002

0.000596223120000002

0.00906061265999989

5.914477e-05

0.00012590911

3.048176e-05

0.00088876185

0.0951212638799998

0.00267714993000003

0.00267714993000003

7.74699e-06

7.74699e-06

8.59689e-06

8.59689e-06

0.00373504161000005

0.00373504161000005

4.9236e-06

4.9236e-06

6.460073e-05

7.15712e-06

6.97279e-06

5.047082e-05

0.00359811129000003

0.00010663041

7.35068e-05

0.00281496273000001

0.000428334990000001

3.791749e-05

0.00013675887

0.0002856778

0.0002856778

0.0002856778

4.71795e-06

4.87709e-06

4.31985e-05

0.00022922645

3.65781e-06

0.00207481098

0.00207481098

0.00207481098

8.53489e-06

0.00206627609

0.000432769750000001

1.21927e-06

1.21927e-06

1.21927e-06

4.87708e-06

4.87708e-06

4.87708e-06

2.16286e-05

2.16286e-05

2.16286e-05

0.000405044800000001

0.000405044800000001

0.000405044800000001

0.00214200113

0.00214200113

0.00214200113

0.00214200113

0.000938142089999998

6.641074e-05

6.641074e-05

6.641074e-05

6.641074e-05

8.56201e-06

8.56201e-06

8.56201e-06

8.56201e-06

5.642266e-05

5.642266e-05

5.642266e-05

5.642266e-05

2.099665e-05

2.099665e-05

2.099665e-05

2.099665e-05

9.48335e-05

9.48335e-05

9.48335e-05

9.48335e-05

5.218127e-05

5.218127e-05

5.218127e-05

5.218127e-05

0.000638735259999998

0.000638735259999998

0.000638735259999998

0.000638735259999998

4.267246e-05

4.267246e-05

4.267246e-05

4.267246e-05

4.267246e-05

0.022509798919999

1.19285e-06

1.19285e-06

1.19285e-06

1.19285e-06

1.968274e-05

1.968274e-05

1.968274e-05

1.968274e-05

1.034557e-05

1.034557e-05

1.034557e-05

1.034557e-05

4.94757e-06

4.94757e-06

4.94757e-06

4.94757e-06

0.00045730784

0.00045730784

0.00045730784

0.00045730784

0.00043789171

0.00043789171

0.00043789171

0.00043789171

9.70743e-06

9.70743e-06

9.70743e-06

9.70743e-06

2.78912e-06

2.78912e-06

2.78912e-06

2.78912e-06

3.052738e-05

3.052738e-05

3.052738e-05

3.052738e-05

1.19285e-06

1.19285e-06

1.19285e-06

1.19285e-06

6.16191e-06

6.16191e-06

6.16191e-06

6.16191e-06

7.59474e-06

7.59474e-06

7.59474e-06

7.59474e-06

0.0198600840699993

0.0198600840699993

0.0198600840699993

0.0198600840699993

2.426047e-05

2.426047e-05

2.426047e-05

2.426047e-05

2.4547e-06

2.4547e-06

2.4547e-06

2.4547e-06

3.66943e-06

3.66943e-06

3.66943e-06

3.66943e-06

3.66943e-06

3.66943e-06

3.66943e-06

3.66943e-06

0.00147571184

0.00147571184

0.00147571184

0.00147571184

2.43854e-06

2.43854e-06

2.43854e-06

2.43854e-06

5.564487e-05

5.564487e-05

5.564487e-05

5.564487e-05

1.26579e-06

1.26579e-06

1.26579e-06

1.26579e-06

2.1394e-05

2.1394e-05

2.1394e-05

2.1394e-05

6.986407e-05

6.986407e-05

6.986407e-05

6.986407e-05

2.094162e-05

2.094162e-05

2.094162e-05

2.094162e-05

2.094162e-05

2.094162e-05

0.0361816586699978

0.0361816586699978

0.0361816586699978

0.0361816586699978

0.0361816586699978

0.0361816586699978

0.0443868408299985

0.0443868408299985

8.90163e-06

6.46309e-06

6.46309e-06

6.46309e-06

2.43854e-06

2.43854e-06

2.43854e-06

0.00034740098

2.687508e-05

2.687508e-05

2.687508e-05

1.214057e-05

1.214057e-05

1.214057e-05

0.00017102713

0.00017102713

0.00017102713

0.0001373582

0.0001373582

0.0001373582

8.66324799999999e-05

8.66324799999999e-05

8.66324799999999e-05

8.66324799999999e-05

0.00300436619000002

0.00156803604

0.00156803604

0.00156803604

0.00104161087

0.00104161087

0.00104161087

0.0002901483

0.0002901483

0.0002901483

0.00010457098

0.00010457098

0.00010457098

0.0191784010099974

2.43854e-06

2.43854e-06

2.43854e-06

1.21927e-06

1.21927e-06

1.21927e-06

0.0118436377199999

0.0118436377199999

0.0118436377199999

0.000629435869999998

0.000629435869999998

0.000629435869999998

0.00444330833000005

0.00444330833000005

0.00444330833000005

0.00225836128000001

0.00225836128000001

0.00225836128000001

0.0217611385399989

0.0217611385399989

0.00819595898000009

0.00819595898000009

0.00543098801000006

0.00011693489

0.00426024180000001

0.000452257779999999

3.917764e-05

0.00012711334

0.00043526256

0.00813419155000008

0.00025694768

1.22492e-06

3.67475e-06

0.00208719341000001

0.00274359415000005

7.71167399999999e-05

6.80316e-06

0.00169175786

2.44806e-06

0.00122413425

2.845902e-05

1.083755e-05

1.431165e-05

1.431165e-05

1.431165e-05

1.431165e-05

1.431165e-05

1.431165e-05

0.22253951512006

1.22314e-06

1.22314e-06

1.22314e-06

1.22314e-06

1.22314e-06

0.143191792560034

0.0235976236599985

4.140622e-05

3.582798e-05

3.582798e-05

5.57824e-06

5.57824e-06

0.000735438549999999

0.00042195709

0.00042195709

0.00029522836

0.00029522836

1.82531e-05

1.82531e-05

0.00362992783000003

3.892636e-05

3.892636e-05

2.211244e-05

2.211244e-05

0.00353246081000003

0.00353246081000003

3.642822e-05

3.642822e-05

0.00268561251000002

3.724478e-05

3.724478e-05

6.45583e-05

1.22492e-06

6.333338e-05

0.00213564921000002

7.31563e-06

0.000392353089999999

7.289052e-05

0.00166308997

0.000448160219999999

0.000438956029999999

9.20419e-06

2.78912e-06

1.39456e-06

1.39456e-06

1.39456e-06

1.39456e-06

0.00695097365000005

3.66943e-06

3.66943e-06

0.00010897164

3.75085e-06

6.272416e-05

4.249663e-05

2.963057e-05

1.996305e-05

7.21769e-06

2.44983e-06

0.00017875329

0.00017875329

1.980557e-05

1.980557e-05

0.00011712652

0.00011207415

1.21927e-06

1.39456e-06

2.43854e-06

8.73342e-06

1.22314e-06

7.51028e-06

2.32593e-06

1.06014e-06

1.26579e-06

3.65781e-06

3.65781e-06

0.00647829947000003

0.0001402099

0.00259791941000006

0.00015378743

0.00146922871

0.00211715402

0.00837923298000015

9.8741e-06

8.81396e-06

1.06014e-06

0.0045981901

0.0045981901

0.00017348956

5.75941e-06

1.025509e-05

0.00015747506

0.00141347169

1.469855e-05

3.477572e-05

0.00040498485

6.22655e-06

0.000642695119999999

1.673471e-05

1.39456e-06

0.0002539263

3.803533e-05

0.00031377189

2.041704e-05

9.277362e-05

0.00020058123

0.000750566069999997

0.000400139579999999

2.43854e-06

0.000347987949999999

0.00111503403

5.06932e-06

1.26579e-06

4.922924e-05

0.000369698599999999

0.000689771079999999

4.83554e-06

4.83554e-06

9.73166e-06

7.28537e-06

7.28537e-06

2.44629e-06

2.44629e-06

0.00116251114

0.00116251114

0.00116251114

6.192662e-05

2.336467e-05

2.336467e-05

2.336467e-05

3.489252e-05

3.489252e-05

3.489252e-05

3.66943e-06

3.66943e-06

3.66943e-06

0.0413968226800008

8.54264e-06

8.54264e-06

8.54264e-06

0.00130258928

0.00109135983

0.00107422291

1.26579e-06

6.24721e-06

2.44629e-06

7.17763e-06

2.316615e-05

9.75417e-06

1.341198e-05

1.377598e-05

1.377598e-05

0.00015424678

0.00015424678

2.004054e-05

3.63704e-06

1.64035e-05

6.45855e-06

6.45855e-06

6.45855e-06

0.0127744495799996

0.0127744495799996

0.0127744495799996

1.06014e-06

1.06014e-06

1.06014e-06

2.61948e-06

2.61948e-06

2.61948e-06

0.0199624884799997

2.902907e-05

2.902907e-05

0.000403061489999999

0.000403061489999999

2.181177e-05

4.89258e-06

1.20421e-05

4.87709e-06

0.00017004006

1.22314e-06

0.00016628534

2.53158e-06

2.474973e-05

1.22492e-06

2.352481e-05

5.23718e-06

2.44806e-06

2.78912e-06

1.153318e-05

1.153318e-05

0.0192970259999999

4.80348e-06

9.41698e-06

1.834607e-05

0.0004221165

0.01565101665

7.46382e-06

3.66943e-06

1.19285e-06

4.18368e-06

1.075686e-05

8.736152e-05

0.00307162886

2.44983e-06

2.61947e-06

0.00028183218

0.00016804252

0.00016804252

0.00011378966

0.00011378966

0.00671133804000002

0.00613288844

0.00613288844

1.13348e-05

1.13348e-05

0.000567114799999999

0.000567114799999999

0.00034544431

5.528396e-05

5.528396e-05

0.00026846973

0.00026846973

2.169062e-05

2.169062e-05

1.62011e-05

1.62011e-05

1.62011e-05

1.62011e-05

0.0536350788299991

1.762219e-05

1.762219e-05

1.762219e-05

9.243253e-05

5.73351e-05

5.73351e-05

3.509743e-05

3.509743e-05

0.000440671239999999

0.000363895609999999

3.57856e-06

4.058023e-05

0.00031973682

5.06932e-06

5.06932e-06

8.57442e-06

8.57442e-06

5.578239e-05

5.578239e-05

7.3495e-06

4.89967e-06

2.44983e-06

0.00015796573

0.000148183

0.00014141191

6.77109e-06

2.43854e-06

2.43854e-06

7.34419e-06

7.34419e-06

0.0117674475999997

5.813647e-05

5.813647e-05

0.00134654253

0.00134654253

0.00101017403

0.00101017403

0.00880926875999988

0.00880926875999988

1.542427e-05

1.542427e-05

0.00052790154

0.00052790154

0.000402850349999999

6.415796e-05

6.415796e-05

0.00033869239

0.00033869239

0.00118004372

2.375024e-05

2.255739e-05

1.19285e-06

0.000952311879999998

0.000871480669999999

7.956542e-05

1.26579e-06

1.22492e-06

1.22492e-06

0.00020275668

0.00020275668

1.22314e-06

1.22314e-06

1.22314e-06

7.743896e-05

4.601354e-05

4.601354e-05

3.142542e-05

3.142542e-05

3.156259e-05

2.302769e-05

5.99455e-06

1.703314e-05

8.5349e-06

8.5349e-06

3.070353e-05

3.070353e-05

3.070353e-05

0.00041152084

0.00017652088

1.552705e-05

2.93629e-05

0.00013040601

1.22492e-06

4.968214e-05

4.689302e-05

1.39456e-06

1.39456e-06

9.80598e-06

2.3857e-06

7.42028e-06

0.00017551184

0.00017551184

0.00387288498000002

3.607951e-05

4.89612e-06

3.118339e-05

0.000646468799999999

0.000646468799999999

5.06931e-06

2.61948e-06

2.44983e-06

5.20511e-06

5.20511e-06

6.09636e-06

6.09636e-06

0.00022313539

4.512124e-05

8.727989e-05

2.572322e-05

6.36084e-06

5.86502e-05

0.00295083050000001

0.00010649197

3.112998e-05

0.00262558435

2.382539e-05

0.00016379881

1.472798e-05

1.472798e-05

1.472798e-05

0.0126954153399997

0.0126954153399997

0.0126954153399997

9.07626e-06

9.07626e-06

9.07626e-06

2.28329e-06

2.28329e-06

2.28329e-06

9.385785e-05

4.53627e-06

4.53627e-06

2.555711e-05

1.803797e-05

7.51914e-06

6.376447e-05

6.376447e-05

0.00320526641000001

0.000370209819999999

5.319538e-05

1.079488e-05

0.00030621956

1.765772e-05

1.765772e-05

0.000416782319999999

0.000416782319999999

4.410913e-05

2.44984e-06

3.57856e-06

4.89258e-06

3.318815e-05

0.00011203035

2.335128e-05

3.179186e-05

5.688721e-05

4.89966e-06

2.44983e-06

2.44983e-06

4.05491e-06

4.05491e-06

0.00223552250000001

1.101893e-05

5.868411e-05

2.53158e-06

1.44999e-05

9.761706e-05

8.934003e-05

1.22492e-06

0.00191321765999999

1.39456e-06

2.78912e-06

2.53158e-06

4.067305e-05

0.000699772859999998

1.06014e-06

1.06014e-06

0.00021375407

0.00013964282

7.411125e-05

9.861237e-05

2.167753e-05

7.34241e-06

6.959243e-05

0.0001756478

2.837879e-05

9.30501e-06

0.000137964

0.00021069848

0.00021069848

0.0183473745699993

0.00185919188

0.00026668921

2.708789e-05

0.000840977549999997

1.22314e-06

0.000454742519999999

5.57823e-06

2.78912e-06

0.00025154222

8.562e-06

0.00141967407

0.00141967407

1.21927e-06

1.21927e-06

0.0150672893499997

3.380183e-05

0.0136142303899997

1.22492e-06

3.17826e-05

0.00011613911

0.00100953852

0.00026057198

9.79225e-06

4.89967e-06

4.89967e-06

4.89258e-06

4.89258e-06

7.314462e-05

2.65512e-05

2.65512e-05

4.659342e-05

4.659342e-05

0.00797612600000015

3.784775e-05

1.22314e-06

1.22314e-06

3.662461e-05

1.22314e-06

3.540147e-05

0.00793827825000015

0.000922890119999999

5.877996e-05

0.000636912940000001

2.914468e-05

5.23895e-06

0.00016859914

2.421445e-05

1.766271e-05

1.766271e-05

0.000677784639999999

0.00023212968

6.12281e-06

1.262933e-05

6.534094e-05

0.00036156188

4.044222e-05

4.044222e-05

0.000714211289999998

0.000714211289999998

0.000690006959999999

0.00023100766

3.66943e-06

2.6177e-06

2.78912e-06

4.283978e-05

0.00040708327

0.00487528031000005

0.00045405796

0.00101616606

1.22492e-06

0.00320223522000002

2.655137e-05

2.767451e-05

5.942752e-05

8.794275e-05

0.00368868169000003

4.649773e-05

4.649773e-05

4.649773e-05

4.18367e-06

4.18367e-06

4.18367e-06

2.636785e-05

1.26579e-06

1.26579e-06

2.231294e-05

2.231294e-05

2.78912e-06

2.78912e-06

6.906697e-05

1.309029e-05

1.309029e-05

6.80138e-06

6.80138e-06

4.18368e-06

4.18368e-06

1.534015e-05

1.534015e-05

3.66943e-06

3.66943e-06

2.598204e-05

2.598204e-05

0.00301237546000002

6.11572e-06

6.11572e-06

7.51914e-06

7.51914e-06

1.100829e-05

1.100829e-05

1.836489e-05

1.836489e-05

8.333962e-05

8.333962e-05

0.000554737149999999

0.000554737149999999

7.76224e-06

7.76224e-06

0.00012551024

0.00012551024

0.00218961918000002

0.00218961918000002

7.33885e-06

7.33885e-06

1.06014e-06

1.06014e-06

6.347763e-05

6.068851e-05

6.068851e-05

2.78912e-06

2.78912e-06

0.00025141379

1.39456e-06

1.39456e-06

2.060233e-05

1.22314e-06

1.274568e-05

6.63351e-06

0.00017869649

1.780966e-05

0.00014165911

1.922772e-05

5.072041e-05

3.317303e-05

1.754738e-05

6.30395e-06

4.18367e-06

4.18367e-06

2.12028e-06

2.12028e-06

5.06399e-06

5.06399e-06

1.39456e-06

3.66943e-06

0.00020393065

8.73342e-06

8.73342e-06

6.484945e-05

4.952955e-05

3.79737e-06

4.18367e-06

7.33886e-06

0.00013034778

0.00013034778

0.0119285036499997

0.0119285036499997

0.000906740759999997

0.000906740759999997

2.735478e-05

2.735478e-05

7.3944e-05

7.3944e-05

7.31563e-06

7.31563e-06

1.21927e-06

1.21927e-06

0.0108733956799998

4.89612e-06

1.698844e-05

0.00356249213000006

1.584733e-05

0.00727317166000003

3.853353e-05

3.48662e-05

1.21927e-06

2.44806e-06

0.000890828329999998

0.000890828329999998

0.000890828329999998

0.000890828329999998

0.00931296545000003

1.26579e-06

1.26579e-06

1.26579e-06

1.26579e-06

9.4359e-06

9.4359e-06

7.31562e-06

7.31562e-06

2.12028e-06

1.06014e-06

1.06014e-06

0.00011312839

1.02994e-05

1.02994e-05

1.02994e-05

1.617347e-05

1.617347e-05

1.617347e-05

4.036969e-05

4.036969e-05

4.036969e-05

4.628583e-05

4.628583e-05

4.628583e-05

2.478368e-05

2.478368e-05

2.478368e-05

1.744127e-05

7.34241e-06

2.565263e-05

2.442949e-05

2.442949e-05

2.442949e-05

1.22314e-06

1.22314e-06

1.22314e-06

2.118191e-05

1.783671e-05

1.783671e-05

1.783671e-05

3.3452e-06

2.12028e-06

2.12028e-06

1.22492e-06

1.22492e-06

0.000721189939999998

0.00013363015

0.00013119161

0.0001062578

2.493381e-05

2.43854e-06

2.43854e-06

0.0005471805

0.0001768294

0.00010806543

7.18959e-06

6.157438e-05

2.19636e-05

1.837188e-05

3.59172e-06

0.00033624942

6.11572e-06

8.93586e-06

2.53158e-06

3.66943e-06

1.06014e-06

3.84085e-06

0.00031009584

1.213808e-05

1.213808e-05

4.037929e-05

4.037929e-05

4.037929e-05

0.00328210811000002

0.00026641605

4.18367e-06

4.18367e-06

6.02839e-06

6.02839e-06

1.22492e-06

1.22492e-06

0.00023021601

0.00023021601

1.39456e-06

1.39456e-06

2.33685e-05

2.33685e-05

5.23541e-06

5.23541e-06

5.23541e-06

0.00153601535999999

0.00028759898

0.0002049487

2.495916e-05

2.3857e-06

5.530542e-05

0.00120386698

1.22492e-06

6.9728e-06

1.22492e-06

1.398471e-05

4.97312e-06

0.00012283673

0.00105264978

2.970025e-05

2.970025e-05

1.484915e-05

2.44629e-06

1.240286e-05

1.21927e-06

1.21927e-06

1.21927e-06

0.00147322202

1.35981e-05

1.35981e-05

5.39418e-05

5.39418e-05

6.141528e-05

1.06014e-06

2.76144e-05

3.274074e-05

1.22314e-06

1.22314e-06

4.44621e-06

4.44621e-06

1.520105e-05

6.27939e-06

4.9094e-06

1.22314e-06

2.78912e-06

2.690865e-05

4.89967e-06

1.220965e-05

9.79933e-06

0.00030478037

3.53861e-05

0.00026694444

2.44983e-06

2.78912e-06

2.78912e-06

1.22492e-06

1.22492e-06

0.000608067399999999

0.000608067399999999

6.397048e-05

2.61383e-06

2.44629e-06

3.625463e-05

2.265573e-05

0.0003156555

2.011361e-05

0.00029554189

0.000665683209999999

0.000665683209999999

0.000665683209999999

0.000665683209999999

0.00018983319

0.00018983319

0.0001313026

0.0001313026

1.4221e-05

1.4221e-05

4.18367e-06

4.18367e-06

4.012592e-05

1.89869e-05

2.113902e-05

0.000441358379999999

7.51914e-06

7.51914e-06

7.51914e-06

3.50642e-06

3.50642e-06

3.50642e-06

0.000427882979999999

0.000425093859999999

0.00039511227

1.871127e-05

1.127032e-05

2.78912e-06

2.78912e-06

2.44984e-06

2.44984e-06

2.44984e-06

0.0007763874

0.00067845316

0.00067845316

1.22314e-06

0.00063484741

2.54642e-05

1.06014e-06

6.09636e-06

9.76191e-06

9.793424e-05

8.57442e-06

8.57442e-06

7.546125e-05

2.48893e-06

6.03801e-06

6.693431e-05

1.389857e-05

8.30038e-06

1.19285e-06

4.40534e-06

1.39456e-06

1.39456e-06

1.39456e-06

1.39456e-06

0.000714223299999998

0.000714223299999998

0.000714223299999998

0.00012056174

2.3857e-06

0.00059127586

0.00116877401

0.00116877401

6.503001e-05

6.503001e-05

0.001103744

1.39456e-06

7.31563e-06

5.400066e-05

7.039181e-05

4.87709e-06

0.0001358385

0.000827136629999999

2.78912e-06

0.00115656505

2.411156e-05

2.044035e-05

2.044035e-05

2.44629e-06

2.44629e-06

1.22492e-06

1.22492e-06

8.065294e-05

7.704797e-05

1.19285e-06

7.585512e-05

1.21927e-06

1.21927e-06

2.3857e-06

2.3857e-06

0.00105180055

0.00105180055

0.00105180055

0.00102216464

0.00102216464

0.00102216464

0.00102216464

0.00102216464

0.0191687439999987

0.00265112584000002

5.519085e-05

5.519085e-05

5.519085e-05

1.39456e-06

1.39456e-06

1.39456e-06

4.18368e-06

4.18368e-06

4.18368e-06

0.00230451973000002

0.00230451973000002

0.00230451973000002

0.0001790475

0.0001790475

0.0001790475

4.503081e-05

4.503081e-05

4.503081e-05

4.712381e-05

4.712381e-05

4.712381e-05

1.46349e-05

1.46349e-05

1.46349e-05

6.990775e-05

6.990775e-05

2.43854e-06

2.43854e-06

6.746921e-05

2.44629e-06

5.099301e-05

1.402991e-05

0.0145023966899996

0.00018078083

8.573184e-05

8.573184e-05

1.21927e-06

1.21927e-06

9.382972e-05

3.106709e-05

6.276263e-05

0.00015259255

0.00015259255

0.00015259255

0.00213108856000001

7.31563e-06

7.31563e-06

6.415948e-05

6.415948e-05

5.598753e-05

1.994682e-05

2.85739e-05

7.46681e-06

0.00200362592

1.19285e-06

6.46033e-06

0.00199597274

0.00564835990000016

3.963922e-05

3.963922e-05

4.18367e-06

4.18367e-06

0.00159622427

0.00159622427

0.00335226874000003

0.00320220708000003

1.310533e-05

0.00013695633

0.00014724627

5.657798e-05

9.066829e-05

1.22314e-06

1.22314e-06

1.39456e-06

1.39456e-06

0.00033392522

2.4547e-06

0.00033147052

0.00012077642

0.00012077642

5.147839e-05

5.147839e-05

0.00638957485000011

5.06366e-06

5.06366e-06

0.00017471012

0.00010028474

6.500275e-05

9.42263e-06

2.43854e-06

2.43854e-06

3.485808e-05

2.44984e-06

2.43854e-06

2.99697e-05

2.3857e-06

2.3857e-06

0.00037022033

0.000254934

8.75688e-05

2.771753e-05

0.00045404525

0.00045404525

1.634066e-05

1.634066e-05

3.71562e-06

3.71562e-06

0.00501277393000007

0.00501277393000007

0.00031302296

0.00028670856

2.63144e-05

7.17586e-06

7.17586e-06

7.17586e-06

7.17586e-06

0.00011703005

0.00011703005

8.392671e-05

8.392671e-05

1.950832e-05

1.950832e-05

3.65781e-06

3.65781e-06

3.84085e-06

3.84085e-06

6.09636e-06

6.09636e-06

0.000539964609999998

0.000539964609999998

0.000539964609999998

0.000539964609999998

7.19176799999999e-05

3.366544e-05

3.366544e-05

3.366544e-05

3.825224e-05

3.825224e-05

3.825224e-05

2.762263e-05

2.762263e-05

2.518409e-05

2.518409e-05

2.43854e-06

2.43854e-06

0.00117793346

0.00117426403

0.000774115869999998

0.000387343359999999

1.39456e-06

2.933037e-05

0.00010272

0.00025332758

2.44419e-06

2.44419e-06

0.0001680501

0.0001228671

4.5183e-05

0.00022965387

0.00020608214

2.357173e-05

3.66943e-06

3.66943e-06

3.66943e-06

3.66943e-06

3.66943e-06

3.66943e-06

3.66943e-06

0.00019041125

1.06014e-06

1.06014e-06

1.06014e-06

1.06014e-06

2.473423e-05

2.473423e-05

2.473423e-05

2.473423e-05

0.00016461688

0.00016461688

0.00016461688

0.00016461688

0.0496522140799983

4.451791e-05

2.3857e-06

2.3857e-06

2.3857e-06

4.213221e-05

1.237961e-05

1.237961e-05

1.707607e-05

1.707607e-05

1.267653e-05

1.267653e-05

0.00352245693000004

0.00038448648

0.00038448648

0.00038448648

0.00179719176

0.00179719176

0.00179719176

1.22492e-06

1.22492e-06

1.22492e-06

2.413764e-05

2.413764e-05

2.413764e-05

1.26579e-06

1.26579e-06

1.26579e-06

6.77214e-06

6.77214e-06

6.77214e-06

1.381955e-05

1.381955e-05

1.381955e-05

2.303955e-05

2.303955e-05

2.303955e-05

0.00117284977

0.00022424974

0.00022424974

0.000638294959999999

0.000638294959999999

1.202516e-05

1.202516e-05

0.00024189817

0.00024189817

1.153318e-05

1.153318e-05

4.484856e-05

4.484856e-05

1.064335e-05

1.064335e-05

1.064335e-05

8.580284e-05

8.580284e-05

8.580284e-05

1.22314e-06

1.22314e-06

1.22314e-06

5.96426e-06

5.96426e-06

5.96426e-06

5.96426e-06

6.23591e-06

6.23591e-06

6.23591e-06

6.23591e-06

2.490032e-05

2.44629e-06

2.44629e-06

2.44629e-06

2.245403e-05

2.245403e-05

2.245403e-05

1.067847e-05

1.067847e-05

1.067847e-05

8.29277e-06

2.3857e-06

0.0388066279699985

0.00015215781

6.642837e-05

6.642837e-05

1.830839e-05

1.830839e-05

6.742105e-05

6.742105e-05

5.761266e-05

5.761266e-05

5.761266e-05

0.00017212681

1.591506e-05

1.591506e-05

3.145441e-05

3.145441e-05

1.22314e-06

1.22314e-06

1.21927e-06

1.21927e-06

7.77934e-06

7.77934e-06

0.00011453559

0.00011453559

0.0123762168399996

0.0123762168399996

0.0123762168399996

0.00015178308

0.00015178308

0.00015178308

0.00356330713000003

3.90987e-05

3.90987e-05

0.00352298529000003

0.00191428917

0.00147721981

3.764044e-05

9.383587e-05

1.22314e-06

1.22314e-06

0.000889175099999998

0.00026856133

0.00026856133

2.854494e-05

2.854494e-05

0.000592068829999999

0.000592068829999999

0.00226624928000002

1.39456e-06

1.39456e-06

0.00020833839

0.00020833839

0.00111634119

0.00111634119

1.781172e-05

1.781172e-05

0.000902764739999997

0.000902764739999997

1.959868e-05

1.959868e-05

0.00044019536

6.274754e-05

6.274754e-05

3.868715e-05

3.868715e-05

1.07844e-05

1.07844e-05

0.00032797627

0.00032797627

2.43854e-06

2.43854e-06

2.43854e-06

0.00218980152000001

0.00035143464

8.799313e-05

0.00026344151

0.000810976049999999

8.79683e-06

0.00018460014

0.000617579079999999

0.000765396459999998

0.000765396459999998

0.00026199437

0.00026199437

0.000701639649999998

4.594829e-05

4.594829e-05

0.00047069791

0.00047069791

0.00016810833

0.00016810833

2.12028e-06

2.12028e-06

1.476484e-05

1.476484e-05

0.00365561730000002

0.000688325899999999

0.00031140685

0.00037691905

1.502055e-05

1.135112e-05

3.66943e-06

0.00012641371

1.39456e-06

0.00012501915

0.000841148829999999

3.861505e-05

0.000623991199999999

1.068228e-05

9.60694e-06

0.00015825336

0.000674564469999998

0.000674564469999998

0.00131014384

8.022986e-05

0.000781332889999999

0.00044858109

0.00456491741000006

0.00456491741000006

3.237917e-05

0.00011804499

0.00016652687

1.448516e-05

0.00031954821

0.00385929727000001

4.626839e-05

1.39456e-06

6.97279e-06

0.0009864358

0.0009864358

2.12028e-06

0.00021495397

7.75034e-05

9.041973e-05

0.00022665209

0.00037478633

4.731578e-05

1.376715e-05

1.376715e-05

2.675759e-05

2.675759e-05

6.79104e-06

6.79104e-06

0.00541486642000012

4.57498e-06

4.57498e-06

7.017363e-05

5.150622e-05

7.6901e-06

1.097731e-05

0.00050817222

0.00050817222

0.00483194559000009

4.89789e-06

0.0005634744

9.06075e-06

0.000681135839999999

0.00357337671000002

0.00117477148

1.271524e-05

1.271524e-05

0.0002431861

1.22314e-06

0.00020812908

3.065346e-05

3.18042e-06

0.000451493989999999

0.000437891219999999

1.081365e-05

2.78912e-06

0.000464758449999999

0.000464758449999999

2.6177e-06

2.6177e-06

0.00102683735

4.89612e-06

4.89612e-06

4.89612e-06

2.78912e-06

2.78912e-06

2.78912e-06

2.44629e-06

2.44629e-06

2.44629e-06

0.000942027489999998

0.000942027489999998

0.000942027489999998

3.18042e-06

3.18042e-06

3.18042e-06

6.782848e-05

6.782848e-05

6.782848e-05

3.66943e-06

3.66943e-06

3.66943e-06

0.000446047939999999

0.000446047939999999

0.000446047939999999

0.000446047939999999

0.00246789992000002

1.19285e-06

1.19285e-06

1.19285e-06

0.00246670707000002

0.00172078694

0.00012887067

0.00159191627

0.000745920129999998

0.00018123662

0.000564683509999999

0.00282407814000003

0.00110768625

8.562e-06

8.562e-06

0.00106919434

1.654342e-05

0.000814845109999998

3.580556e-05

5.786242e-05

0.00014413783

4.18367e-06

4.18367e-06

2.574624e-05

1.39456e-06

2.435168e-05

2.44629e-06

2.44629e-06

2.44629e-06

0.0017139456

0.00138787143

1.934853e-05

1.21927e-06

0.000863589629999998

1.39456e-06

3.65781e-06

2.432174e-05

0.00047433989

0.000326074169999999

3.44808e-05

0.00021307935

1.661221e-05

6.190181e-05

0.000465968959999999

0.000465968959999999

0.000465968959999999

0.00010669736

3.867054e-05

0.000320601059999999

0.000732168279999999

0.000732168279999999

0.000732168279999999

0.000732168279999999

7.382408e-05

1.957032e-05

5.425376e-05

0.000654674769999999

1.590776e-05

0.000638767009999999

3.66943e-06

3.66943e-06

1.06014e-06

1.06014e-06

1.06014e-06

1.06014e-06

1.06014e-06

1.06014e-06

4.179708e-05

2.415886e-05

2.415886e-05

2.415886e-05

2.415886e-05

2.415886e-05

1.763822e-05

1.763822e-05

1.763822e-05

1.763822e-05

1.763822e-05

0.000396995039999999

0.00018713818

0.00018713818

0.00018713818

0.00018713818

0.00018713818

2.78912e-06

2.78912e-06

2.78912e-06

2.78912e-06

2.78912e-06

0.00020706774

0.00020706774

0.00020706774

0.00020706774

0.00020706774

8.44626e-06

7.18047e-06

7.18047e-06

7.18047e-06

7.18047e-06

7.18047e-06

1.26579e-06

1.26579e-06

1.26579e-06

1.26579e-06

1.26579e-06

0.00304505209000002

1.920424e-05

1.920424e-05

1.920424e-05

1.920424e-05

1.920424e-05

3.65781e-06

3.65781e-06

3.65781e-06

3.65781e-06

3.65781e-06

0.00013927759

0.00013927759

0.00013927759

0.00013927759

0.00013927759

0.00107687897

0.00107687897

0.00107687897

0.00107687897

0.00107687897

2.43854e-06

2.43854e-06

2.43854e-06

2.43854e-06

2.43854e-06

0.00180359494

0.00180359494

0.00180359494

0.00180359494

0.00180359494

0.0019952874

0.0019952874

7.05081e-06

7.05081e-06

7.05081e-06

7.05081e-06

1.26579e-06

1.26579e-06

1.26579e-06

1.26579e-06

0.0019869708

0.00179610888

0.00179610888

0.000588022779999998

1.26579e-06

0.000620979619999999

0.00017683661

0.00040900408

0.00019086192

4.947691e-05

4.808235e-05

1.39456e-06

0.00014032487

3.63139e-06

1.001074e-05

6.10023e-06

2.1139e-05

2.3857e-06

5.419183e-05

4.286598e-05

1.06014e-06

1.06014e-06

2.987471e-05

2.987471e-05

2.987471e-05

2.987471e-05

2.987471e-05

2.987471e-05

0.0492086627100004

0.00151577958

0.00151577958

0.00151577958

0.00151577958

0.00151577958

0.00185294271999999

1.223144e-05

1.223144e-05

1.223144e-05

1.223144e-05

0.00184071127999999

0.00184071127999999

0.00183948813999999

9.582903e-05

0.000762437539999999

0.00098122157

1.22314e-06

1.22314e-06

8.80408e-05

1.970327e-05

1.970327e-05

1.970327e-05

1.970327e-05

6.833753e-05

6.833753e-05

6.833753e-05

6.833753e-05

0.0390520219000004

0.0390520219000004

0.000722756689999999

0.000722756689999999

0.000722756689999999

0.00978091547

1.559498e-05

1.559498e-05

0.00013730458

0.00013730458

0.00872185079000014

0.00872185079000014

0.000906165119999999

0.000906165119999999

0.00430598590000003

0.00430598590000003

0.00430598590000003

7.7554e-06

7.7554e-06

7.7554e-06

0.024201811949999

0.024201811949999

0.00494084112000001

0.000715210399999998

0.00142785215

0.00830567904000005

0.00881222924

2.43854e-06

2.43854e-06

2.43854e-06

1.151624e-05

1.151624e-05

2.78912e-06

8.72712e-06

1.884171e-05

1.884171e-05

1.884171e-05

0.00165150918

0.00165150918

0.00165150918

6.210356e-05

2.027714e-05

4.182642e-05

0.00014702558

0.0001105841

3.644148e-05

1.993233e-05

4.00839e-06

1.592394e-05

1.637638e-05

1.39456e-06

1.498182e-05

0.00013201182

0.00013201182

0.00046305232

0.00017120698

6.962773e-05

0.00022082305

1.39456e-06

0.000811007189999998

2.235944e-05

7.84149e-06

0.000595463519999999

0.00018534274

0.00503858338000011

0.00023135305

0.00023135305

0.00023135305

0.00023135305

0.00036298928

0.00036298928

0.00036298928

0.00036298928

0.00362289433000002

0.00362289433000002

0.00362289433000002

0.00362289433000002

0.000568288549999999

0.000568288549999999

0.000568288549999999

0.000568288549999999

0.00011146572

0.00011146572

0.00011146572

0.00011146572

7.2744e-06

7.2744e-06

7.2744e-06

7.2744e-06

9.664877e-05

9.664877e-05

9.664877e-05

7.395812e-05

2.269065e-05

3.766928e-05

3.766928e-05

3.766928e-05

3.766928e-05

9.78515e-06

9.78515e-06

9.78515e-06

9.78515e-06

3.66943e-06

6.11572e-06

0.085035629740014

0.0485247568599996

0.0485247568599996

0.000773124500000001

0.00050081985

0.00050081985

2.994214e-05

2.994214e-05

0.00024236251

0.00024236251

2.43854e-06

2.43854e-06

2.43854e-06

0.00010723082

3.838171e-05

3.838171e-05

6.884911e-05

6.884911e-05

4.18367e-06

4.18367e-06

4.18367e-06

0.00354654994000002

5.208729e-05

5.208729e-05

0.00017230261

0.00017230261

1.950834e-05

1.950834e-05

0.00218081758000001

0.00218081758000001

0.00112183412

0.00112183412

1.06014e-06

1.06014e-06

1.06014e-06

0.00186613518

0.00186613518

0.00186613518

8.40673200000001e-05

2.27941e-06

1.06014e-06

1.21927e-06

8.17879100000001e-05

8.17879100000001e-05

0.00502762542000002

0.00200105777

1.22492e-06

3.67475e-06

0.00192925853

2.943328e-05

5.23541e-06

3.117074e-05

1.06014e-06

0.00048901811

2.43854e-06

0.0002903877

0.00019619187

0.00242142650000003

0.00242142650000003

0.00011612304

8.36735e-06

2.44629e-06

5.57823e-06

1.27217e-05

2.370745e-05

6.330202e-05

5.761956e-05

4.831501e-05

4.831501e-05

9.30455e-06

9.30455e-06

1.658652e-05

1.100829e-05

1.100829e-05

5.57823e-06

5.57823e-06

0.0370329604299998

0.00024602455

0.00018665663

5.936792e-05

0.0137343485199998

4.93521e-06

1.433026e-05

8.299479e-05

0.00641357173000001

0.00173708081

1.19285e-06

9.48242e-06

2.570408e-05

0.00544505637000001

0.00021598989

0.00016262755

5.336234e-05

0.00217817660000001

0.00023500282

2.852602e-05

0.0018853384

2.179199e-05

2.44806e-06

1.39456e-06

3.67475e-06

2.43854e-06

2.43854e-06

0.00502819447000006

2.44628e-06

1.26579e-06

0.00015116468

9.2391e-06

1.993785e-05

3.70433e-06

0.00115958897

0.00028744318

0.00333622130000003

5.718299e-05

7.436158e-05

7.174388e-05

2.6177e-06

9.211591e-05

4.900707e-05

4.310884e-05

0.00512256813000008

7.42593e-06

0.00016898238

1.39456e-06

1.06014e-06

1.26579e-06

0.00426319430999999

0.00029155339

1.39456e-06

0.00013665687

1.22314e-06

6.361456e-05

3.57856e-06

2.570216e-05

0.00013774452

1.166155e-05

6.11571e-06

0.00930299651999991

0.00930299651999991

0.00024454924

2.528894e-05

2.44628e-06

2.570643e-05

0.00019110759

0.000606031419999998

0.00010159203

1.22492e-06

0.00050321447

0.00018516506

1.165226e-05

0.0001735128

5.17482e-06

5.17482e-06

5.17482e-06

2.823566e-05

2.823566e-05

2.44628e-06

2.44628e-06

2.44628e-06

3.78026e-06

3.78026e-06

3.78026e-06

3.65149e-06

2.3857e-06

2.3857e-06

1.26579e-06

1.26579e-06

8.53489e-06

8.53489e-06

6.09635e-06

2.43854e-06

7.70246e-06

7.70246e-06

1.26579e-06

6.43667e-06

2.12028e-06

2.12028e-06

1.06014e-06

1.06014e-06

4.87708e-06

4.87708e-06

4.87708e-06

4.87708e-06

4.87708e-06

0.00384891202000002

0.00384891202000002

0.00242326525000001

4.56834e-06

4.56834e-06

6.09636e-06

6.09636e-06

1.21927e-06

1.21927e-06

1.06014e-06

1.06014e-06

9.79225e-06

1.22492e-06

8.56733e-06

0.00202904001

2.390674e-05

0.00192767024

1.21927e-06

1.991513e-05

1.081718e-05

3.67475e-06

4.18367e-05

5.717692e-05

5.717692e-05

0.00029719178

2.234793e-05

0.00027484385

1.589704e-05

8.53489e-06

7.36215e-06

1.22314e-06

1.22314e-06

0.00053519743

1.21927e-06

1.21927e-06

3.486394e-05

3.486394e-05

0.00049911422

0.00049911422

0.000887999509999998

2.28328e-06

2.28328e-06

1.220821e-05

2.44629e-06

9.76192e-06

0.000873508019999999

0.0002419764

1.06014e-06

1.133176e-05

0.000619139719999999

2.44983e-06

2.44983e-06

2.44983e-06

0.0311286654999977

4.71795e-06

1.06014e-06

1.06014e-06

1.06014e-06

3.65781e-06

3.65781e-06

3.65781e-06

0.000380559959999999

0.000380559959999999

2.03612e-05

2.03612e-05

8.927677e-05

7.09877e-05

1.828907e-05

0.00010432668

3.72443e-06

7.185086e-05

2.875139e-05

0.00012056215

0.00012056215

4.603316e-05

4.603316e-05

9.17322399999999e-05

9.17322399999999e-05

9.17322399999999e-05

9.17322399999999e-05

0.0306516553499977

0.00111300008

0.0010688213

0.00101952111

4.790563e-05

1.39456e-06

4.295386e-05

4.295386e-05

1.22492e-06

1.22492e-06

0.0269782913199983

0.00016497557

9.26762e-06

0.00015570795

0.00271756230000004

1.22314e-06

4.97399e-06

0.00207721314000001

0.00010425456

9.04596e-06

9.687884e-05

0.00033690564

1.603191e-05

5.104612e-05

1.256486e-05

2.53158e-06

4.89256e-06

0.00576111400000007

0.00235331793000001

2.28328e-06

1.21927e-06

0.00044684703

0.00295744649000003

0.00014245663

2.976129e-05

1.39456e-06

0.00011130078

0.000692399089999997

0.00015979711

6.28326e-06

0.00039518655

0.00013113217

0.00596308940000004

1.39456e-06

9.75417e-06

0.00046985631

0.00126517218

0.00103835534

1.22314e-06

4.085513e-05

0.00011837295

0.00297986124000003

3.824438e-05

0.000915618829999999

0.00041414145

3.572071e-05

0.000321345149999999

0.00014441152

0.00027222328

0.000198277

2.675787e-05

2.43854e-06

4.474987e-05

0.00536068663000005

0.00536068663000005

0.00314475683000004

0.0001779774

0.00237071339000004

2.44629e-06

0.00024014548

1.22314e-06

1.22492e-06

2.44628e-06

0.000348579929999999

0.00020942111

2.48506e-06

0.00020693605

0.000400056819999999

0.0001691281

2.12028e-06

0.00022880844

0.00051928314

1.975474e-05

5.577031e-05

0.00017990014

1.22492e-06

0.00024670905

1.592398e-05

0.000352747759999999

1.637448e-05

0.00016716298

5.57823e-06

0.00016363207

3.796959e-05

3.478917e-05

3.18042e-06

1.394558e-05

2.78912e-06

1.115646e-05

0.00027917036

7.384238e-05

0.00020532798

3.08144e-05

3.08144e-05

0.000398180340000001

7.498027e-05

7.498027e-05

0.00030847579

0.00030847579

1.472428e-05

1.472428e-05

0.00025600218

0.00012205868

4.18367e-06

0.00011787501

0.00012575144

1.06014e-06

1.21927e-06

0.00012347203

1.21927e-06

1.21927e-06

4.18367e-06

4.18367e-06

2.78912e-06

2.78912e-06

0.00190618143

0.00190618143

0.00190618143

4.89258e-06

4.89258e-06

4.89258e-06

4.89258e-06

4.89258e-06

0.00149529003999999

0.00149529003999999

0.00149529003999999

0.00149529003999999

0.00149529003999999

0.00013630972

0.00013630972

0.00013630972

1.955628e-05

1.955628e-05

1.955628e-05

1.045531e-05

4.18367e-06

4.18367e-06

6.27164e-06

6.27164e-06

9.949675e-05

3.484997e-05

3.362683e-05

1.22314e-06

2.44629e-06

2.44629e-06

6.220049e-05

6.220049e-05

6.80138e-06

4.18368e-06

4.18368e-06

2.6177e-06

1.22314e-06

1.39456e-06

0.0656874632899971

2.179268e-05

2.179268e-05

2.179268e-05

2.179268e-05

2.179268e-05

3.955924e-05

3.955924e-05

3.955924e-05

3.955924e-05

3.955924e-05

0.0586528872100004

0.000926280129999999

9.78515e-06

9.78515e-06

9.78515e-06

0.000668187

0.000668187

0.000668187

2.444809e-05

2.444809e-05

2.444809e-05

0.00022385989

0.00022385989

0.00022385989

0.0308658289499993

0.00166620118999999

0.00166620118999999

0.00166620118999999

0.0001941306

1.22314e-06

1.22314e-06

1.886981e-05

1.886981e-05

0.00012878132

0.00012878132

4.525633e-05

4.525633e-05

0.000331106789999999

3.772277e-05

3.772277e-05

1.098505e-05

1.098505e-05

0.00028239897

0.00028239897

0.0124470205899995

0.000306516729999999

0.000306516729999999

0.000906621799999999

0.000906621799999999

2.324172e-05

2.324172e-05

0.00331827789000004

0.00331827789000004

7.426539e-05

7.426539e-05

1.22314e-06

1.22314e-06

0.00781687392000023

0.00781687392000023

0.00580285615000003

1.22492e-06

1.22492e-06

0.00244160866000002

0.00244160866000002

2.851236e-05

2.851236e-05

0.0025718251

0.0025718251

0.00033883656

0.00033883656

0.00017858439

0.00017858439

0.00015195847

0.00015195847

9.030569e-05

9.030569e-05

0.0104245136299999

0.00726317150000002

0.00026923021

7.51737e-06

7.2256e-06

0.000555824669999999

0.00575734368000002

0.00066602997

0.00023065477

2.3857e-06

0.00022826907

0.00293068736000003

0.00293068736000003

3.439165e-05

1.22492e-06

1.22492e-06

1.22492e-06

9.46092e-06

9.46092e-06

9.46092e-06

1.724194e-05

1.724194e-05

1.724194e-05

6.46387e-06

6.46387e-06

6.46387e-06

0.0216569325399998

2.43854e-06

2.43854e-06

2.43854e-06

4.015408e-05

4.015408e-05

4.015408e-05

0.0180285673699997

0.0180285673699997

0.0180285673699997

1.135644e-05

1.135644e-05

1.135644e-05

4.8017e-06

4.8017e-06

4.8017e-06

0.00025267133

0.00025267133

0.00025267133

1.22492e-06

1.22492e-06

1.22492e-06

0.00148372219

0.00148372219

0.00148372219

0.000481821

0.000481821

0.000481821

1.39456e-06

1.39456e-06

1.39456e-06

0.00021749314

0.00021749314

0.00021749314

0.00018693025

0.00018693025

0.00018693025

7.18473e-06

7.18473e-06

7.18473e-06

1.22314e-06

1.22314e-06

1.22314e-06

8.584302e-05

8.584302e-05

8.584302e-05

2.44629e-06

2.44629e-06

2.44629e-06

3.677146e-05

3.677146e-05

3.677146e-05

0.00071155218

0.00071155218

0.00071155218

2.151318e-05

2.151318e-05

2.151318e-05

8.57441e-06

8.57441e-06

8.57441e-06

6.189911e-05

6.189911e-05

6.189911e-05

2.44983e-06

2.44983e-06

2.44983e-06

4.89967e-06

4.89967e-06

4.89967e-06

3.66943e-06

3.66943e-06

3.66943e-06

3.66943e-06

0.00515477624000006

1.22492e-06

1.22492e-06

1.22492e-06

7.84923e-06

7.84923e-06

7.84923e-06

0.00514570209000006

1.223144e-05

1.223144e-05

0.00510299573000006

5.06366e-06

0.00403148755000005

0.00049413725

0.00024084725

0.00033146002

3.047492e-05

3.047492e-05

6.11571e-06

6.11571e-06

6.11571e-06

6.11571e-06

4.89256e-06

4.89256e-06

4.89256e-06

4.89256e-06

6.11571e-06

1.22314e-06

1.22314e-06

1.22314e-06

1.22314e-06

4.89257e-06

4.89257e-06

4.89257e-06

4.89257e-06

0.00696710845000011

2.095709e-05

2.095709e-05

2.095709e-05

2.095709e-05

0.00540339500000008

0.00014048122

0.00014048122

0.00014048122

0.00021598935

0.00021598935

0.00021598935

0.0013560914

0.0013560914

0.0013560914

0.00054471919

0.00054471919

0.00054471919

0.00314611384000003

0.00314611384000003

0.00314611384000003

0.00154275635999999

0.00017007664

0.00017007664

0.00017007664

0.00135125554999999

3.86049e-05

3.86049e-05

1.22314e-06

1.22314e-06

0.00131142750999999

0.00131142750999999

2.142417e-05

2.41599e-06

2.41599e-06

1.900818e-05

1.900818e-05

0.00039355759

0.00039355759

4.24056e-06

4.24056e-06

4.24056e-06

4.24056e-06

1.829292e-05

1.22314e-06

1.22314e-06

1.22314e-06

1.706978e-05

1.706978e-05

1.706978e-05

9.240073e-05

4.18367e-06

4.18367e-06

4.18367e-06

1.642486e-05

1.642486e-05

1.642486e-05

7.17922e-05

7.17922e-05

7.17922e-05

2.11398e-05

6.11926e-06

6.11926e-06

6.11926e-06

4.01226e-06

4.01226e-06

4.01226e-06

1.100828e-05

1.100828e-05

1.100828e-05

0.00025748358

0.00011379503

0.00011379503

0.00011379503

2.489554e-05

2.489554e-05

2.489554e-05

0.00011879301

0.00011879301

0.00011879301

0.0010832599

0.0010832599

0.0010832599

0.00015191698

1.314454e-05

1.314454e-05

0.00013877244

0.00013877244

0.000931342919999999

0.000931342919999999

3.883342e-05

0.000643764359999999

7.395357e-05

2.932705e-05

2.327342e-05

0.0001221911

0.185810274530003

0.0171219434599991

0.0171219434599991

6.536163e-05

6.536163e-05

6.536163e-05

0.00018436219

1.21927e-06

1.21927e-06

4.01404e-06

4.01404e-06

1.21927e-06

1.21927e-06

0.00017790961

0.00017790961

0.00021785386

3.045317e-05

1.39456e-06

2.905861e-05

0.00018740069

3.826905e-05

0.00014546254

3.6691e-06

0.00206887804

0.000890067459999999

0.00018985814

0.00041503874

0.00028517058

0.000891972929999997

0.000761069429999998

9.092131e-05

3.998219e-05

0.0002439733

0.0002439733

4.286435e-05

2.452981e-05

1.833454e-05

0.00881890668000005

0.000632739609999999

0.000632739609999999

7.138021e-05

7.138021e-05

1.086211e-05

1.086211e-05

0.00025205188

0.00025205188

9.01566e-06

9.01566e-06

0.0014099165

0.0014099165

0.00010545893

0.00010545893

0.00597854675000005

0.00597854675000005

5.39475e-05

5.39475e-05

0.00013456158

0.00013456158

5.06399e-06

5.06399e-06

0.00015536196

0.00015536196

0.00452426807

0.00452426807

0.00452426807

0.00122129696

0.00122129696

1.592394e-05

5.4086e-06

2.44628e-06

3.184765e-05

1.901314e-05

0.0002152343

0.000931423049999998

2.101603e-05

2.101603e-05

2.101603e-05

0.0900802768400157

0.00018688569

0.00018688569

0.00018476541

3.67298e-06

4.24056e-06

0.00017685187

2.12028e-06

2.12028e-06

0.000962930309999997

0.000962930309999997

4.786356e-05

4.786356e-05

0.00018973136

0.00018973136

0.000725335389999998

8.56201e-06

0.000581948659999999

9.541982e-05

1.98848e-05

1.95201e-05

0.00167726072

0.00167726072

0.00167726072

0.00167726072

0.0219987478299989

0.0219987478299989

1.647194e-05

1.279719e-05

3.67475e-06

0.000665065259999999

0.00033000405

0.000305147009999999

2.99142e-05

0.0001345944

2.28506e-06

0.00013230934

0.000486191159999999

0.00021429378

0.00027189738

3.67475e-06

3.67475e-06

1.885068e-05

2.3857e-06

1.646498e-05

0.00706869858000007

0.00012133692

0.00692140654000007

6.32895e-06

1.226299e-05

3.72049e-06

3.64269e-06

0.0122610991899998

0.0122610991899998

0.000355209229999999

0.00031249391

3.6418e-05

1.22492e-06

1.22314e-06

3.84926e-06

8.868324e-05

5.23541e-06

8.344783e-05

2.44629e-06

2.44629e-06

1.22492e-06

1.22492e-06

0.00089653819

6.132586e-05

0.00083521233

0.0105261044999998

0.000479465299999999

0.00011476109

2.37597e-05

9.100139e-05

6.088608e-05

6.088608e-05

2.43854e-06

2.43854e-06

0.00015066311

4.712943e-05

0.00010353368

1.22492e-06

1.22492e-06

0.00014949156

0.00011270549

4.18367e-06

3.26024e-05

0.0100380338199999

0.000853215339999998

0.00015305352

0.00024308408

0.00045707774

0.00133969267

0.00127749118

6.220149e-05

0.00403290164000003

0.00041387377

7.31562e-06

0.00021695318

4.83553e-06

3.666692e-05

3.770531e-05

1.81529e-05

3.57856e-06

2.17448e-05

0.00327207505000004

1.22492e-06

1.22492e-06

0.00239456256000003

0.00239456256000003

0.000875395739999998

2.44983e-06

0.000872945909999998

0.00054104095

3.67475e-06

0.000395125849999999

0.00013857092

3.66943e-06

8.60538e-06

8.60538e-06

8.60538e-06

0.00261559219000002

0.00261559219000002

2.010394e-05

1.064222e-05

8.06716e-06

1.39456e-06

4.204557e-05

2.362246e-05

1.842311e-05

0.00152348385

0.000402193479999998

0.00112129037

6.375685e-05

9.28267e-06

5.447418e-05

0.000966201979999997

0.000966201979999997

0.024048422169999

3.915633e-05

3.915633e-05

1.22492e-06

3.180861e-05

6.1228e-06

0.00045235929

3.80668e-06

3.80668e-06

9.980137e-05

4.697819e-05

5.282318e-05

0.00034875124

2.25299e-06

0.00034649825

0.00219999804000002

4.73489e-06

4.73489e-06

0.0016802965

0.0016802965

0.000514966649999998

0.000514966649999998

0.00111981264

0.00111981264

0.00111981264

0.000410855659999999

1.181819e-05

1.19285e-06

1.062534e-05

0.000311510589999999

0.000311510589999999

8.752688e-05

2.099588e-05

6.6531e-05

0.0196653281099996

6.12458e-06

6.12458e-06

0.00378407281000003

1.19285e-06

0.000832003959999998

0.002950876

0.00548223133000013

0.00253088184000003

0.00015615964

0.00279518985000001

0.0078291911800001

1.22492e-06

4.86406e-06

0.00105410216

3.86464e-05

8.72955e-06

0.00209450948999999

0.0046271146

0.00256370821000002

0.00256370821000002

0.0001609121

1.39456e-06

1.39456e-06

0.00015951754

0.00015271616

6.80138e-06

0.00933845026999995

0.00933845026999995

0.000512284549999999

0.00020411614

3.78026e-06

0.00030438815

0.000623525389999998

0.00032623113

0.000297294259999999

3.486809e-05

3.75472e-06

3.111337e-05

0.00024300933

0.00023814172

4.86761e-06

0.00159718464

0.00159718464

0.00026961772

2.915795e-05

0.00024045977

5.564909e-05

4.518298e-05

7.93453e-06

2.53158e-06

2.293176e-05

1.06014e-06

2.187162e-05

0.00426498652

1.06014e-06

3.574967e-05

0.000329653869999999

0.00342443895000004

5.13728e-05

2.53158e-06

3.561629e-05

0.00038456322

0.00159112591

0.00031367791

0.000411000849999999

0.000866447149999998

0.00012326727

8.56959e-06

0.00011190856

2.78912e-06

0.00120513706999999

5.356906e-05

5.250892e-05

2.61383e-06

4.777481e-05

2.12028e-06

1.06014e-06

1.06014e-06

0.00032580632

7.107884e-05

3.800604e-05

3.30728e-05

0.00017058871

6.859538e-05

6.279775e-05

3.919558e-05

7.925039e-05

6.29423e-06

7.295616e-05

4.88838e-06

4.88838e-06

0.000755947819999999

0.000755947819999999

1.22314e-06

2.44983e-06

0.000752274849999999

6.981387e-05

6.981387e-05

2.44983e-06

6.736404e-05

0.000436291539999999

2.78912e-06

2.78912e-06

2.78912e-06

4.060713e-05

4.060713e-05

4.060713e-05

0.00019392562

5.9108e-06

2.43854e-06

2.25299e-06

1.21927e-06

4.314203e-05

1.482945e-05

2.831258e-05

0.00014487279

0.00014487279

0.00019896967

1.834084e-05

1.834084e-05

1.336368e-05

1.336368e-05

0.00016726515

1.684183e-05

0.00015042332

0.0170844545500002

4.764606e-05

4.519623e-05

2.49071e-06

4.270552e-05

2.44983e-06

2.44983e-06

0.0118251320400001

1.06014e-06

1.06014e-06

0.00173788601999999

0.00173788601999999

0.0013461158

0.00015801269

0.00118810311

0.000542521289999999

0.00041454635

0.00012797494

0.00819754878999999

1.10207e-05

0.00406015268

9.79933e-06

5.07635e-06

0.00299301121000006

0.00111604223

2.44629e-06

0.00209556891000001

3.4323e-05

3.4323e-05

9.177716e-05

1.22314e-06

9.055402e-05

1.84804e-05

1.84804e-05

0.00019900614

2.217895e-05

1.21927e-06

0.00017560792

4.350433e-05

6.46387e-06

2.44629e-06

3.459417e-05

2.78912e-06

2.78912e-06

2.12028e-06

2.12028e-06

0.00170356847999999

0.00170356847999999

0.00029616041

0.00029616041

0.00029616041

0.00027218517

0.00027218517

0.00013200231

9.24111099999999e-05

4.777175e-05

0.00034426438

1.22492e-06

1.22492e-06

2.43854e-06

2.43854e-06

7.42098e-06

7.42098e-06

0.00033317994

3.67475e-06

0.00032950519

0.000583791109999999

3.740334e-05

3.740334e-05

0.00015008189

6.1334e-06

0.00011367643

3.027206e-05

0.00028654608

0.00028654608

6.137052e-05

7.15711e-06

5.421341e-05

2.128616e-05

1.338779e-05

7.89837e-06

2.710312e-05

2.588385e-05

1.21927e-06

2.817306e-05

2.817306e-05

2.572323e-05

2.44983e-06

0.00158773604

0.00123132632

6.12458e-06

0.000722718109999998

1.22314e-06

2.49852e-05

0.00047627529

6.215789e-05

6.215789e-05

1.22492e-06

1.22492e-06

0.00029302691

5.871553e-05

0.00023431138

3.79737e-06

3.79737e-06

3.79737e-06

0.00519772779000004

0.00010138541

0.00010138541

0.00010138541

0.00010138541

1.340641e-05

1.340641e-05

1.340641e-05

1.340641e-05

0.00217515694000001

0.00217515694000001

0.00217515694000001

0.00217515694000001

0.00290777903000003

0.00290777903000003

0.00290777903000003

0.00290777903000003

0.00350145954000004

4.480279e-05

4.480279e-05

4.480279e-05

4.480279e-05

0.00344811799000003

0.00344811799000003

0.00344811799000003

0.00141709392

4.39969e-06

4.700797e-05

0.00018032812

0.00179928829

8.53876e-06

8.53876e-06

8.53876e-06

1.22314e-06

7.31562e-06

0.0224902554799988

0.0224902554799988

0.0224902554799988

0.0224902554799988

0.0172320423199991

0.00275681833000002

2.3857e-06

0.00194018481

0.000558824319999999

0.0444320674399987

0.0010348078

0.0010348078

0.0010348078

0.0010348078

0.0329366209699995

0.000328187959999999

0.000328187959999999

0.000328187959999999

1.941713e-05

3.18042e-06

3.18042e-06

1.623671e-05

1.623671e-05

0.00316354073000002

0.00146793219

0.00146793219

1.012799e-05

1.012799e-05

0.00101647102

0.00101647102

0.00025943223

0.00025943223

2.490574e-05

2.490574e-05

0.00033619053

0.00033619053

4.848103e-05

4.848103e-05

0.00135946346

2.43854e-06

2.43854e-06

0.000478578069999999

0.000478578069999999

0.000878446849999998

0.000878446849999998

0.00882926782999995

0.00882926782999995

0.00882926782999995

5.20511e-06

5.20511e-06

1.22314e-06

3.98197e-06

0.00614298343000006

0.00614298343000006

0.00337846664000007

7.251853e-05

0.00020771459

0.00248428367000001

0.00580870133000005

0.00196580643

0.00196580643

0.0001372114

0.0001372114

0.00330093559000005

0.00330093559000005

1.22314e-06

1.22314e-06

0.00013765714

0.00013765714

0.00026586763

0.00026586763

0.000461338809999999

0.000461338809999999

0.000461338809999999

6.006452e-05

1.22492e-06

1.22492e-06

3.085393e-05

3.085393e-05

2.798567e-05

2.798567e-05

0.0001422712

0.0001422712

0.0001422712

0.00531323743000004

0.00531323743000004

5.56612e-06

0.00370904484000002

3.75472e-06

0.000518619249999999

0.000587500189999996

0.00048630248

2.44983e-06

0.00117611061

0.00116445374

0.00116445374

1.165687e-05

1.165687e-05

0.00012683142

0.00012683142

0.00012683142

0.0104606386700001

0.00659946163000004

3.768208e-05

3.768208e-05

9.37199e-05

9.37199e-05

0.00341605574000004

0.00341605574000004

0.00302909256000002

0.00302909256000002

1.22492e-06

1.22492e-06

3.67475e-06

3.67475e-06

1.801168e-05

1.801168e-05

7.31744e-06

7.31744e-06

7.31744e-06

0.000695766279999999

0.000695766279999999

0.000695766279999999

0.00315809332000003

0.00315809332000003

0.00227955894000002

2.44983e-06

1.053437e-05

0.000780242309999999

8.530787e-05

0.00110856253

0.00110856253

0.00110856253

0.00110856253

0.00110856253

0.00187798145

3.57856e-06

3.57856e-06

3.57856e-06

3.57856e-06

0.00010710901

0.00010710901

0.00010710901

0.00010710901

0.000716012789999999

0.000716012789999999

0.000716012789999999

0.000716012789999999

0.00105128109

0.00105128109

0.00105128109

0.00105128109

1.39456e-06

1.39456e-06

1.39456e-06

1.39456e-06

1.39456e-06

1.39456e-06

0.00010263975

0.00010010817

0.00010010817

0.00010010817

0.00010010817

0.00010010817

2.53158e-06

2.53158e-06

2.53158e-06

2.53158e-06

2.53158e-06

0.0643304983899886

1.381777e-05

1.381777e-05

1.381777e-05

1.381777e-05

1.381777e-05

0.00141235958

1.21927e-06

1.21927e-06

1.21927e-06

1.21927e-06

0.00137871241

0.00137871241

0.00137871241

0.00137871241

4.89258e-06

4.89258e-06

4.89258e-06

4.89258e-06

3.66556e-06

3.66556e-06

3.66556e-06

3.66556e-06

1.098296e-05

1.098296e-05

1.098296e-05

1.098296e-05

1.28868e-05

1.28868e-05

1.28868e-05

1.28868e-05

4.752262e-05

4.752262e-05

4.752262e-05

4.752262e-05

4.752262e-05

0.0339546326999984

0.0339546326999984

0.0339546326999984

5.712e-06

3.18042e-06

1.26579e-06

1.26579e-06

6.588448e-05

6.588448e-05

0.0005685878

1.26579e-06

0.00029036114

7.68524e-06

2.44629e-06

2.78912e-06

0.00026159038

2.44984e-06

2.438547e-05

2.438547e-05

0.00218373493000001

0.00139860046999999

1.623985e-05

0.000768894609999999

0.00776534532000012

0.00776534532000012

0.000528602680000001

1.704018e-05

0.00031410023

0.00018600033

1.146194e-05

0.00331819542000003

5.40295e-06

9.377405e-05

0.00010414307

0.00244270066000003

3.54907e-06

0.000351446789999999

6.668552e-05

1.06014e-06

9.367976e-05

4.599102e-05

5.132228e-05

3.67476e-06

1.624369e-05

3.852166e-05

0.00149361061

2.44983e-06

4.244187e-05

0.00114088796

0.0001574516

2.51574e-05

1.012799e-05

4.892195e-05

6.617201e-05

0.0171593620299991

4.18368e-06

6.186471e-05

1.22314e-06

0.00026043376

2.44983e-06

0.0145941084599998

1.186537e-05

1.087893e-05

4.147745e-05

1.19285e-06

2.44984e-06

0.000615909129999999

9.01196e-06

0.00024290459

1.095125e-05

0.00101506425

3.915098e-05

0.00023424185

0.000488940699999999

0.00035136943

3.911382e-05

9.845745e-05

0.000347023499999999

0.00020146229

3.66555e-06

0.00014189566

5.24776e-06

1.19285e-06

2.78912e-06

1.26579e-06

0.0288757834099982

1.668068e-05

1.249701e-05

1.249701e-05

1.249701e-05

4.18367e-06

4.18367e-06

4.18367e-06

0.00019434007

0.00019434007

3.65781e-06

3.65781e-06

3.66943e-06

2.44629e-06

1.22314e-06

1.288858e-05

1.288858e-05

2.495387e-05

8.19593e-06

1.39456e-06

1.536338e-05

0.00014917038

3.962076e-05

2.44629e-06

0.00010710333

0.0286439473399982

3.711767e-05

3.711767e-05

3.711767e-05

0.00651361935000009

0.00651361935000009

0.00651361935000009

0.00276657491000003

0.00276657491000003

0.00276657491000003

0.0182547903299989

0.0182547903299989

0.0182547903299989

9.46286e-05

9.46286e-05

9.46286e-05

0.000661692519999999

0.000661692519999999

0.000661692519999999

2.53158e-06

2.53158e-06

2.53158e-06

1.588472e-05

1.588472e-05

1.588472e-05

0.00029710766

0.00029710766

0.00029710766

2.081532e-05

2.081532e-05

2.081532e-05

2.081532e-05

1.135112e-05

1.22314e-06

1.22314e-06

1.22314e-06

1.22314e-06

7.33886e-06

7.33886e-06

7.33886e-06

7.33886e-06

2.78912e-06

2.78912e-06

2.78912e-06

2.78912e-06

1.503119e-05

3.66943e-06

3.66943e-06

3.66943e-06

3.66943e-06

1.136176e-05

1.136176e-05

1.136176e-05

1.136176e-05

0.00523596112

0.00146688884

2.53158e-06

2.53158e-06

2.53158e-06

2.53158e-06

8.15054e-06

1.06014e-06

1.06014e-06

1.06014e-06

7.0904e-06

4.60534e-06

1.06014e-06

3.5452e-06

2.48506e-06

2.48506e-06

0.00145620672

8.4729e-06

3.67121e-06

3.67121e-06

3.57855e-06

2.3857e-06

1.19285e-06

1.22314e-06

1.22314e-06

1.22314e-06

1.22314e-06

1.22314e-06

2.316614e-05

2.316614e-05

2.316614e-05

0.000736056999999998

0.00025221126

1.77585e-05

6.34271100000001e-05

0.00017102565

8.62793e-06

8.62793e-06

5.703265e-05

5.703265e-05

0.00041447308

0.00041325381

1.21927e-06

3.71208e-06

3.71208e-06

4.50598e-06

4.50598e-06

1.06014e-06

3.44584e-06

1.17325e-05

1.06014e-06

1.06014e-06

7.02968e-06

7.02968e-06

1.19285e-06

1.19285e-06

2.44983e-06

2.44983e-06

4.737611e-05

4.737611e-05

4.737611e-05

0.00026902602

2.43854e-06

2.43854e-06

0.00017037083

1.06014e-06

7.585022e-05

9.346047e-05

3.5452e-06

3.5452e-06

7.83228e-06

7.83228e-06

5.691648e-05

4.97964e-06

2.023577e-05

3.170107e-05

1.329142e-05

1.21927e-06

1.207215e-05

1.463127e-05

1.21927e-06

1.3412e-05

2.43854e-06

2.43854e-06

2.43854e-06

0.000242474

6.54562e-06

2.53158e-06

4.01404e-06

3.38607e-06

1.06014e-06

2.32593e-06

2.44628e-06

2.44628e-06

1.213614e-05

1.091122e-05

1.22492e-06

0.0002133489

0.0002133489

1.22492e-06

1.22492e-06

3.38607e-06

3.38607e-06

0.00010973439

1.463124e-05

1.463124e-05

9.510315e-05

9.510315e-05

0.00370175291000003

0.00013861577

1.21927e-06

1.21927e-06

1.21927e-06

4.813157e-05

5.95981e-06

5.95981e-06

4.5324e-06

4.5324e-06

1.22492e-06

1.22492e-06

1.22314e-06

1.22314e-06

1.22314e-06

1.22314e-06

3.396816e-05

4.89968e-06

2.906848e-05

6.953337e-05

1.39456e-06

1.39456e-06

6.33819e-06

6.33819e-06

6.180062e-05

6.180062e-05

1.06014e-06

1.06014e-06

1.06014e-06

1.15715e-05

1.15715e-05

1.15715e-05

7.09992e-06

5.83413e-06

4.56834e-06

1.26579e-06

1.26579e-06

1.26579e-06

0.00351288027000003

1.872145e-05

7.14623e-06

2.43854e-06

4.70769e-06

2.12028e-06

2.12028e-06

1.06014e-06

1.06014e-06

2.28328e-06

2.28328e-06

6.11152e-06

2.44984e-06

3.66168e-06

0.00143965105

0.00143965105

0.00143965105

0.00028371772

1.010418e-05

1.010418e-05

2.41212e-06

2.41212e-06

1.72841e-05

1.72841e-05

0.00024669608

1.21927e-06

1.22314e-06

3.67475e-06

0.00022337973

2.3857e-06

4.8676e-06

1.22492e-06

1.21927e-06

7.5017e-06

7.22124e-06

2.44983e-06

4.77141e-06

5.717693e-05

5.717693e-05

1.394558e-05

4.323135e-05

0.000641164829999998

1.955882e-05

1.955882e-05

0.00010156663

8.44177899999999e-05

1.714884e-05

1.39456e-06

1.39456e-06

0.00051152107

5.06316e-06

7.33887e-06

1.19285e-06

4.268997e-05

2.8055e-05

0.000427181219999999

7.12375e-06

7.12375e-06

0.00107244829

0.00025576926

0.00025576926

6.739792e-05

3.041952e-05

3.45927e-05

2.3857e-06

0.00065728144

0.00065728144

7.261897e-05

7.139405e-05

1.22492e-06

3.67475e-06

3.67475e-06

1.570595e-05

1.22492e-06

6.69526e-06

7.78577e-06

5.025687e-05

5.025687e-05

5.025687e-05

5.025687e-05

1.06014e-06

1.06014e-06

1.06014e-06

1.06014e-06

1.06014e-06

6.625923e-05

6.625923e-05

6.625923e-05

7.55387e-06

1.26579e-06

1.22492e-06

5.06316e-06

4.39969e-06

4.39969e-06

4.461915e-05

1.26579e-06

4.107395e-05

2.27941e-06

2.43854e-06

2.43854e-06

7.24798e-06

7.24798e-06

0.0679906726499964

0.000736863339999998

0.000736863339999998

0.000736863339999998

0.000736863339999998

1.26579e-06

0.000735597549999998

9.74402e-06

9.74402e-06

9.74402e-06

9.74402e-06

9.74402e-06

0.00120223189

9.070384e-05

9.070384e-05

9.070384e-05

9.070384e-05

0.000509828389999999

0.000509828389999999

0.000509828389999999

0.000509828389999999

0.00021662812

0.00021662812

0.00021662812

0.00021662812

0.00038507154

0.00038507154

0.00038507154

0.00038507154

0.00789347245000005

1.577814e-05

1.577814e-05

1.577814e-05

1.577814e-05

3.522285e-05

3.522285e-05

3.522285e-05

3.522285e-05

1.22492e-06

1.22492e-06

1.22492e-06

1.22492e-06

0.00385351501000003

0.00385351501000003

0.00385351501000003

0.00385351501000003

0.00259512692000004

0.00259512692000004

0.00259512692000004

0.00259512692000004

3.305968e-05

3.305968e-05

3.305968e-05

3.305968e-05

1.22314e-06

1.22314e-06

1.22314e-06

1.22314e-06

1.803581e-05

1.803581e-05

1.803581e-05

1.803581e-05

2.44983e-06

2.44983e-06

2.44983e-06

2.44983e-06

0.00129763492

0.00129763492

0.00129763492

0.00129763492

3.408551e-05

3.408551e-05

3.408551e-05

3.408551e-05

6.11572e-06

6.11572e-06

6.11572e-06

6.11572e-06

0.00110413908

7.81912e-06

7.81912e-06

7.81912e-06

7.81912e-06

0.0001247873

0.0001247873

0.0001247873

0.0001247873

3.008856e-05

3.008856e-05

3.008856e-05

3.008856e-05

0.000681959129999998

0.000681959129999998

0.000681959129999998

0.000681959129999998

0.00025948497

0.00025948497

0.00025948497

0.00025948497

1.049991e-05

1.049991e-05

1.049991e-05

1.049991e-05

1.049991e-05

0.00160484431

0.00160484431

0.00160484431

7.352664e-05

1.321542e-05

6.12459e-06

5.418663e-05

0.00151373384

0.000328588959999999

0.00118514488

1.758383e-05

1.758383e-05

0.00397933224000003

4.89258e-06

4.89258e-06

4.89258e-06

4.89258e-06

0.00122347296

0.00122347296

0.00122347296

0.00122347296

1.22314e-06

1.22314e-06

1.22314e-06

1.22314e-06

1.22492e-06

1.22492e-06

1.22492e-06

1.22492e-06

0.00274851864000003

0.00274851864000003

0.00274851864000003

0.00274851864000003

0.00130575217

0.00130575217

0.00130575217

0.00124652194

6.46033e-06

4.957899e-05

4.18368e-06

1.396496e-05

0.00012717605

8.562e-06

0.0010310177

5.57823e-06

5.923023e-05

5.923023e-05

1.129054e-05

1.00674e-05

1.00674e-05

1.00674e-05

1.00674e-05

1.22314e-06

1.22314e-06

1.22314e-06

1.22314e-06

0.00362105347000005

0.00360887762000005

0.00309649698000004

0.00309649698000004

0.00309649698000004

3.292811e-05

3.292811e-05

3.292811e-05

0.00047945253

0.00047945253

0.00047945253

1.217585e-05

2.44629e-06

2.44629e-06

2.44629e-06

9.72956e-06

9.72956e-06

9.72956e-06

2.046684e-05

2.046684e-05

2.046684e-05

2.046684e-05

2.046684e-05

0.0105533001499999

1.22492e-06

1.22492e-06

1.22492e-06

1.22492e-06

0.00159012762

0.00159012762

0.00159012762

0.00159012762

0.00662649988000007

0.00662649988000007

0.00662649988000007

0.00662649988000007

2.43854e-06

2.43854e-06

2.43854e-06

2.43854e-06

0.00233300919000002

0.00233300919000002

0.00233300919000002

0.00233300919000002

0.000459538549999999

1.21927e-06

1.21927e-06

1.21927e-06

1.21927e-06

0.000407483179999999

0.000407483179999999

0.000407483179999999

0.000407483179999999

3.387968e-05

3.387968e-05

3.387968e-05

3.387968e-05

1.695642e-05

1.695642e-05

1.695642e-05

1.695642e-05

0.0135697790999997

0.00147687184

1.483927e-05

1.39456e-06

1.39456e-06

3.66168e-06

3.66168e-06

9.78303e-06

6.1157e-06

3.66733e-06

5.48513e-05

5.48513e-05

5.48513e-05

1.22314e-06

1.22314e-06

1.22314e-06

0.000466063789999999

0.000466063789999999

0.000466063789999999

0.000939894339999996

0.000939894339999996

0.000429152829999999

0.00011743168

1.26579e-06

7.51028e-06

0.000384533759999999

0.00108248671

0.00108248671

0.00108248671

3.129147e-05

0.0007000574

0.000351137839999999

0.0109274864999998

0.00018793072

0.00018793072

0.00017691952

1.10112e-05

0.00168822381

0.00168822381

0.00168822381

0.00905133197000004

0.00315245951000005

0.00315245951000005

4.830593e-05

4.830593e-05

0.00028549424

0.00028549424

3.79737e-06

3.79737e-06

0.00523605105

0.00523605105

0.000325223869999999

0.000325223869999999

8.293405e-05

8.293405e-05

8.293405e-05

8.293405e-05

0.00303727248000003

3.861201e-05

3.861201e-05

3.861201e-05

3.861201e-05

0.00261008008000003

2.712392e-05

2.712392e-05

2.712392e-05

0.000449720779999999

0.000449720779999999

0.000449720779999999

0.00204318087000001

0.00204318087000001

0.00204318087000001

9.005451e-05

9.005451e-05

9.005451e-05

0.00038858039

0.00038858039

0.00038858039

0.00038858039

0.00226610527000002

0.00226610527000002

0.00226610527000002

0.00226610527000002

0.00226610527000002

0.0166049868399997

0.00114631882

0.00016000251

0.00016000251

0.00016000251

4.87709e-06

4.87709e-06

4.87709e-06

1.616537e-05

1.616537e-05

1.616537e-05

0.000963879289999997

0.000963879289999997

0.000963879289999997

1.39456e-06

1.39456e-06

1.39456e-06

0.000594783849999999

0.000594783849999999

1.680115e-05

2.27941e-06

3.65781e-06

8.47823e-06

2.3857e-06

0.000572518999999999

0.000572518999999999

5.4637e-06

5.4637e-06

0.0147034192499999

0.00021501264

0.00021501264

0.00021501264

0.00434969502000001

0.00434969502000001

0.00434969502000001

4.205779e-05

4.205779e-05

4.205779e-05

0.00110123944

0.00110123944

0.00110123944

0.00114457211

0.00114457211

0.00114457211

0.00785084225000003

0.00785084225000003

0.00785084225000003

0.00016046492

0.00016046492

0.00016046492

0.00016046492

2.49886e-05

2.6177e-06

2.6177e-06

2.6177e-06

2.6177e-06

2.6177e-06

2.23709e-05

2.23709e-05

2.23709e-05

2.23709e-05

2.23709e-05

0.0001999203

5.70764e-05

5.70764e-05

5.70764e-05

5.70764e-05

5.70764e-05

3.935916e-05

3.935916e-05

3.935916e-05

3.935916e-05

3.935916e-05

6.376204e-05

6.376204e-05

6.376204e-05

6.376204e-05

6.376204e-05

3.97227e-05

3.97227e-05

3.97227e-05

3.97227e-05

3.97227e-05

9.79224e-06

9.79224e-06

9.79224e-06

9.79224e-06

9.79224e-06

9.79224e-06

0.000896209619999999

0.000893724559999999

0.000893724559999999

0.000875941989999999

0.00040356276

0.00040356276

0.00030069571

0.00030069571

0.00017168352

0.00017168352

2.44241e-06

2.44241e-06

2.44241e-06

1.534016e-05

1.534016e-05

1.534016e-05

2.48506e-06

2.48506e-06

2.48506e-06

2.48506e-06

2.48506e-06

1.435702e-05

5.57824e-06

5.57824e-06

5.57824e-06

5.57824e-06

5.57824e-06

8.77878e-06

8.77878e-06

8.77878e-06

8.77878e-06

8.77878e-06

9.263815e-05

3.417826e-05

3.417826e-05

3.417826e-05

3.417826e-05

3.417826e-05

5.112733e-05

5.112733e-05

5.112733e-05

5.112733e-05

5.112733e-05

7.33256e-06

7.33256e-06

7.33256e-06

7.33256e-06

7.33256e-06

0.00044540561

0.00044540561

0.00044540561

0.00044540561

0.00044540561

0.00044540561

0.00044540561
