# Supplementary material for: Succession of bacterial communities on carrion is independent of vertebrate scavengers
Source: PeerJ. 2020 Jun 10;8:e9307. doi: 10.7717/peerj.9307 (PMC7293191; doi:10.7717/peerj.9307)
Supplement: Dataset S1 — Interactive Krona plots, and the full count table showing the distribution of each OTU among all samples. [file peerj-08-9307-s001.zip › DS2_COW.bloat_stage_krona.html]

Javascript must be enabled to view this page.

members
magnitude
magnitudeUnassigned

COW.week\_2\_krona

0.999999976348451

0.999403825008451

8.09361399999999e-05

2.94842e-06

2.94842e-06

2.94842e-06

2.94842e-06

2.94842e-06

1.031947e-05

1.031947e-05

1.031947e-05

1.031947e-05

1.031947e-05

6.471983e-05

6.471983e-05

6.471983e-05

6.471983e-05

6.471983e-05

1.47421e-06

1.47421e-06

1.47421e-06

1.47421e-06

1.47421e-06

1.47421e-06

1.47421e-06

1.47421e-06

1.47421e-06

1.47421e-06

0.0181458271000014

0.00535765905999982

0.00535765905999982

5.033021e-05

5.033021e-05

5.033021e-05

6.64401e-06

6.64401e-06

6.64401e-06

0.000580858570000001

1.326792e-05

1.326792e-05

0.00018575023

0.00018575023

0.00038184042

0.00038184042

1.47421e-06

1.47421e-06

1.47421e-06

0.000415526949999999

0.000415526949999999

0.000415526949999999

8.84525e-06

2.94842e-06

2.94842e-06

5.89683e-06

5.89683e-06

0.000744801520000002

1.351019e-05

7.82656e-06

5.68363e-06

0.00021069643

0.00019153167

1.326792e-05

5.89684e-06

3.538108e-05

2.211316e-05

1.326792e-05

0.00034081557

0.00034081557

4.51373e-06

1.56531e-06

2.94842e-06

0.00013988452

0.00012283368

1.89454e-06

1.51563e-05

7.37105e-06

7.37105e-06

7.37105e-06

0.00354180729000002

1.010626e-05

1.010626e-05

0.00130082321000001

0.00026349412

1.47421e-06

1.031947e-05

0.00039848432

0.000627051090000003

0.00010761722

8.84525e-06

9.877197e-05

0.000822707050000002

1.91647e-05

0.000748996580000002

2.94842e-06

1.47421e-06

5.012314e-05

4.42263e-06

4.42263e-06

0.000344962179999999

7.37104e-06

8.84525e-06

1.47421e-06

0.000317259459999999

1.001222e-05

0.000904093600000003

0.000904093600000003

1.843768e-05

1.843768e-05

1.916475e-05

1.916475e-05

9.47271e-06

9.47271e-06

0.00167580232

0.00165858391

1.705092e-05

1.515638e-05

1.515638e-05

1.89454e-06

1.89454e-06

7.24894e-06

5.68363e-06

5.68363e-06

1.56531e-06

1.56531e-06

1.3288e-06

1.3288e-06

1.3288e-06

1.13673e-05

1.13673e-05

1.13673e-05

0.00013164189

6.387284e-05

2.79049e-05

3.596794e-05

7.97282e-06

7.97282e-06

5.979623e-05

9.30162e-06

5.049461e-05

0.00136847592

0.00134111666

0.00131022877

2.619195e-05

4.69594e-06

2.2939e-06

1.14695e-06

1.14695e-06

1.14695e-06

1.14695e-06

2.391841e-05

1.993201e-05

2.6576e-06

1.3288e-06

0.00010050178

0.00010050178

3.600223e-05

1.06002e-05

5.389935e-05

4.69594e-06

4.69594e-06

4.69594e-06

1.627242e-05

2.65761e-06

2.65761e-06

7.82657e-06

7.82657e-06

2.65761e-06

2.65761e-06

3.13063e-06

3.13063e-06

1.721841e-05

1.721841e-05

1.721841e-05

1.721841e-05

0.00242412456999999

0.00242412456999999

3.538103e-05

3.538103e-05

3.538103e-05

0.00235188826999999

0.00010394767

0.00010394767

0.00028134507

1.095718e-05

0.00026882258

1.56531e-06

0.000984577690000001

1.878373e-05

0.000909742260000002

5.60517e-05

6.033767e-05

1.095718e-05

4.938049e-05

0.0005011089

1.443517e-05

0.00048667373

3.78908e-06

3.78908e-06

0.00027450827

6.85702e-06

2.94842e-05

0.00023374442

4.42263e-06

7.576209e-05

7.576209e-05

1.962023e-05

1.962023e-05

3.527596e-05

3.9864e-06

3.128956e-05

7.82656e-06

6.26125e-06

1.56531e-06

3.78908e-06

3.78908e-06

3.685527e-05

4.42263e-06

4.42263e-06

3.243264e-05

2.063893e-05

1.47421e-06

1.03195e-05

0.00815291312999968

4.93406e-06

1.89454e-06

1.89454e-06

1.89454e-06

1.56531e-06

1.56531e-06

1.56531e-06

1.47421e-06

1.47421e-06

1.47421e-06

4.127788e-05

4.127788e-05

4.127788e-05

4.127788e-05

0.00030419583

0.00030419583

0.00010909148

3.243265e-05

7.665883e-05

2.262455e-05

2.262455e-05

0.00016805717

7.45279e-06

0.0001209732

3.963118e-05

4.42263e-06

4.42263e-06

0.0078025053599997

0.000647225620000001

0.00051847243

0.000451108039999999

1.851045e-05

4.885394e-05

5.615873e-05

5.615873e-05

7.259446e-05

6.26125e-06

6.633321e-05

0.00635441618999978

8.93636e-06

1.47421e-06

7.46215e-06

0.00199699437000001

0.00199699437000001

0.00104969507

2.80301e-06

0.000880302570000003

0.00010614305

1.14695e-06

1.023027e-05

2.94842e-06

3.832942e-05

4.84296e-06

2.94842e-06

1.179366e-05

1.179366e-05

9.133267e-05

1.3288e-06

9.000387e-05

0.00014018194

1.47421e-06

2.51125e-05

0.00011064681

2.94842e-06

0.00016831657

7.37105e-06

9.44704e-06

5.705797e-05

1.47421e-06

9.29663e-05

5.89684e-06

1.47421e-06

4.42263e-06

0.00149956529000001

0.0003680485

0.00010070261

0.00103081418

0.00010909148

5.896835e-05

3.095841e-05

1.916472e-05

0.00101968154

8.66875e-05

5.89684e-06

2.94842e-06

0.00023734771

0.000290420449999999

0.00039638062

0.00010761727

8.255572e-05

5.89684e-06

1.916471e-05

1.769051e-05

1.47421e-06

1.62163e-05

0.00012762262

2.948417e-05

8.634477e-05

5.89684e-06

5.89684e-06

0.00017985355

0.00010909155

0.00010909155

4.275203e-05

4.275203e-05

2.800997e-05

2.800997e-05

0.00016249404

0.00014642314

2.94842e-06

0.00014347472

1.3288e-06

1.3288e-06

2.94842e-06

1.47421e-06

1.47421e-06

1.179368e-05

1.179368e-05

0.000458515959999998

0.000458515959999998

0.000458515959999998

0.000535328020000001

0.000535328020000001

0.000535328020000001

0.000535328020000001

0.000535328020000001

0.00313743259000003

1.47421e-06

1.47421e-06

1.47421e-06

1.47421e-06

1.47421e-06

0.000800496060000002

0.000800496060000002

0.000800496060000002

0.000800496060000002

0.000800496060000002

6.339098e-05

6.339098e-05

6.339098e-05

6.339098e-05

6.339098e-05

0.00017543101

0.00017543101

0.00017543101

0.00017543101

0.00017543101

0.00166618711000001

0.00166618711000001

0.00166618711000001

0.00166618711000001

0.00166618711000001

0.000389175349999999

0.000389175349999999

0.00020476969

0.00020476969

0.00020476969

6.015178e-05

6.015178e-05

6.015178e-05

0.00012425388

0.00012425388

0.00012425388

4.127787e-05

4.127787e-05

4.127787e-05

4.127787e-05

4.127787e-05

1.89454e-06

1.89454e-06

1.89454e-06

1.89454e-06

1.89454e-06

1.89454e-06

4.983231e-05

2.211314e-05

2.211314e-05

2.211314e-05

2.211314e-05

2.211314e-05

2.771917e-05

2.771917e-05

2.771917e-05

2.771917e-05

2.771917e-05

4.42263e-06

4.42263e-06

4.42263e-06

4.42263e-06

1.47421e-06

1.47421e-06

2.94842e-06

2.94842e-06

0.0563637310999938

3.361604e-05

3.361604e-05

1.916475e-05

1.916475e-05

1.916475e-05

8.55445e-06

8.55445e-06

8.55445e-06

5.89684e-06

5.89684e-06

5.89684e-06

0.0512490269499945

0.0102634422499996

0.0102634422499996

3.243256e-05

3.243256e-05

1.47421e-06

1.47421e-06

0.000255038320000001

0.000255038320000001

0.00997449715999962

0.00893518038999969

2.211314e-05

0.00101720363

0.0312987157200028

0.0312987157200028

0.00026208188

0.00026208188

2.94842e-06

2.94842e-06

7.37105e-06

7.37105e-06

1.03469e-05

1.03469e-05

0.00292377700000002

0.00292377700000002

0.00048501443

1.621631e-05

0.00046879812

4.42263e-06

4.42263e-06

0.00409830117999996

1.47421e-06

0.00307225162000001

0.00102457535

0.000315480979999999

2.94842e-06

0.000296316239999999

1.621632e-05

2.94842e-06

2.94842e-06

0.00017100783

0.00016805941

2.94842e-06

1.780594e-05

1.780594e-05

0.0147497367699994

0.00011646259

0.00156997843000001

2.800997e-05

0.000755635510000002

4.42263e-06

0.000822609020000001

0.0114526186199996

2.94842e-06

2.94842e-06

0.00824452386999979

4.42263e-06

7.37105e-06

0.00705851193999995

0.00116832142000001

5.89683e-06

0.000685507250000002

0.000685507250000002

0.000685507250000002

7.813307e-05

0.000602951550000001

4.42263e-06

0.00536169845999984

0.00536169845999984

0.00536169845999984

1.769052e-05

0.00534400793999985

0.000896318620000002

2.94842e-06

2.94842e-06

2.94842e-06

1.179368e-05

1.179368e-05

1.179368e-05

3.538104e-05

3.538104e-05

3.538104e-05

0.000846195480000002

0.000846195480000002

0.000846195480000002

0.00274334465000001

0.00274334465000001

0.00274334465000001

0.00274334465000001

0.000280099839999999

1.326788e-05

1.326788e-05

1.326788e-05

1.326788e-05

1.47421e-06

1.47421e-06

1.47421e-06

1.47421e-06

2.801001e-05

2.801001e-05

2.801001e-05

2.801001e-05

0.00023734774

0.00023734774

0.00023734774

0.00023734774

1.76905e-05

1.76905e-05

1.76905e-05

1.76905e-05

1.76905e-05

0.00478329776999986

4.42263e-06

4.42263e-06

4.42263e-06

4.42263e-06

0.00036684669

0.00036684669

0.00036684669

0.00036684669

1.56531e-06

1.56531e-06

1.56531e-06

1.56531e-06

1.03195e-05

1.03195e-05

1.03195e-05

1.03195e-05

4.42263e-06

4.42263e-06

4.42263e-06

4.42263e-06

4.426754e-05

4.426754e-05

4.426754e-05

4.426754e-05

4.42263e-06

4.42263e-06

4.42263e-06

4.42263e-06

5.89684e-06

5.89684e-06

5.89684e-06

5.89684e-06

0.0043022027699999

0.0043022027699999

0.0043022027699999

0.0043022027699999

9.44703e-06

9.44703e-06

9.44703e-06

9.44703e-06

2.94842e-05

2.94842e-05

2.94842e-05

2.94842e-05

1.001116e-05

1.001116e-05

1.001116e-05

4.69594e-06

4.69594e-06

4.69594e-06

5.31522e-06

5.31522e-06

5.31522e-06

0.00538779447999982

0.00463920742999987

4.27521e-05

2.801001e-05

2.801001e-05

2.801001e-05

1.474209e-05

1.474209e-05

1.474209e-05

0.00227542115000001

0.00015653372

0.00015653372

0.00015653372

4.42263e-06

4.42263e-06

4.42263e-06

1.031946e-05

4.42263e-06

4.42263e-06

5.89683e-06

5.89683e-06

0.00014175859

1.916475e-05

1.916475e-05

1.710889e-05

1.710889e-05

1.97665e-05

1.97665e-05

2.94842e-06

2.94842e-06

8.277003e-05

8.277003e-05

0.00043477476

5.89684e-06

5.89684e-06

0.00016954695

0.00016954695

8.84525e-06

8.84525e-06

0.00025048572

0.00025048572

0.00152761199000001

0.000841043010000001

0.00017248199

1.90193e-05

0.00050633677

0.00014320495

2.94842e-06

1.47421e-06

1.47421e-06

0.000683620560000001

0.000683620560000001

0.00185932943000001

7.37105e-06

7.37105e-06

7.37105e-06

0.00158808203000001

0.00158808203000001

0.00158808203000001

2.23584e-05

2.23584e-05

2.23584e-05

0.00017468191

0.00017468191

0.00017468191

2.653571e-05

2.653571e-05

2.653571e-05

3.602311e-05

3.602311e-05

3.602311e-05

1.3288e-06

1.3288e-06

1.3288e-06

2.94842e-06

2.94842e-06

2.94842e-06

0.000443850729999999

0.000443850729999999

0.000443850729999999

0.000339181869999999

9.43494e-05

2.94842e-06

7.37104e-06

2.94842e-06

2.94842e-06

2.94842e-06

2.94842e-06

1.49056e-05

1.49056e-05

1.49056e-05

1.49056e-05

0.000748587050000002

2.94842e-06

2.94842e-06

2.94842e-06

2.94842e-06

0.000647912370000002

8.85855e-05

8.85855e-05

8.85855e-05

4.570052e-05

4.570052e-05

4.570052e-05

2.933878e-05

2.933878e-05

2.933878e-05

0.000484287569999999

0.000484287569999999

0.000484287569999999

9.772626e-05

9.182942e-05

9.182942e-05

9.182942e-05

5.89684e-06

1.47421e-06

1.47421e-06

4.42263e-06

4.42263e-06

2.504504e-05

2.504504e-05

2.504504e-05

2.504504e-05

2.504504e-05

2.504504e-05

4.570053e-05

4.570053e-05

2.948422e-05

1.031947e-05

1.031947e-05

1.031947e-05

1.916475e-05

1.916475e-05

1.916475e-05

1.179368e-05

2.94842e-06

2.94842e-06

2.94842e-06

4.42263e-06

4.42263e-06

4.42263e-06

4.42263e-06

4.42263e-06

4.42263e-06

4.42263e-06

4.42263e-06

4.42263e-06

4.42263e-06

0.00522957732999976

0.00522957732999976

0.00522957732999976

0.00522957732999976

0.00522957732999976

0.00522957732999976

0.00619826880999975

0.00619826880999975

1.832308e-05

1.684887e-05

1.684887e-05

1.684887e-05

1.47421e-06

1.47421e-06

1.47421e-06

3.095843e-05

2.358738e-05

2.358738e-05

2.358738e-05

2.94842e-06

2.94842e-06

2.94842e-06

1.47421e-06

1.47421e-06

1.47421e-06

2.94842e-06

2.94842e-06

2.94842e-06

8.99267799999999e-05

8.99267799999999e-05

8.99267799999999e-05

8.99267799999999e-05

0.000370026709999999

8.10816499999999e-05

8.10816499999999e-05

8.10816499999999e-05

0.00024471875

0.00024471875

0.00024471875

3.980368e-05

3.980368e-05

3.980368e-05

4.42263e-06

4.42263e-06

4.42263e-06

0.00410356997999998

1.47421e-06

1.47421e-06

1.47421e-06

0.00253351170000001

0.00253351170000001

0.00253351170000001

4.275206e-05

4.275206e-05

4.275206e-05

0.00107972816

0.00107972816

0.00107972816

0.000446103849999999

0.000446103849999999

0.000446103849999999

0.00158546383000001

0.00158546383000001

0.000686283120000002

0.000686283120000002

0.000423769439999999

0.000305832699999999

3.685526e-05

5.89683e-06

7.518465e-05

0.000475411269999999

2.94842e-06

7.669913e-05

0.00015122236

2.93065e-05

0.00017690547

3.832939e-05

6.486523e-05

6.486523e-05

6.486523e-05

6.486523e-05

6.486523e-05

5.749418e-05

2.94842e-06

4.42263e-06

2.94842e-06

2.94842e-06

2.94842e-06

2.94842e-06

2.94842e-06

2.94842e-06

0.034930939909995

0.00282127081000002

0.00282127081000002

0.00178844468000001

0.00022571759

0.00022571759

5.89684e-06

5.89684e-06

8.845253e-05

8.845253e-05

0.00050534688

0.00050534688

0.000933546650000003

0.000933546650000003

1.47421e-06

1.47421e-06

4.42263e-06

4.42263e-06

2.358735e-05

2.358735e-05

7.37105e-06

5.89684e-06

5.89684e-06

1.47421e-06

1.47421e-06

0.000486639039999999

0.000486639039999999

0.000486639039999999

6.928786e-05

6.928786e-05

8.84526e-06

6.04426e-05

2.6576e-06

2.6576e-06

2.6576e-06

0.00024113432

0.00024113432

2.043922e-05

0.0002206951

0.00022573626

5.749413e-05

5.30715e-05

2.94842e-06

1.47421e-06

0.00015939687

3.685526e-05

4.42263e-06

5.472794e-05

6.339104e-05

7.37105e-06

7.37105e-06

1.47421e-06

1.47421e-06

0.0204856631800006

0.00014160936

0.00014160936

4.69593e-06

4.69593e-06

0.00013691343

2.65761e-06

0.00013425582

6.039211e-05

6.039211e-05

4.42263e-06

4.42263e-06

7.37105e-06

7.37105e-06

4.859843e-05

3.640263e-05

1.21958e-05

0.00023494685

0.00023494685

0.00023494685

0.00023494685

0.00138374552000001

0.00138374552000001

8.84525e-06

8.84525e-06

5.89684e-06

5.89684e-06

8.024158e-05

5.665424e-05

2.358734e-05

9.39187e-06

9.39187e-06

0.000530341430000001

0.000525918800000001

4.42263e-06

0.000713082200000002

0.000713082200000002

2.211314e-05

2.211314e-05

1.383321e-05

1.383321e-05

0.00165419836000001

4.597382e-05

2.506157e-05

1.179368e-05

1.326789e-05

4.42263e-06

4.42263e-06

2.94842e-06

1.47421e-06

1.47421e-06

1.47421e-06

1.47421e-06

7.37105e-06

1.47421e-06

5.89684e-06

4.69594e-06

4.69594e-06

0.00159937928000001

7.636805e-05

8.84526e-06

1.47421e-06

6.604858e-05

0.000576252800000002

0.000539542970000001

3.670983e-05

0.000426869659999999

2.063895e-05

4.42263e-06

9.39188e-06

0.000392416199999999

0.000377218029999999

0.000377218029999999

2.326011e-05

2.326011e-05

0.00011941063

0.00011941063

8.84526e-06

8.84526e-06

8.84526e-06

0.0003347947

0.0003347947

1.14695e-06

1.14695e-06

4.422622e-05

4.422622e-05

0.00010514529

6.546692e-05

3.967837e-05

1.916472e-05

1.916472e-05

0.00016511152

0.00016511152

1.515633e-05

1.515633e-05

5.68362e-06

5.68362e-06

9.47271e-06

9.47271e-06

2.619452e-05

2.619452e-05

2.462921e-05

2.462921e-05

1.56531e-06

1.56531e-06

0.00925895975999994

0.00760647231999988

0.00700009918999989

0.00258330135999998

0.00037091954

0.00261934252999999

0.00142653576

4.16668e-05

2.850485e-05

1.316195e-05

0.000564706329999999

0.000564706329999999

4.51373e-06

4.51373e-06

4.51373e-06

5.89683e-06

5.89683e-06

5.89683e-06

0.00164051157000001

0.00164051157000001

1.56531e-06

0.00163894626000001

1.56531e-06

1.56531e-06

1.56531e-06

0.00186285187000001

1.2214e-05

1.2214e-05

1.031946e-05

1.89454e-06

5.882291e-05

5.882291e-05

5.882291e-05

0.000525674120000001

0.0004006571

0.0004006571

0.00012501702

0.00012501702

9.88631399999999e-05

9.88631399999999e-05

9.88631399999999e-05

0.000959414210000004

0.00012966141

4.319215e-05

8.646926e-05

0.000319602259999999

0.00016220476

1.179368e-05

0.00014560382

0.000306708399999999

7.497788e-05

9.95213099999999e-05

0.00013220921

0.00020344214

0.00020344214

0.00020786349

4.42263e-06

4.42263e-06

2.800993e-05

2.800993e-05

0.00017543093

5.89684e-06

0.00016953409

0.000592416990000002

0.000592416990000002

1.47421e-06

1.47421e-06

8.84525e-06

7.37104e-06

1.47421e-06

6.64402e-06

6.64402e-06

0.00027715138

0.00027715138

3.193046e-05

3.060166e-05

1.3288e-06

2.65761e-06

2.65761e-06

5.46062e-06

5.46062e-06

0.00010851297

8.901487e-05

1.94981e-05

8.84526e-06

8.84526e-06

0.00013159358

3.538101e-05

5.159727e-05

4.46153e-05

9.30163e-06

9.30163e-06

6.7375e-06

1.47421e-06

1.47421e-06

1.47421e-06

1.47421e-06

1.47421e-06

1.47421e-06

3.78908e-06

3.78908e-06

3.78908e-06

0.00491365930999993

0.000406553909999999

0.000406553909999999

9.81382e-06

0.00038288879

1.38513e-05

0.00300493687999999

0.00063568745

0.00048996722

0.00014572023

0.000879209060000001

0.000879209060000001

0.00037633143

0.00029595731

7.847958e-05

1.89454e-06

1.56531e-06

1.56531e-06

0.00101042801

9.95377e-05

5.45527e-05

0.000293292119999999

0.00056304549

1.282038e-05

3.13062e-06

9.68976e-06

6.385023e-05

1.689083e-05

4.69594e-05

2.504501e-05

2.504501e-05

0.00022924866

3.921611e-05

3.921611e-05

3.78908e-06

3.78908e-06

0.00018624347

0.00018624347

0.00016075999

0.00016075999

0.00016075999

0.00012765023

0.00012765023

4.492017e-05

8.273006e-05

3.929451e-05

1.89454e-06

1.89454e-06

3.739997e-05

3.739997e-05

0.000691730930000001

1.063043e-05

1.063043e-05

0.00013110481

0.00013110481

0.000346990739999999

4.169932e-05

0.00030529142

0.00013267915

4.42263e-06

0.00012825652

3.199638e-05

7.37104e-06

2.462534e-05

3.832942e-05

3.832942e-05

0.00019526574

9.77383299999999e-05

6.35935e-05

4.42263e-06

2.97222e-05

4.878562e-05

4.878562e-05

6.64401e-06

6.64401e-06

4.209778e-05

3.03952e-06

3.905826e-05

5.508783e-05

5.508783e-05

1.762851e-05

3.745932e-05

3.13063e-06

3.13063e-06

3.13063e-06

0.000808229110000002

1.621631e-05

1.621631e-05

1.621631e-05

1.621631e-05

0.000278987599999999

0.000278987599999999

0.000278987599999999

0.000278987599999999

0.0005130252

0.0005130252

0.0005130252

0.0005130252

0.00109504619

1.47421e-05

1.47421e-05

1.47421e-05

1.47421e-05

0.00106113937

0.00106113937

0.00106113937

0.000298973709999999

2.800999e-05

0.00020196671

0.000532188960000001

1.916472e-05

1.916472e-05

1.916472e-05

1.47421e-06

8.84526e-06

8.84525e-06

0.00365428810000002

0.00365428810000002

0.00365428810000002

0.00365428810000002

0.00240787521000001

0.00016506404

0.00103291312

4.843573e-05

3.443692e-05

3.443692e-05

3.443692e-05

2.347972e-05

2.347972e-05

3.13063e-06

3.13063e-06

7.82657e-06

3.13063e-06

4.69594e-06

0.00582053818999977

0.00026919871

0.00026919871

0.00026919871

0.00026919871

0.00422860706999997

4.275202e-05

4.275202e-05

4.275202e-05

0.000386078369999999

0.00017702347

0.00017702347

3.642071e-05

3.642071e-05

0.00011283804

0.00011283804

5.979615e-05

5.979615e-05

0.00018067296

8.84526e-06

8.84526e-06

2.948418e-05

2.948418e-05

0.00014234352

0.00014234352

0.00128833484

0.00128833484

0.00128833484

0.000524177540000001

0.000524177540000001

0.0003888373

1.563469e-05

1.326792e-05

0.00010643763

4.430805e-05

4.430805e-05

4.430805e-05

0.000814312220000002

0.00044490344

0.00044490344

1.017406e-05

1.017406e-05

0.000315008399999999

0.000315008399999999

2.948423e-05

2.948423e-05

1.474209e-05

1.474209e-05

0.000919019600000002

0.000919019600000002

0.000598001850000001

0.0002112318

2.948421e-05

7.800784e-05

2.2939e-06

2.600305e-05

2.600305e-05

2.600305e-05

2.94842e-06

2.94842e-06

2.94842e-06

0.00132273241000001

0.00101941086

0.000435749809999999

0.000435749809999999

0.000583661050000001

0.000583661050000001

6.59032e-05

6.59032e-05

6.59032e-05

0.00023741835

0.00023741835

0.00021708667

1.148643e-05

8.84525e-06

0.00015297269

0.00015297269

0.00015297269

0.00015297269

0.00015297269

5.849472e-05

1.47421e-06

1.47421e-06

1.47421e-06

1.47421e-06

4.27722e-06

4.27722e-06

4.27722e-06

4.27722e-06

5.274329e-05

5.274329e-05

5.274329e-05

5.274329e-05

0.11454788853001

0.0238952112600021

0.00306960308000002

4.726582e-05

5.89683e-06

5.89683e-06

4.136899e-05

4.136899e-05

0.00019901823

0.00019901823

0.00019901823

0.00177306193000001

2.94842e-06

2.94842e-06

0.000819537270000001

0.000819537270000001

3.282161e-05

1.47421e-06

3.13474e-05

0.000485802509999999

0.00031105769

8.956803e-05

2.620846e-05

5.896833e-05

0.00016363061

7.37105e-06

5.89684e-06

0.00015036272

0.00013310839

5.969926e-05

1.47421e-06

7.193492e-05

0.00012341945

5.5896e-06

4.113413e-05

7.669572e-05

1.179367e-05

8.84525e-06

2.94842e-06

0.00011016741

8.11725599999999e-05

4.275202e-05

1.47421e-06

3.694633e-05

2.899485e-05

2.899485e-05

0.00017245074

0.00017245074

0.00017245074

0.000767638950000002

1.89454e-06

1.89454e-06

3.390681e-05

2.948418e-05

1.47421e-06

2.94842e-06

0.00022113144

1.179371e-05

0.0001120399

9.729783e-05

3.22334e-06

1.89454e-06

1.3288e-06

7.64436e-06

7.64436e-06

0.00049983846

5.75143e-06

0.00015239521

1.179371e-05

0.00012341749

0.00020648062

1.095719e-05

1.095719e-05

1.095719e-05

1.095719e-05

0.00813043442999974

5.89683e-06

5.89683e-06

5.89683e-06

9.01485e-05

6.361274e-05

3.542057e-05

3.13062e-06

2.506155e-05

4.42263e-06

4.42263e-06

1.474209e-05

1.474209e-05

7.37104e-06

7.37104e-06

0.00276431241000002

5.153142e-05

5.153142e-05

1.56531e-06

1.56531e-06

1.163159e-05

1.163159e-05

9.39187e-06

9.39187e-06

0.00269019222000002

1.17937e-05

1.47421e-06

0.0001203919

8.84526e-06

0.00017211136

0.00234804899000001

2.75268e-05

4.42263e-06

4.42263e-06

4.42263e-06

0.00200956299000002

0.00200956299000002

0.00200956299000002

4.27722e-06

2.94842e-06

2.94842e-06

1.3288e-06

1.3288e-06

0.00325181385

0.00306635421

0.00306635421

5.89684e-06

5.89684e-06

0.0001795628

0.0001795628

4.42263e-06

4.42263e-06

4.42263e-06

4.42263e-06

0.00616466549999982

6.044253e-05

1.47421e-06

1.47421e-06

1.769051e-05

1.769051e-05

4.127781e-05

4.127781e-05

3.128954e-05

3.128954e-05

2.6576e-06

2.863194e-05

2.932498e-05

1.795773e-05

1.795773e-05

1.136725e-05

1.136725e-05

0.00148898529000001

0.00014117844

0.00014117844

0.00013020134

0.00013020134

0.00100321145

0.00100321145

0.00021439406

0.00021439406

3.685526e-05

3.685526e-05

3.685526e-05

0.00015691334

2.2939e-06

2.2939e-06

7.518463e-05

7.371042e-05

1.47421e-06

7.943481e-05

7.943481e-05

1.8632e-06

1.8632e-06

1.8632e-06

2.94842e-06

2.94842e-06

2.94842e-06

8.32133299999999e-05

1.588904e-05

8.84525e-06

7.04379e-06

1.179367e-05

7.37104e-06

4.42263e-06

1.47421e-06

1.47421e-06

5.405641e-05

5.405641e-05

0.00024590544

3.325541e-05

3.325541e-05

2.94842e-06

1.47421e-06

1.47421e-06

0.00020970161

8.84526e-06

3.685525e-05

0.00016252689

1.47421e-06

0.00113848431

0.00113848431

0.00113848431

1.37634e-05

1.37634e-05

1.37634e-05

0.000804131930000001

7.45279e-06

7.45279e-06

2.049519e-05

5.58959e-06

1.49056e-05

2.94842e-06

2.94842e-06

2.211623e-05

2.211623e-05

1.47421e-06

1.47421e-06

0.000734972420000001

7.667868e-05

0.0006523969

2.94842e-06

2.94842e-06

1.467267e-05

1.14695e-06

1.352572e-05

1.163159e-05

2.94842e-06

2.94842e-06

8.68317e-06

8.68317e-06

0.00205301610000001

0.00033058901

2.948418e-05

0.00020344104

9.76637899999999e-05

7.347429e-05

7.347429e-05

0.00164895280000001

0.00136899882000001

8.84525e-06

1.017406e-05

0.00026093467

5.89684e-06

5.89684e-06

5.89684e-06

0.000934613620000001

2.62116e-06

1.47421e-06

1.47421e-06

1.14695e-06

1.14695e-06

2.94842e-06

2.94842e-06

2.94842e-06

0.000877362570000001

6.759512e-05

6.759512e-05

4.350204e-05

4.350204e-05

0.000750049110000001

0.000750049110000001

2.94842e-06

2.94842e-06

4.42263e-06

4.42263e-06

8.84525e-06

8.84525e-06

1.03225e-05

1.03225e-05

1.03225e-05

1.474213e-05

1.03195e-05

1.03195e-05

4.42263e-06

4.42263e-06

2.65761e-06

2.65761e-06

2.65761e-06

2.395923e-05

1.89454e-06

1.89454e-06

6.64402e-06

6.64402e-06

1.542067e-05

1.542067e-05

0.00108264317

2.94842e-06

2.94842e-06

2.94842e-06

0.00107969475

6.54036e-05

5.096876e-05

7.37104e-06

5.58959e-06

1.47421e-06

1.982233e-05

9.66495e-06

4.42263e-06

5.73475e-06

1.47421e-06

1.47421e-06

2.302702e-05

2.302702e-05

3.966436e-05

2.623131e-05

4.5878e-06

2.94842e-06

5.89683e-06

0.000930303230000002

0.00018429583

4.42263e-06

0.000365805549999999

2.344376e-05

0.000342016

1.031946e-05

0.00435457986999991

0.00435457986999991

4.5878e-06

4.5878e-06

0.000289088239999999

0.000289088239999999

1.916472e-05

1.916472e-05

0.0002157913

0.00020442405

9.47271e-06

1.89454e-06

2.65358e-05

2.65358e-05

0.00378636961000002

1.47421e-06

0.000829095310000006

1.024765e-05

0.00294555244000001

1.30424e-05

1.8632e-06

1.11792e-05

0.00014329177

0.00014329177

0.00014329177

0.00014329177

0.00014029389

0.00014029389

0.00014029389

0.00014029389

0.00014029389

1.47421e-06

1.47421e-06

1.47421e-06

1.47421e-06

1.47421e-06

0.00449430329999989

3.220646e-05

3.220646e-05

3.087766e-05

3.087766e-05

1.3288e-06

1.3288e-06

0.000434164819999999

0.00011646253

0.00011646253

0.00011646253

0.00015715886

0.00015715886

0.00015715886

2.94842e-06

2.94842e-06

2.94842e-06

8.84525e-06

8.84525e-06

8.84525e-06

8.84526e-06

8.84526e-06

8.84526e-06

0.00012678203

1.031947e-05

1.031947e-05

9.140102e-05

9.140102e-05

2.94842e-06

2.94842e-06

2.94842e-06

2.94842e-06

1.91647e-05

1.91647e-05

1.3288e-06

1.3288e-06

1.3288e-06

1.179367e-05

1.179367e-05

1.179367e-05

0.00344723024000003

4.0571e-05

1.47421e-06

1.47421e-06

3.98641e-06

3.98641e-06

3.511038e-05

3.511038e-05

3.243259e-05

1.76905e-05

1.76905e-05

1.474209e-05

1.474209e-05

2.94842e-06

2.94842e-06

2.94842e-06

9.07047699999999e-05

6.859163e-05

1.699428e-05

5.159735e-05

5.89683e-06

5.89683e-06

7.37105e-06

7.37105e-06

8.84526e-06

8.84526e-06

9.39131999999999e-05

7.37104e-06

7.37104e-06

2.063893e-05

2.063893e-05

6.442902e-05

6.442902e-05

1.47421e-06

1.47421e-06

0.00127253578000001

0.00127253578000001

0.00127253578000001

0.000551354330000001

1.326789e-05

1.326789e-05

0.000538086440000001

0.000288945079999999

0.00024766715

1.47421e-06

0.00024029605

7.37104e-06

7.37104e-06

2.653577e-05

2.653577e-05

0.00013710142

8.550409e-05

1.47421e-06

5.012312e-05

6.928782e-05

1.47421e-06

4.42263e-06

6.339098e-05

5.454571e-05

5.454571e-05

1.769051e-05

1.47421e-06

3.538099e-05

0.000533994730000001

0.000533994730000001

1.887391e-05

0.000515120820000001

6.044262e-05

1.621631e-05

1.621631e-05

4.422631e-05

4.422631e-05

0.00015164386

1.326788e-05

4.42263e-06

8.84525e-06

7.37105e-06

7.37105e-06

0.00013100493

1.312251e-05

7.37105e-06

3.97493e-05

7.076207e-05

0.00025251006

8.845249e-05

8.845249e-05

1.769054e-05

1.769054e-05

0.00014636703

0.00014636703

6.933712e-05

2.358735e-05

1.179368e-05

1.179367e-05

4.574977e-05

4.574977e-05

1.867525e-05

1.397931e-05

1.397931e-05

1.10852e-05

1.3288e-06

1.56531e-06

4.69594e-06

4.69594e-06

4.69594e-06

1.179367e-05

1.179367e-05

1.179367e-05

1.179367e-05

3.832946e-05

3.832946e-05

3.832946e-05

3.832946e-05

7.483954e-05

7.483954e-05

7.336533e-05

2.653577e-05

1.47421e-06

4.535535e-05

1.47421e-06

1.47421e-06

0.000435498549999999

0.00022391964

7.37104e-06

7.37104e-06

0.0002119608

1.179367e-05

0.00014414717

1.031947e-05

1.179368e-05

3.390681e-05

4.5878e-06

4.5878e-06

0.00021157891

0.00020715628

1.47421e-06

4.42263e-06

0.00012532754

1.47421e-06

7.445769e-05

4.42263e-06

4.42263e-06

1.56531e-06

1.56531e-06

1.56531e-06

1.56531e-06

7.82656e-06

7.82656e-06

7.82656e-06

7.82656e-06

7.82656e-06

0.0835388090999991

7.57816e-06

7.57816e-06

1.89454e-06

1.89454e-06

5.68362e-06

5.68362e-06

0.0004487605

0.0004487605

1.47421e-06

1.47421e-06

0.00015375272

2.64388e-05

0.00012731392

0.00029353357

4.358035e-05

0.00024995322

7.3298e-05

7.3298e-05

7.3298e-05

7.3298e-05

8.84526e-06

2.94842e-06

2.94842e-06

2.94842e-06

5.89684e-06

5.89684e-06

5.89684e-06

5.89684e-06

5.89684e-06

5.89684e-06

5.89684e-06

5.853218e-05

5.75143e-06

5.75143e-06

5.75143e-06

5.278075e-05

7.08024e-06

1.47421e-06

5.60603e-06

4.570051e-05

2.94842e-06

4.275209e-05

0.000940746940000001

0.000631002510000001

0.000631002510000001

5.28119e-05

0.00057704366

1.14695e-06

7.82656e-06

7.82656e-06

7.82656e-06

0.00011113713

0.00011113713

0.00011113713

0.00019078074

0.00017759978

1.721841e-05

0.00016038137

1.318096e-05

9.39188e-06

3.78908e-06

4.097777e-05

4.097777e-05

4.097777e-05

4.097777e-05

0.00291306539000001

1.47421e-06

1.47421e-06

1.47421e-06

5.229486e-05

2.434696e-05

3.78908e-06

2.055788e-05

2.79479e-05

2.79479e-05

2.94842e-06

2.94842e-06

2.94842e-06

0.00285634790000001

5.89684e-06

5.89684e-06

2.211314e-05

1.769051e-05

4.42263e-06

0.00128388716

7.4528e-06

1.3288e-06

0.000108694

0.00116641156

4.852465e-05

4.852465e-05

0.00106670298

0.0010435502

2.315278e-05

6.948076e-05

5.04515e-05

6.26125e-06

1.276801e-05

4.42263e-06

4.42263e-06

2.653581e-05

1.179368e-05

1.474213e-05

9.39187e-06

4.69593e-06

4.69594e-06

0.0001823412

0.0001823412

1.474212e-05

2.94842e-06

1.17937e-05

0.00012230874

4.42263e-06

0.00010849425

9.39186e-06

0.0530037861800035

0.0530037861800035

0.000863031370000005

0.000863031370000005

0.000490909030000001

0.00012919163

0.0003617174

7.45279e-06

7.45279e-06

4.69594e-06

1.56531e-06

3.13063e-06

0.000375296

0.000273075

0.000102221

0.000111148

0.000111148

0.0496255062000033

9.35745e-06

3.200139e-05

0.0241602176399997

0.02541160358

1.8632e-06

1.046294e-05

0.000336277940000001

0.000336277940000001

3.07086e-05

3.07086e-05

0.00115876031

0.0008907272

0.00026803311

0.00022557927

0.00022263085

7.97282e-06

7.97282e-06

1.328804e-05

9.30163e-06

3.98641e-06

1.996079e-05

1.996079e-05

0.0001814092

0.00017951466

1.89454e-06

2.94842e-06

2.94842e-06

2.94842e-06

0.0250859802899985

0.000908546060000001

0.000908546060000001

2.06389e-05

0.000759051620000001

1.14695e-06

4.69594e-06

6.26125e-06

5.68363e-06

4.370825e-05

5.149961e-05

3.13063e-06

1.47421e-06

1.125507e-05

0.0241774342299985

0.0221011374499985

0.00160109561

0.00209201464

0.00063777

5.31521e-06

0.00018738675

0.0173539199299991

0.00022363531

1.14695e-06

1.14695e-06

4.69594e-06

4.69594e-06

0.00207045388999999

0.00113897

1.46169e-05

0.00071766817

0.00019919882

1.56531e-06

1.56531e-06

1.56531e-06

1.56531e-06

6.332941e-05

6.332941e-05

6.332941e-05

4.18464e-05

2.148301e-05

7.37104e-06

7.37104e-06

7.37104e-06

1.47421e-06

5.89683e-06

0.000602770940000002

0.000593379070000002

3.431644e-05

3.431644e-05

0.000552801380000002

2.94842e-06

1.06304e-05

1.769052e-05

0.000462381489999999

3.13063e-06

5.601992e-05

6.26125e-06

6.26125e-06

1.56531e-06

1.56531e-06

1.56531e-06

7.82656e-06

7.82656e-06

7.82656e-06

5.072562e-05

1.073979e-05

8.84525e-06

8.84525e-06

1.89454e-06

1.89454e-06

3.998583e-05

3.998583e-05

1.345012e-05

2.653571e-05

0.00243606337000001

5.749417e-05

1.03195e-05

1.03195e-05

1.03195e-05

4.717467e-05

4.717467e-05

4.717467e-05

2.358734e-05

2.358734e-05

2.358734e-05

7.37105e-06

1.621629e-05

0.00204228334000001

0.00012773181

2.661036e-05

3.13062e-06

2.347974e-05

6.048396e-05

6.048396e-05

1.56531e-06

1.56531e-06

3.907218e-05

3.907218e-05

1.410734e-05

1.410734e-05

1.410734e-05

0.00021029004

1.76905e-05

1.76905e-05

2.94842e-06

2.94842e-06

0.00018602842

0.00018602842

3.6227e-06

3.6227e-06

0.000649626870000003

2.65761e-06

2.65761e-06

1.76905e-05

1.76905e-05

8.55177799999999e-05

8.55177799999999e-05

0.000536551230000001

0.000533602810000001

2.94842e-06

1.89454e-06

1.89454e-06

5.31521e-06

1.3288e-06

3.98641e-06

0.00104052728

1.47421e-06

1.47421e-06

5.30715e-05

4.570046e-05

7.37104e-06

2.65358e-05

2.65358e-05

3.538099e-05

3.538099e-05

5.454572e-05

5.454572e-05

2.94842e-06

2.94842e-06

0.00012815292

0.000104339

2.381392e-05

1.56531e-06

1.56531e-06

0.000723584500000003

0.000723584500000003

1.326791e-05

1.17937e-05

1.47421e-06

7.46215e-06

7.46215e-06

7.46215e-06

7.46215e-06

3.796022e-05

2.981119e-05

2.981119e-05

2.981119e-05

8.14903e-06

8.14903e-06

8.14903e-06

2.136943e-05

2.136943e-05

2.136943e-05

2.136943e-05

0.00016153008

0.00016153008

1.3288e-06

1.3288e-06

0.00016020128

8.34122e-06

0.00015186006

8.437664e-05

8.437664e-05

8.437664e-05

7.553138e-05

8.84526e-06

3.390684e-05

1.47421e-06

1.47421e-06

1.47421e-06

1.47421e-06

3.243263e-05

3.243263e-05

3.243263e-05

3.243263e-05

0.00010614301

0.00010614301

0.00010614301

0.00010614301

0.00010614301

0.00010614301

1.85831e-05

9.73785e-06

9.73785e-06

9.73785e-06

9.73785e-06

9.73785e-06

8.84525e-06

8.84525e-06

8.84525e-06

8.84525e-06

8.84525e-06

0.00711275712999969

0.00014845904

0.00014551062

0.00014551062

0.00014551062

0.00014551062

2.94842e-06

2.94842e-06

2.94842e-06

2.94842e-06

0.00384003819000004

0.00384003819000004

0.00384003819000004

7.960732e-05

3.685527e-05

4.275205e-05

0.000112549

0.000112549

0.000378164819999999

0.0002487051

0.00012945972

0.00129567069

0.00129567069

1.485331e-05

1.485331e-05

0.00010017525

1.47421e-06

6.184578e-05

3.685526e-05

0.00013382584

2.94842e-06

1.031946e-05

1.47421e-06

0.00010139324

1.769051e-05

1.523452e-05

1.031946e-05

4.91506e-06

0.00170544371000001

2.94842e-06

2.034813e-05

3.577006e-05

3.538105e-05

0.00133362585000001

2.94842e-06

0.00022724714

4.717464e-05

1.56531e-06

1.56531e-06

2.94842e-06

2.94842e-06

0.00312425990000002

3.066758e-05

3.066758e-05

3.066758e-05

7.08023e-06

2.358735e-05

0.00309169778000002

3.76811e-06

3.76811e-06

3.76811e-06

0.000852811500000002

0.000852811500000002

0.000852811500000002

1.769054e-05

1.769054e-05

1.769054e-05

0.000316328469999999

0.000316328469999999

0.000316328469999999

0.00180969822000001

0.00180969822000001

0.00180969822000001

7.37105e-06

7.37105e-06

7.37105e-06

8.402989e-05

8.402989e-05

8.402989e-05

1.89454e-06

1.89454e-06

1.89454e-06

1.89454e-06

1.47421e-06

1.47421e-06

1.47421e-06

1.47421e-06

1.47421e-06

1.47421e-06

0.723288898769161

0.703359061589218

1.660947e-05

1.660947e-05

1.660947e-05

1.660947e-05

4.66621e-06

4.66621e-06

3.192e-06

3.192e-06

1.47421e-06

1.47421e-06

0.703337785909218

0.00022241885

3.287157e-05

2.191438e-05

1.095719e-05

4.69593e-06

4.69593e-06

9.77465e-06

9.77465e-06

2.590078e-05

2.590078e-05

6.077639e-05

4.255592e-05

1.822047e-05

3.13063e-06

3.13063e-06

2.504499e-05

2.504499e-05

3.13063e-06

3.13063e-06

2.675657e-05

3.13062e-06

1.579939e-05

7.82656e-06

4.69594e-06

4.69594e-06

1.781421e-05

1.781421e-05

7.82656e-06

7.82656e-06

2.362594e-05

7.82656e-06

7.82656e-06

1.56531e-06

1.56531e-06

1.157647e-05

1.157647e-05

1.3288e-06

1.3288e-06

1.3288e-06

1.3288e-06

0.452991900439913

3.13063e-06

3.13063e-06

0.451755739669917

0.01785075417

0.00154898911000001

3.01015e-06

0.141972007180065

5.580972e-05

0.290325169339966

5.601992e-05

5.601992e-05

1.56531e-06

1.56531e-06

1.3288e-06

1.3288e-06

0.000918061200000008

0.000918061200000008

1.60525e-05

1.23261e-05

3.7264e-06

0.00024000241

3.169149e-05

0.00020831092

1.56531e-06

1.56531e-06

1.56531e-06

4.677886e-05

4.677886e-05

6.64402e-06

2.135109e-05

1.878375e-05

8.573659e-05

1.8632e-06

1.8632e-06

1.56531e-06

1.56531e-06

1.181297e-05

1.181297e-05

1.001115e-05

4.69594e-06

5.31521e-06

6.048396e-05

3.13063e-06

3.98641e-06

5.336692e-05

8.653295e-05

8.653295e-05

8.653295e-05

0.249469278710079

0.196560400880081

1.328803e-05

0.000566720340000001

4.69593e-06

0.00015015482

1.136725e-05

1.3288e-06

3.67024e-05

0.11917193640002

0.0766042069100359

1.378123e-05

1.378123e-05

2.2939e-06

2.2939e-06

0.000474312499999998

0.000474312499999998

0.0227314615600002

0.00172336539000001

0.0209673980600001

3.443686e-05

6.26125e-06

0.0296870286399976

0.00229691649000001

0.027388783349998

1.3288e-06

4.69593e-06

4.69593e-06

4.69593e-06

7.37105e-06

1.47421e-06

1.47421e-06

5.89684e-06

5.89684e-06

0.000397881279999999

9.39188e-06

9.39188e-06

6.26125e-06

4.69594e-06

1.56531e-06

3.98641e-06

3.98641e-06

7.45279e-06

7.45279e-06

3.340333e-05

3.98641e-06

2.067991e-05

8.73701e-06

3.45985e-06

3.45985e-06

2.89411e-06

2.89411e-06

1.086693e-05

1.3288e-06

9.53813e-06

2.71226e-06

1.14695e-06

1.56531e-06

2.62836e-05

6.26125e-06

2.002235e-05

0.000291168869999999

0.000291168869999999

0.0194722756499993

0.00630651204999999

1.095719e-05

1.095719e-05

1.095719e-05

0.00019737796

1.56531e-06

1.56531e-06

0.00019581265

7.009e-05

7.15959e-05

5.412675e-05

0.00333578492999999

3.98641e-06

3.98641e-06

9.235343e-05

9.235343e-05

0.000871023350000003

9.30163e-06

4.903476e-05

0.00019557301

0.00061711395

0.00023033721

0.00023033721

0.00080484934

1.721844e-05

0.00013300051

0.00065463039

0.00014924458

2.65761e-06

7.24893e-06

0.00013933804

1.289338e-05

1.156458e-05

1.3288e-06

1.432678e-05

1.432678e-05

0.00100916751

0.00014089534

0.00011211428

0.00075615789

0.00014760294

0.00012099263

2.661031e-05

0.0011680536

0.000703218150000001

0.00036733218

7.462327e-05

0.0002612627

5.158345e-05

5.158345e-05

0.000413252

0.0004105944

2.6576e-06

1.791573e-05

1.14695e-06

1.14695e-06

1.676878e-05

1.676878e-05

0.00010133203

0.00010133203

0.00010133203

0.00147509061

3.242701e-05

3.242701e-05

1.69882e-05

1.69882e-05

6.523219e-05

6.523219e-05

0.000745551720000001

0.00018346248

6.15311e-06

0.00055593613

0.000614891490000001

0.00043704309

0.00017386199

3.98641e-06

0.0131305730800002

0.00387552548999999

0.00172501007000001

0.000346604489999999

0.00031102373

0.00106738185

0.000564014489999999

0.000564014489999999

1.14695e-06

1.14695e-06

7.16404e-05

7.16404e-05

0.000740102750000001

1.3288e-06

1.490558e-05

0.000723868370000001

0.000773610830000001

0.0002633771

0.00051023373

0.00310928163999999

0.00310928163999999

0.00310928163999999

7.37104e-06

7.37104e-06

7.37104e-06

0.00011226052

1.56531e-06

1.56531e-06

4.55215e-06

4.55215e-06

1.47421e-06

1.47421e-06

0.00010466885

0.00010024622

4.42263e-06

0.00394142439999998

0.00122672627

0.00122383216

1.3288e-06

1.56531e-06

3.13063e-06

3.13063e-06

8.72511e-06

3.04149e-06

5.68362e-06

7.867817e-05

2.470637e-05

5.39718e-05

0.00138474912

4.69594e-06

0.000309171569999999

0.00107088161

0.00114527797

0.00114527797

3.261449e-05

3.261449e-05

1.89454e-06

1.89454e-06

5.96281e-05

1.56531e-06

5.806279e-05

0.00207337004

6.64401e-06

5.31521e-06

1.3288e-06

1.56531e-06

1.56531e-06

3.47661e-05

1.095719e-05

2.380891e-05

9.261727e-05

9.261727e-05

1.14695e-06

1.14695e-06

0.00192732877000001

9.11857e-06

2.94842e-06

3.98641e-06

1.47421e-06

1.56531e-06

2.94842e-06

0.00190528743000001

9.30163e-06

2.65761e-06

6.64402e-06

1.133995e-05

1.133995e-05

8.20933e-06

3.13062e-06

7.97283e-06

7.97283e-06

7.97283e-06

7.97283e-06

2.721769e-05

2.721769e-05

2.721769e-05

2.721769e-05

1.026552e-05

1.026552e-05

1.026552e-05

1.026552e-05

1.026552e-05

0.000447296009999999

0.000447296009999999

0.000447296009999999

0.000394415679999999

8.62943999999999e-05

0.00030812128

5.288033e-05

5.173338e-05

1.14695e-06

0.0136383807199992

0.00021965737

0.00021965737

0.00021965737

0.00021965737

0.00021965737

5.307154e-05

1.47421e-06

1.47421e-06

1.47421e-06

1.47421e-06

3.980366e-05

3.980366e-05

3.980366e-05

3.980366e-05

1.179367e-05

1.179367e-05

1.179367e-05

1.179367e-05

0.000465413909999999

0.000462465489999999

0.00022954044

0.00022954044

0.00022954044

3.685526e-05

3.685526e-05

3.685526e-05

0.00019606979

0.00019606979

0.00019606979

2.94842e-06

2.94842e-06

2.94842e-06

2.94842e-06

0.000670453200000002

1.47421e-06

1.47421e-06

1.47421e-06

1.47421e-06

0.00037329683

0.00037329683

0.00037329683

0.00037329683

1.47421e-06

1.47421e-06

1.47421e-06

1.47421e-06

0.00022509889

0.00022509889

0.00022509889

0.00022509889

1.89454e-06

1.89454e-06

1.89454e-06

1.89454e-06

6.721452e-05

6.721452e-05

6.721452e-05

6.721452e-05

7.938186e-05

4.42263e-06

4.42263e-06

4.42263e-06

4.42263e-06

5.198635e-05

5.198635e-05

5.198635e-05

5.198635e-05

2.297288e-05

2.297288e-05

2.297288e-05

2.297288e-05

0.00331008871000002

0.00034465759

0.00034465759

0.00034465759

0.00034465759

0.00252336620000002

0.00252336620000002

0.00252336620000002

0.00252336620000002

0.00044206492

0.00044206492

0.00044206492

0.00044206492

1.179368e-05

8.84526e-06

8.84526e-06

8.84526e-06

8.84526e-06

2.94842e-06

2.94842e-06

2.94842e-06

2.94842e-06

1.474209e-05

1.474209e-05

1.474209e-05

1.474209e-05

1.474209e-05

0.00248849384000002

0.0002047697

6.781363e-05

6.781363e-05

6.781363e-05

1.769052e-05

1.769052e-05

1.769052e-05

4.997771e-05

4.997771e-05

3.09584e-05

1.474209e-05

4.27722e-06

6.928784e-05

6.928784e-05

6.928784e-05

0.000423097729999999

0.000423097729999999

0.000423097729999999

0.000358232469999999

6.486526e-05

0.00178232770000001

6.633947e-05

6.633947e-05

6.339105e-05

2.94842e-06

0.00129246456000001

0.000493714699999999

0.000493714699999999

4.42263e-06

4.42263e-06

1.769052e-05

1.769052e-05

0.000772214080000003

0.000772214080000003

4.42263e-06

4.42263e-06

0.00042352367

0.00042352367

0.00042352367

7.829871e-05

7.829871e-05

7.829871e-05

7.829871e-05

0.000371435879999999

0.000371435879999999

0.000371435879999999

8.84525e-06

8.84525e-06

0.000355364989999999

4.777645e-05

1.47421e-06

0.00030611433

7.22564e-06

7.22564e-06

0.000310374119999999

2.94842e-06

2.94842e-06

2.94842e-06

2.94842e-06

0.00023961209

7.728348e-05

7.728348e-05

7.728348e-05

0.00015495757

0.00015495757

0.00015495757

7.37104e-06

7.37104e-06

7.37104e-06

6.781361e-05

6.781361e-05

6.781361e-05

6.781361e-05

2.211315e-05

2.211315e-05

2.211315e-05

2.211315e-05

1.47421e-06

2.94842e-06

1.621631e-05

1.47421e-06

0.00024261295

0.00024261295

0.00024261295

0.00024261295

0.00024261295

0.000882788050000002

0.00059919387

0.00059919387

0.00059919387

0.00059919387

2.94842e-06

2.94842e-06

2.94842e-06

2.94842e-06

0.00028064576

0.00028064576

0.00028064576

0.00028064576

0.00449596036999986

0.000381093249999999

0.00011498827

0.00011498827

0.00011498827

2.94842e-06

2.94842e-06

2.94842e-06

7.37104e-06

7.37104e-06

7.37104e-06

0.00025578552

0.00025578552

0.00025578552

0.00022555352

0.00022555352

5.89684e-06

5.89684e-06

0.00021818247

0.00021818247

1.47421e-06

1.47421e-06

0.00388046834000002

4.597649e-05

4.597649e-05

4.597649e-05

0.00206386732000001

0.00206386732000001

0.00206386732000001

1.47421e-06

1.47421e-06

1.47421e-06

0.00036369355

0.00036369355

0.00036369355

1.474209e-05

1.474209e-05

1.474209e-05

0.00139071468000001

0.00139071468000001

0.00139071468000001

8.84526e-06

8.84526e-06

8.84526e-06

8.84526e-06

5.705796e-05

2.609955e-05

2.609955e-05

2.609955e-05

2.609955e-05

2.609955e-05

3.095841e-05

3.095841e-05

3.095841e-05

3.095841e-05

3.095841e-05

7.507379e-05

1.769051e-05

1.769051e-05

1.769051e-05

1.769051e-05

1.769051e-05

1.463119e-05

1.463119e-05

1.463119e-05

1.463119e-05

1.463119e-05

3.095842e-05

3.095842e-05

3.095842e-05

3.095842e-05

3.095842e-05

1.179367e-05

1.179367e-05

1.179367e-05

1.179367e-05

1.179367e-05

1.040959e-05

6.65185e-06

6.65185e-06

6.65185e-06

6.65185e-06

6.65185e-06

3.75774e-06

3.75774e-06

3.75774e-06

3.75774e-06

3.75774e-06

0.000688208700000001

1.76905e-05

1.76905e-05

1.76905e-05

1.76905e-05

1.76905e-05

0.00011685156

0.00011685156

0.00011685156

0.00011685156

0.00011685156

0.000553666640000002

0.000553666640000002

0.000553666640000002

0.000553666640000002

0.000553666640000002

0.00108053759

0.00108053759

2.94842e-06

2.94842e-06

2.94842e-06

2.94842e-06

1.47421e-06

1.47421e-06

1.47421e-06

1.47421e-06

0.00107611496

0.000980109130000002

0.000980109130000002

0.00019017249

3.13063e-06

2.96524e-05

0.00011200628

0.00021090292

0.000434244409999999

9.60058299999999e-05

3.832942e-05

2.948416e-05

8.84526e-06

5.325378e-05

2.94842e-06

1.179367e-05

2.966643e-05

5.89684e-06

2.94842e-06

4.42263e-06

4.42263e-06

0.00900483519999968

0.00017552194

0.00017552194

0.00017552194

0.00017552194

0.00017552194

0.00016713902

0.00016713902

0.00016713902

0.00016713902

5.89683e-06

3.078386e-05

0.00013045833

0.00773763316999973

0.00773763316999973

0.00029573387

0.00029573387

0.00029573387

0.00116424788

2.94842e-06

2.94842e-06

1.47421e-05

1.47421e-05

0.00102292351

0.00102292351

0.00012363385

0.00012363385

0.000657045100000002

0.000657045100000002

0.000657045100000002

0.00562060631999981

0.00562060631999981

0.00038296929

6.6194e-05

0.00018104402

0.00339969239000001

0.00159070662000001

0.000317957079999999

0.000317957079999999

0.000317957079999999

3.13063e-06

3.13063e-06

1.47421e-06

1.47421e-06

5.89684e-06

5.89684e-06

1.47421e-06

1.47421e-06

0.00012170504

8.84525e-06

5.536563e-05

5.749416e-05

6.486522e-05

1.47421e-06

1.47421e-06

6.19168e-05

0.00011941093

0.00011941093

0.000606583990000002

1.47421e-06

1.47421e-06

1.47421e-06

1.47421e-06

3.243257e-05

3.243257e-05

3.243257e-05

3.243257e-05

0.000551617940000002

0.000551617940000002

0.000551617940000002

0.000551617940000002

2.105927e-05

2.105927e-05

2.105927e-05

2.105927e-05

8.99267599999999e-05

8.99267599999999e-05

8.99267599999999e-05

8.84525499999999e-05

2.506156e-05

2.506156e-05

6.044257e-05

6.044257e-05

2.94842e-06

2.94842e-06

1.47421e-06

1.47421e-06

1.47421e-06

4.42263e-06

4.42263e-06

4.42263e-06

4.42263e-06

4.42263e-06

4.42263e-06

0.00059615134

0.00059615134

0.00059615134

0.00059615134

0.00059615134

0.00059615134

0.00059615134
