# Supplementary material for: Succession of bacterial communities on carrion is independent of vertebrate scavengers
Source: PeerJ. 2020 Jun 10;8:e9307. doi: 10.7717/peerj.9307 (PMC7293191; doi:10.7717/peerj.9307)
Supplement: Dataset S1 — Interactive Krona plots, and the full count table showing the distribution of each OTU among all samples. [file peerj-08-9307-s001.zip › DS3_COW.active_decay_stage_krona.html]

Javascript must be enabled to view this page.

members
magnitude
magnitudeUnassigned

COW.week\_3\_4\_5\_krona

1.00000002785451

0.99997088147251

2.61149e-06

2.61149e-06

2.61149e-06

2.61149e-06

2.61149e-06

2.61149e-06

1.2319547e-05

3.72056e-07

3.72056e-07

3.72056e-07

3.72056e-07

3.72056e-07

2.328107e-06

2.328107e-06

2.328107e-06

2.328107e-06

2.328107e-06

9.619384e-06

9.619384e-06

9.619384e-06

9.619384e-06

9.619384e-06

0.0228869507839996

0.001012225992

0.001012225992

0.000292536305

9.6661323e-05

9.4369763e-05

7.55957e-07

1.535603e-06

7.5040628e-05

4.5844509e-05

2.664978e-05

2.546339e-06

2.1291115e-05

2.687303e-06

1.5626356e-05

2.977456e-06

5.1751828e-05

5.1751828e-05

7.4614e-06

7.4614e-06

4.0330011e-05

3.83901e-07

3.834247e-05

1.213422e-06

3.90218e-07

4.2414384e-05

7.44112e-07

7.44112e-07

5.20878e-06

5.20878e-06

3.6461492e-05

3.6461492e-05

9.09198809999999e-05

2.97645e-06

2.97645e-06

7.5293529e-05

7.5293529e-05

1.2649902e-05

1.2649902e-05

1.488224e-06

1.488224e-06

1.488224e-06

0.000584867197999998

1.896208e-06

1.896208e-06

0.00015895319

4.6403334e-05

2.680785e-06

4.8644046e-05

6.12250250000001e-05

4.3638e-07

4.3638e-07

5.88771100000001e-05

5.88771100000001e-05

8.67407599999999e-05

6.5576546e-05

7.44112e-07

1.998665e-05

4.33452e-07

2.042945e-06

2.042945e-06

8.25519969999999e-05

1.6370464e-05

2.844924e-05

3.6016257e-05

1.716036e-06

0.000183888410999999

0.000183888410999999

8.30602e-07

4.58546e-07

3.72056e-07

2.976446e-06

2.976446e-06

5.673149e-06

3.72056e-07

5.301093e-06

0.0174730668529971

0.0174730668529971

1.77517e-06

4.58546e-07

4.58546e-07

1.316624e-06

4.27186e-07

8.89438e-07

3.2864085e-05

3.0621836e-05

7.67801e-07

2.9854035e-05

2.242249e-06

2.242249e-06

0.000271293833

0.000162577059

0.000152595645

9.981414e-06

3.544158e-06

4.45621e-07

7.67801e-07

2.330736e-06

1.193351e-06

1.193351e-06

0.000103979265

9.10035049999999e-05

1.297576e-05

0.0168834092789959

0.0168834092789959

3.6098311e-05

0.000681275768999993

0.0161660351989969

1.803896e-06

1.803896e-06

4.58546e-07

1.34535e-06

0.000281920589999999

0.000281920589999999

0.000281920589999999

0.00361907119199992

0.00361907119199992

0.00352908227399992

8.262384e-06

8.262384e-06

0.000671905230000005

4.8298436e-05

1.908672e-06

2.23842e-06

5.0620293e-05

5.603699e-06

3.83901e-07

7.56971e-07

0.000551946083000002

4.608435e-06

5.54032e-06

0.00154225295299998

1.5356e-06

5.07006349999999e-05

0.00149001671799998

3.626051e-05

3.626051e-05

0.000329405209

4.1372005e-05

0.000287265402

7.67802e-07

9.62987750000001e-05

9.62987750000001e-05

3.716886e-06

2.849982e-06

8.66904e-07

1.0943656e-05

1.0943656e-05

5.347453e-06

1.336863e-06

4.01059e-06

9.6786756e-05

9.6786756e-05

0.000321964361999999

0.000321964361999999

2.68731e-06

2.68731e-06

0.000319740953999999

2.1893972e-05

0.000297846981999999

9.597515e-06

9.597515e-06

5.9707993e-05

5.9707993e-05

1.151702e-06

1.151702e-06

1.3052626e-05

1.3052626e-05

8.90702080000001e-05

8.45107860000001e-05

7.636491e-06

1.345348e-06

7.55289470000001e-05

7.67802e-07

7.67802e-07

3.79162e-06

1.48822e-06

2.3034e-06

9.1871e-07

9.1871e-07

9.1871e-07

0.000756432192999996

6.54377e-07

2.20925e-07

2.20925e-07

2.20925e-07

4.33452e-07

4.33452e-07

4.33452e-07

2.604392e-06

2.604392e-06

2.604392e-06

2.604392e-06

8.09542380000001e-05

8.09542380000001e-05

2.978425e-06

2.978425e-06

7.1650857e-05

5.21387e-06

4.9703943e-05

1.6733044e-05

6.324956e-06

6.324956e-06

0.000672219185999998

0.000136518307

3.9290919e-05

3.0880638e-05

4.22291e-06

4.187371e-06

1.151702e-06

1.151702e-06

4.45621e-07

4.45621e-07

9.56300650000001e-05

1.0696083e-05

8.49339820000001e-05

0.000495419020999998

1.552922e-06

1.552922e-06

7.41873159999998e-05

7.41873159999998e-05

2.830522e-05

3.348502e-06

2.3037214e-05

1.919504e-06

3.72056e-07

3.72056e-07

8.56433e-07

8.56433e-07

7.21926879999999e-05

6.84421189999999e-05

3.366668e-06

3.83901e-07

1.3777917e-05

7.080911e-06

6.697006e-06

7.4614e-06

5.22298e-06

2.23842e-06

0.000203924702999999

9.35793099999999e-05

2.1784294e-05

8.85610989999999e-05

6.60003439999999e-05

5.330808e-06

2.232338e-06

4.853e-07

1.1157516e-05

4.6335836e-05

4.58546e-07

1.2277842e-05

3.72056e-07

1.11617e-06

1.0789616e-05

1.451018e-05

7.441118e-06

1.860282e-06

5.20878e-06

4.45621e-07

4.45621e-07

4.45621e-07

3.390347e-06

2.081207e-06

2.20925e-07

1.860282e-06

1.30914e-06

1.30914e-06

3.644589e-05

3.644589e-05

3.644589e-05

2.6154554e-05

2.6154554e-05

2.6154554e-05

2.6154554e-05

2.6154554e-05

0.000202596675

3.5968384e-05

3.5968384e-05

3.5968384e-05

3.5968384e-05

3.5968384e-05

1.11617e-06

1.11617e-06

1.11617e-06

1.11617e-06

1.11617e-06

1.0826168e-05

1.0826168e-05

1.0826168e-05

1.0826168e-05

1.0826168e-05

0.000133022346

0.000133022346

0.000133022346

0.000133022346

0.000133022346

1.6082767e-05

1.6082767e-05

9.644732e-06

9.644732e-06

9.644732e-06

2.680929e-06

2.680929e-06

2.680929e-06

3.757106e-06

3.757106e-06

3.757106e-06

5.58084e-06

5.58084e-06

5.58084e-06

5.58084e-06

5.58084e-06

1.532635e-06

4.3638e-07

4.3638e-07

4.3638e-07

4.3638e-07

4.3638e-07

1.096255e-06

1.096255e-06

1.096255e-06

1.096255e-06

1.096255e-06

1.0477406e-05

8.79621e-06

8.79621e-06

8.79621e-06

8.79621e-06

8.79621e-06

1.681196e-06

1.681196e-06

1.681196e-06

1.681196e-06

1.681196e-06

0.00768634917899984

0.00398574415599998

4.0955759e-05

4.0955759e-05

2.604394e-06

2.604394e-06

3.8351365e-05

1.17065e-06

3.6830341e-05

3.50374e-07

0.00385022896699999

0.00385022896699999

4.056887e-05

4.056887e-05

2.232336e-06

2.232336e-06

4.624681e-06

4.624681e-06

0.000154612193

0.000154612193

1.2277846e-05

1.2277846e-05

4.6382943e-05

8.433231e-06

3.7949712e-05

1.1517e-06

1.1517e-06

4.470114e-05

3.72056e-07

2.4238092e-05

2.0090992e-05

3.7307e-06

3.7307e-06

1.6793242e-05

4.58546e-07

1.6334696e-05

1.2572563e-05

1.2572563e-05

0.00267808344199999

1.488224e-06

0.000131055935

1.86028e-06

6.633698e-06

4.451412e-05

0.00249253118499999

0.000832497310999998

2.4265e-07

0.000681176667999999

0.000128010562999999

2.306743e-05

9.619576e-06

9.619576e-06

9.619576e-06

3.037846e-06

6.58173e-06

2.7132919e-05

2.7132919e-05

2.7132919e-05

2.7132919e-05

1.9857251e-05

1.9857251e-05

1.9857251e-05

1.9857251e-05

3.7949684e-05

1.116168e-06

1.116168e-06

1.116168e-06

3.6833516e-05

3.6833516e-05

3.6833516e-05

5.274868e-06

5.274868e-06

5.274868e-06

5.274868e-06

5.274868e-06

1.185962e-06

1.185962e-06

1.185962e-06

1.185962e-06

1.185962e-06

0.003694144193

2.232332e-06

2.232332e-06

2.232332e-06

2.232332e-06

2.20925e-07

2.20925e-07

2.20925e-07

2.20925e-07

1.2385973e-05

1.2385973e-05

1.2385973e-05

1.2385973e-05

6.18783e-07

6.18783e-07

6.18783e-07

6.18783e-07

0.000118444539

0.000118444539

0.000118444539

0.000118444539

1.7582757e-05

1.7582757e-05

1.7582757e-05

1.7582757e-05

3.72056e-07

3.72056e-07

3.72056e-07

3.72056e-07

3.90218e-07

3.90218e-07

3.90218e-07

3.90218e-07

0.003164793591

0.003164793591

0.003164793591

0.003164793591

1.413808e-05

1.413808e-05

1.413808e-05

1.413808e-05

2.85589e-06

2.85589e-06

2.85589e-06

2.85589e-06

1.6144194e-05

1.6144194e-05

1.6144194e-05

1.6144194e-05

0.000150033251

0.000150033251

0.000150033251

0.000150033251

0.000191555552000001

0.000191555552000001

0.000191555552000001

0.000191555552000001

1.63194e-06

1.63194e-06

1.63194e-06

1.63194e-06

7.44112e-07

7.44112e-07

7.44112e-07

7.44112e-07

7.5769507e-05

7.5769507e-05

4.7631394e-05

4.7631394e-05

4.7631394e-05

4.7631394e-05

4.33452e-07

4.33452e-07

4.33452e-07

4.33452e-07

1.886033e-06

1.886033e-06

1.886033e-06

1.886033e-06

7.366197e-06

7.366197e-06

7.366197e-06

7.366197e-06

1.8452431e-05

1.8452431e-05

1.8452431e-05

1.800681e-05

4.45621e-07

0.000680370586999999

3.50374e-07

3.50374e-07

3.50374e-07

3.50374e-07

3.50374e-07

0.000643615589999999

0.00011356633

3.72056e-07

3.72056e-07

3.72056e-07

1.323004e-05

1.323004e-05

1.323004e-05

9.75861869999999e-05

9.75861869999999e-05

9.75861869999999e-05

4.58546e-07

4.58546e-07

4.58546e-07

1.919501e-06

1.919501e-06

1.919501e-06

0.000488479218999998

6.2375015e-05

6.2375015e-05

6.2375015e-05

3.348506e-06

3.348506e-06

3.348506e-06

1.7106891e-05

1.83418e-06

1.83418e-06

4.33452e-07

4.33452e-07

4.84991e-06

4.84991e-06

9.989349e-06

9.989349e-06

7.7771287e-05

7.44112e-07

7.44112e-07

6.2430682e-05

6.2430682e-05

4.550979e-06

4.550979e-06

5.208786e-06

5.208786e-06

4.836728e-06

4.836728e-06

0.000327877519999999

0.000199960744

2.1461801e-05

4.58546e-07

1.0417558e-05

0.000149764159

1.785868e-05

0.000127916776

0.000127916776

4.1570041e-05

4.1570041e-05

4.1570041e-05

2.2276205e-05

1.3394056e-05

3.5876e-06

2.31218e-06

3.6404623e-05

3.0985541e-05

3.72056e-07

3.72056e-07

3.72056e-07

6.697006e-06

6.697006e-06

6.697006e-06

2.23233e-06

2.23233e-06

2.23233e-06

2.1684149e-05

2.1684149e-05

2.1684149e-05

5.419082e-06

1.823946e-06

1.823946e-06

1.823946e-06

3.595136e-06

3.595136e-06

3.595136e-06

8.557288e-06

8.557288e-06

8.557288e-06

2.604392e-06

2.604392e-06

2.604392e-06

5.952896e-06

5.952896e-06

5.952896e-06

0.00180857453599998

0.00180857453599998

0.00180857453599998

0.00180857453599998

0.00180857453599998

0.00180857453599998

0.000848705828999997

0.000848705828999997

2.519661e-06

2.519661e-06

2.519661e-06

2.519661e-06

7.67801e-07

7.67801e-07

7.67801e-07

7.67801e-07

1.8243602e-05

8.570148e-06

8.570148e-06

8.570148e-06

8.929342e-06

8.929342e-06

8.929342e-06

7.44112e-07

7.44112e-07

7.44112e-07

0.000279079627999999

0.000184793441

0.000184793441

0.000184793441

3.0844314e-05

3.0844314e-05

3.0844314e-05

5.5672822e-05

5.5672822e-05

5.5672822e-05

7.769051e-06

7.769051e-06

7.769051e-06

0.000548095136999998

0.000548095136999998

0.000338832514999999

0.000338832514999999

0.000152061245

0.000107887628

6.366307e-06

1.6513654e-05

2.1293656e-05

5.7201377e-05

1.30036e-06

3.1110338e-05

4.635333e-06

2.0155346e-05

1.116168e-06

1.116168e-06

1.116168e-06

1.116168e-06

1.116168e-06

1.116168e-06

4.33452e-07

4.33452e-07

4.33452e-07

4.33452e-07

4.33452e-07

4.33452e-07

0.0138851605640004

0.000381518712999999

0.000381518712999999

0.000204301005

1.816496e-05

1.816496e-05

8.2697e-07

8.2697e-07

8.1294436e-05

8.1294436e-05

9.99369119999998e-05

9.99369119999998e-05

2.217447e-06

2.217447e-06

1.86028e-06

1.86028e-06

7.44112e-07

7.44112e-07

7.44112e-07

6.66598369999999e-05

6.66598369999999e-05

6.66598369999999e-05

3.72056e-07

3.72056e-07

3.72056e-07

6.73967389999999e-05

6.73967389999999e-05

3.86826e-07

4.58546e-07

1.86028e-06

2.1182194e-05

4.3508893e-05

4.2044964e-05

9.449644e-06

4.920652e-06

4.528992e-06

2.8009864e-05

4.55986e-07

2.1855432e-05

5.698446e-06

4.585456e-06

4.585456e-06

0.0120991815850002

4.8480722e-05

4.8480722e-05

4.6232038e-05

3.7307e-07

4.5858968e-05

2.248684e-06

4.07984e-07

7.2149e-07

1.11921e-06

5.381389e-06

5.381389e-06

5.381389e-06

4.93294e-06

4.48449e-07

3.4288257e-05

3.4288257e-05

8.28042e-07

8.28042e-07

1.4871733e-05

1.4871733e-05

1.8588482e-05

1.8129127e-05

4.59355e-07

9.46321299999997e-05

9.46321299999997e-05

9.46321299999997e-05

9.46321299999997e-05

0.001259378656

0.001259378656

7.73652e-07

7.73652e-07

9.11971e-07

9.11971e-07

2.336678e-05

1.717124e-06

1.9458051e-05

2.191605e-06

3.099242e-06

3.099242e-06

2.6701218e-05

2.6701218e-05

1.203467e-06

1.203467e-06

2.976446e-06

3.72056e-07

2.60439e-06

2.1525547e-05

2.1525547e-05

0.000452290141

7.649316e-06

0.000442693246

1.947579e-06

0.000695131074999997

0.000695131074999997

1.4802043e-05

1.3955839e-05

4.55986e-07

3.90218e-07

1.6597074e-05

7.44112e-07

1.5852962e-05

0.000480823193999999

1.4612357e-05

3.376932e-06

1.345423e-06

2.031509e-06

1.743587e-06

1.743587e-06

6.700084e-06

6.700084e-06

1.181506e-06

1.181506e-06

4.58546e-07

4.58546e-07

1.151702e-06

7.67801e-07

3.83901e-07

0.000465466724999999

2.8800765e-05

7.956163e-06

8.199428e-06

1.2645174e-05

0.00017613256

0.000162726226

1.3406334e-05

9.16382069999999e-05

6.269071e-06

8.72759e-07

6.986445e-06

3.72056e-07

7.73652e-07

7.63642239999999e-05

9.68377799999999e-05

9.68377799999999e-05

5.6041148e-05

5.4673188e-05

1.36796e-06

1.6016265e-05

1.4096765e-05

1.9195e-06

7.44112e-07

7.44112e-07

7.44112e-07

0.000159872214

0.000159872214

8.5525e-06

4.12361e-07

8.140139e-06

8.167141e-06

6.216053e-06

1.951088e-06

8.93470249999999e-05

2.9879555e-05

1.21155e-06

5.825592e-05

5.38055480000001e-05

5.38055480000001e-05

7.73652e-07

7.73652e-07

7.73652e-07

7.73652e-07

3.20982e-06

3.20982e-06

3.20982e-06

3.20982e-06

0.00428114533899997

0.00397874798399993

0.00376596931999993

0.000630336918

9.104e-06

3.11373e-06

4.472919e-06

4.58546e-07

3.7307e-07

7.00016669999999e-05

0.00203942196199999

0.001008686508

5.9747552e-05

6.487481e-06

5.3260071e-05

0.000153031112

0.000153031112

2.431224e-06

2.431224e-06

2.431224e-06

2.706253e-06

1.535603e-06

7.67801e-07

3.83901e-07

3.83901e-07

1.17065e-06

1.17065e-06

0.000261780133999999

0.000261780133999999

2.1284849e-05

1.151702e-06

0.000234922469

4.421114e-06

3.5479744e-05

3.5479744e-05

3.5479744e-05

0.00161519880300001

1.192337e-06

1.192337e-06

8.20281e-07

3.72056e-07

8.384839e-06

5.709379e-06

5.709379e-06

2.67546e-06

2.67546e-06

0.000170005628

3.72056e-07

3.72056e-07

0.000140090863

0.000140090863

2.9542709e-05

2.9542709e-05

6.77710719999999e-05

6.77710719999999e-05

6.77710719999999e-05

3.4763082e-05

7.44112e-07

7.44112e-07

1.8999444e-05

1.8999444e-05

1.5019526e-05

3.72056e-07

1.464747e-05

0.00133047745500001

0.000330350761000001

4.55986e-07

1.118196e-06

9.26122399999997e-05

0.000236164339

0.000427616151

0.00019973507

1.1022776e-05

0.000216858305

0.000407402767

8.3224702e-05

9.1871e-07

0.000111630523

0.000211628832

0.000165107776

0.000165107776

2.60439e-06

2.60439e-06

2.60439e-06

0.000675982644999997

0.000675982644999997

1.8554759e-05

4.952862e-06

1.3601897e-05

6.3269529e-05

1.7880727e-05

4.58546e-07

4.4930256e-05

4.3638e-07

4.3638e-07

1.0503038e-05

1.0503038e-05

0.000224662241

0.000224662241

4.55986e-07

4.55986e-07

3.0912625e-05

2.116531e-06

2.8796094e-05

0.000301993504

1.240403e-06

4.685237e-06

0.000283002112999999

1.1517e-06

1.1914051e-05

7.762e-06

7.762e-06

1.3498917e-05

4.206971e-06

4.544668e-06

4.747278e-06

3.933666e-06

4.3638e-07

1.631936e-06

1.86535e-06

2.6976695e-05

9.019757e-06

9.019757e-06

9.019757e-06

1.63194e-06

1.63194e-06

1.63194e-06

4.03604e-06

4.03604e-06

4.03604e-06

1.2288958e-05

1.2288958e-05

1.367958e-06

1.0921e-05

0.00341303806899996

0.000317597257

0.000316823605

1.3800908e-05

5.054413e-06

0.000294489094

3.47919e-06

7.73652e-07

7.73652e-07

0.001965490532

3.2837974e-05

2.4097634e-05

8.74034e-06

2.797296e-06

2.797296e-06

0.000339866782

0.000339866782

0.000231866936

0.000168237449

4.853e-07

6.3144187e-05

7.763622e-06

4.45621e-07

7.318001e-06

0.001306275071

2.0877701e-05

8.34408e-05

3.83901e-07

0.000357121736

0.000727707409000004

0.000116743524

1.6585536e-05

4.737222e-06

1.1848314e-05

2.3548889e-05

1.3228932e-05

1.0319957e-05

3.948426e-06

3.948426e-06

0.000669302452999997

4.33452e-07

4.33452e-07

3.467614e-06

8.66904e-07

2.60071e-06

3.72056e-07

3.72056e-07

8.572087e-06

8.572087e-06

3.4538798e-05

2.206038e-06

1.36796e-06

3.09648e-05

1.720776e-06

4.55986e-07

1.26479e-06

0.000391756464

0.000324247483

8.29522e-07

4.0719727e-05

7.73652e-07

2.518608e-05

5.989682e-06

8.15968e-07

5.173714e-06

2.039146e-06

2.039146e-06

0.000220412378

0.000220412378

5.416262e-05

5.416262e-05

5.416262e-05

2.6807416e-05

2.6807416e-05

4.360491e-06

8.17353e-07

2.1629572e-05

2.0713709e-05

2.681393e-06

2.681393e-06

1.8032316e-05

9.11971e-07

1.7120345e-05

6.83845099999999e-05

3.72056e-07

3.72056e-07

1.4390492e-05

1.4390492e-05

4.1509914e-05

1.5346912e-05

2.6163002e-05

4.464672e-06

4.464672e-06

3.72056e-07

3.72056e-07

7.27532e-06

6.15915e-06

1.11617e-06

0.00017131351

0.000140035824

7.56971e-07

8.97853889999999e-05

4.48159e-06

4.5011874e-05

1.4768902e-05

1.4768902e-05

1.6508784e-05

3.678243e-06

1.2830541e-05

3.9700157e-05

3.9700157e-05

7.498024e-06

1.62391e-06

3.0578223e-05

7.455275e-06

7.455275e-06

2.687301e-06

4.334522e-06

4.33452e-07

5.9470172e-05

5.9470172e-05

5.9470172e-05

6.485416e-06

6.485416e-06

5.693531e-06

7.91885e-07

6.155042e-06

5.387241e-06

5.387241e-06

7.67801e-07

7.67801e-07

1.7816549e-05

8.735997e-06

8.735997e-06

8.735997e-06

8.735997e-06

9.080552e-06

9.080552e-06

9.080552e-06

9.080552e-06

6.6640347e-05

7.44112e-07

7.44112e-07

7.44112e-07

7.44112e-07

6.5896235e-05

6.5896235e-05

6.5896235e-05

3.0587812e-05

1.004551e-05

1.2354232e-05

1.2908681e-05

0.000156611656

0.000156611656

0.000156611656

0.000156611656

0.000143029841

9.736131e-06

3.845684e-06

3.90218e-07

3.90218e-07

3.90218e-07

3.90218e-07

3.90218e-07

0.00114715044

1.3670757e-05

1.3670757e-05

1.3670757e-05

1.3670757e-05

0.001079164007

1.4928171e-05

1.4928171e-05

1.4928171e-05

8.66829639999999e-05

5.2464226e-05

5.2464226e-05

3.86826e-07

3.86826e-07

2.3054529e-05

2.3054529e-05

8.465201e-06

8.465201e-06

2.312182e-06

2.312182e-06

8.716783e-06

6.878189e-06

6.878189e-06

1.838594e-06

1.838594e-06

0.000288281486999999

0.000288281486999999

0.000288281486999999

8.73504e-07

8.73504e-07

8.73504e-07

0.000302128901

0.000302128901

0.000194826365999999

4.606314e-06

0.000102696221

4.58546e-07

4.58546e-07

4.58546e-07

0.000200625325

8.494561e-05

8.494561e-05

3.158713e-06

3.158713e-06

9.15224239999999e-05

9.15224239999999e-05

1.3604607e-05

1.3604607e-05

7.393971e-06

7.393971e-06

0.000172243792

0.000172243792

5.9373354e-05

1.36796e-06

9.08547779999999e-05

9.586919e-06

1.1060781e-05

4.224534e-06

3.478394e-06

3.478394e-06

7.4614e-07

7.4614e-07

5.4315676e-05

3.8084058e-05

2.3034e-06

2.3034e-06

8.193255e-06

8.193255e-06

2.638285e-05

2.638285e-05

7.67801e-07

7.67801e-07

4.36752e-07

4.36752e-07

5.595e-06

5.595e-06

5.595e-06

1.0636618e-05

1.0636618e-05

8.134331e-06

2.502287e-06

1.416856e-05

1.416856e-05

1.416856e-05

1.416856e-05

1.416856e-05

1.682496e-06

4.58546e-07

4.58546e-07

4.58546e-07

4.58546e-07

1.22395e-06

1.22395e-06

1.22395e-06

1.22395e-06

0.108926970692

3.4743809e-05

3.4743809e-05

3.4743809e-05

3.4743809e-05

5.18349e-06

2.9560319e-05

0.00680924066400003

0.00121027448

2.5645335e-05

7.97641e-06

7.97641e-06

1.7668925e-05

1.7668925e-05

3.8831218e-05

7.67802e-07

7.67802e-07

3.8063416e-05

3.8063416e-05

0.000489247996999999

5.33790020000001e-05

3.2507118e-05

2.0871884e-05

0.000279246487

0.000279246487

4.909383e-06

1.345423e-06

3.56396e-06

8.50288079999999e-05

2.9764424e-05

2.232336e-06

4.0593886e-05

4.55986e-07

3.348506e-06

8.63367e-06

4.6125867e-05

1.3006806e-05

3.3119061e-05

5.044e-06

5.044e-06

1.551445e-05

1.656083e-06

1.3858367e-05

4.9699008e-05

2.8483439e-05

1.5266024e-05

8.31411e-07

1.2386004e-05

2.1215569e-05

1.9970754e-05

1.244815e-06

5.1139171e-05

5.1139171e-05

5.1139171e-05

0.000555711750999999

3.72056e-07

3.72056e-07

9.366094e-06

4.36752e-07

8.929342e-06

8.17677e-07

8.17677e-07

3.833587e-06

1.819956e-06

2.013631e-06

1.86028e-06

1.86028e-06

0.000537107020999999

4.548604e-06

0.000147664042999999

1.2916844e-05

8.5460423e-05

0.000286517107

2.355036e-06

2.20925e-07

2.134111e-06

4.33452e-07

4.33452e-07

4.33452e-07

4.33452e-07

0.001533788282

3.72056e-07

3.72056e-07

3.72056e-07

3.2488404e-05

3.2488404e-05

2.674651e-05

5.357993e-06

3.83901e-07

0.000332958703999999

2.0197622e-05

2.0197622e-05

1.5380108e-05

1.5380108e-05

2.27993e-06

2.27993e-06

0.000295101044

1.488226e-06

7.401945e-06

1.36796e-06

0.000275045876

3.72056e-07

4.48449e-07

4.847988e-06

4.128544e-06

3.3485e-06

3.3485e-06

3.3485e-06

0.000661343088999997

0.000661343088999997

0.000661343088999997

3.72056e-07

3.72056e-07

3.72056e-07

0.000502905473

0.000467676752

0.000467676752

1.860282e-06

1.860282e-06

3.3368439e-05

3.3368439e-05

5.37224e-06

5.37224e-06

5.37224e-06

5.37224e-06

0.00250330067399996

2.232338e-06

2.232338e-06

3.72056e-07

1.860282e-06

0.000140210408

6.3254641e-05

8.17353e-07

6.2437288e-05

2.224865e-06

2.224865e-06

6.019536e-05

6.019536e-05

1.4535542e-05

4.609056e-06

9.926486e-06

8.96898e-07

8.96898e-07

4.48449e-07

4.48449e-07

0.000307123470999999

3.86826e-07

3.86826e-07

2.1788968e-05

2.1788968e-05

3.5700276e-05

3.5700276e-05

0.00023649205

0.00023649205

1.2755351e-05

1.2755351e-05

9.611281e-06

2.145612e-06

2.145612e-06

7.465669e-06

7.465669e-06

0.000146975279

2.169307e-06

1.33686e-06

8.32447e-07

9.26604609999999e-05

9.18926599999999e-05

7.67801e-07

1.782481e-06

4.45621e-07

1.33686e-06

5.036303e-05

5.036303e-05

3.72056e-07

3.72056e-07

3.72056e-07

2.7720441e-05

1.2803481e-05

1.2803481e-05

3.72056e-07

3.72056e-07

1.4544904e-05

1.4544904e-05

0.000104690136

5.057476e-06

5.057476e-06

9.963266e-05

4.095658e-06

5.580836e-06

8.9956166e-05

3.720556e-06

3.720556e-06

3.720556e-06

0.000388914492999999

0.000388914492999999

0.000388914492999999

7.441112e-06

7.069056e-06

3.72056e-07

6.697e-06

3.72056e-07

3.72056e-07

0.000230172432

5.745935e-06

9.17091e-07

1.619024e-06

3.20982e-06

2.564764e-05

2.564764e-05

0.000197008542

8.017359e-06

1.17065e-06

0.000171123222

1.3694248e-05

3.003063e-06

1.770315e-06

1.324694e-06

4.45621e-07

4.68158e-06

2.15314e-06

1.693785e-06

4.59355e-07

1.16048e-06

1.16048e-06

1.36796e-06

1.36796e-06

0.001126372337

0.000336671572999998

1.6072953e-05

8.830799e-06

3.72056e-07

4.6351682e-05

0.000158614229

0.000106429854

5.2695081e-05

5.2695081e-05

0.000737005682999997

8.96107e-07

0.000647055136999997

4.04737e-06

4.212871e-06

7.8598196e-05

2.196002e-06

2.165856e-06

3.72056e-07

3.72056e-07

1.7938e-06

1.7938e-06

0.000195038631

3.72056e-07

3.72056e-07

3.72056e-07

0.00018456142

1.333279e-06

1.333279e-06

7.4588733e-05

7.4588733e-05

0.000100827092

0.000100827092

6.615538e-06

6.615538e-06

3.72056e-07

3.72056e-07

8.24722e-07

8.24722e-07

1.11617e-06

1.11617e-06

1.11617e-06

2.055279e-06

2.055279e-06

1.309139e-06

7.4614e-07

1.488226e-06

1.488226e-06

1.488226e-06

5.44548e-06

5.44548e-06

5.44548e-06

0.000238196925

0.000238196925

1.868862e-05

4.84991e-06

1.383871e-05

1.1100842e-05

4.272322e-06

6.82852e-06

1.1785527e-05

1.1785527e-05

3.117517e-05

8.15968e-07

4.33452e-07

3.348506e-06

7.325725e-06

4.222911e-06

1.16048e-06

1.347791e-05

3.90218e-07

0.000165446766

2.2674162e-05

4.45621e-07

0.000114681142

1.488224e-06

1.7433696e-05

1.22395e-06

7.499971e-06

0.00110401133

0.00110401133

1.535602e-06

1.535602e-06

0.000181500517

0.000181500517

7.51175e-06

7.51175e-06

6.02106579999999e-05

3.8570081e-05

5.758511e-06

1.5882066e-05

1.8594831e-05

1.3003969e-05

3.83901e-07

5.206961e-06

0.000834212350999996

4.55986e-07

0.000206677377

0.000627078987999999

4.45621e-07

4.45621e-07

1.882465e-05

1.882465e-05

1.882465e-05

1.882465e-05

0.0998840088650088

8.264804e-06

8.264804e-06

1.931592e-06

1.931592e-06

6.333212e-06

6.333212e-06

0.000387879077999999

0.000387879077999999

2.198917e-05

1.544381e-06

2.0444789e-05

3.95826e-07

3.95826e-07

0.000365038095999999

3.743221e-05

0.000327605885999999

4.55986e-07

4.55986e-07

1.48822e-06

1.48822e-06

1.48822e-06

1.48822e-06

3.72056e-07

3.72056e-07

3.72056e-07

3.72056e-07

1.926144e-06

1.926144e-06

1.926144e-06

1.152492e-06

7.73652e-07

0.00156876686499998

0.001026821107

7.67801e-07

7.67801e-07

0.001026053306

1.17549e-05

0.00101391450500001

3.83901e-07

7.33736930000001e-05

7.33736930000001e-05

8.82972e-06

6.3776172e-05

7.67801e-07

0.000468572064999998

0.000324744686999999

3.82804639999999e-05

0.000286464222999999

0.000143827378

0.000116571721

1.9169658e-05

7.67802e-07

7.318197e-06

4.5900906e-05

4.5900906e-05

4.5900906e-05

4.5900906e-05

0.0831710838789999

2.106511e-06

2.106511e-06

8.73504e-07

1.233007e-06

0.0831689773679999

0.077737765268999

3.3874061e-05

5.62315709999999e-05

0.000596638895000006

0.0289277874909988

0.0481232332510165

8.2729334e-05

8.2729334e-05

0.00488849028300021

0.00477578747300019

0.00011270281

6.693556e-06

4.304377e-06

2.389179e-06

1.5998398e-05

1.5998398e-05

0.000433745671

0.000433745671

1.151702e-06

3.83901e-07

7.67801e-07

2.403155e-06

2.403155e-06

0.00533300797000023

0.00533300797000023

4.4647e-06

1.34535e-06

3.11935e-06

0.000174202204

0.000174202204

0.000307508518000001

7.2851448e-05

4.18628e-06

0.00023047079

0.001386553191

0.001013289797

2.06261e-07

0.000372150122999997

9.0701e-07

4.12522e-07

2.06261e-07

2.06261e-07

3.09461e-06

3.09461e-06

0.00318062164799997

2.405432e-06

4.55985e-06

0.00141828094600002

0.001754463448

9.11972e-07

3.3262156e-05

1.790587e-06

3.1471569e-05

7.109846e-06

7.109846e-06

0.000235778575

0.000176957552

5.88210229999999e-05

0.000567319047000008

0.000192040836

3.719312e-06

3.719312e-06

0.000188321524

3.825432e-06

0.000184496092

0.000372590906999998

0.000360888495999998

0.000360888495999998

2.20925e-07

2.20925e-07

1.11617e-06

1.11617e-06

1.0365316e-05

8.829713e-06

1.535603e-06

2.687304e-06

2.687304e-06

2.687304e-06

0.00862644320100021

0.000807668292000009

0.000807668292000009

1.1517e-06

2.6950653e-05

0.000662686764000008

4.45621e-07

2.303401e-06

1.919504e-06

9.76342440000001e-05

1.18891e-05

7.67802e-07

1.919503e-06

0.00781877490900012

0.00685447821000009

7.67801e-07

0.000873528241000001

0.00115775520399999

3.9324918e-05

3.72056e-07

0.00477863336000001

2.56103e-06

1.5356e-06

6.142402e-06

6.142402e-06

0.000958154297000006

0.000612668907

2.06261e-07

1.513941e-06

1.92535e-06

5.20512e-06

0.000295318868999999

4.1315849e-05

0.00011404322

0.00011404322

0.00011404322

7.275996e-05

4.12832599999999e-05

1.17065e-06

1.17065e-06

1.17065e-06

1.17065e-06

1.043282e-05

1.043282e-05

1.9195e-06

1.9195e-06

4.892965e-06

4.456213e-06

4.36752e-07

1.782481e-06

1.782481e-06

1.837874e-06

1.837874e-06

4.4374402e-05

2.923911e-06

2.923911e-06

7.74119e-07

1.135344e-06

1.014448e-06

4.1450491e-05

4.1450491e-05

4.106659e-05

3.83901e-07

1.535603e-06

1.535603e-06

1.535603e-06

1.535603e-06

2.4832093e-05

2.4832093e-05

2.4832093e-05

2.4832093e-05

2.4832093e-05

0.000791829326999997

2.5517753e-05

9.11971e-07

9.11971e-07

9.11971e-07

3.72056e-07

3.72056e-07

3.72056e-07

2.4233726e-05

2.4233726e-05

2.4233726e-05

4.22887e-06

4.22887e-06

4.22887e-06

4.22887e-06

0.000669958668999998

0.000379070263000001

1.7206872e-05

7.29577e-06

9.911102e-06

3.1203468e-05

1.250805e-06

6.633326e-06

2.3319337e-05

7.67801e-07

7.67801e-07

9.040428e-06

7.30662e-06

1.733808e-06

0.000292447109000001

3.0887306e-05

0.000254174875

7.384928e-06

1.154384e-05

1.154384e-05

1.5850294e-05

1.5850294e-05

2.4265e-07

2.4265e-07

7.67801e-07

7.67801e-07

1.572154e-06

1.572154e-06

1.572154e-06

6.77800949999999e-05

2.305897e-06

2.305897e-06

9.49982e-06

9.49982e-06

4.204492e-06

1.051122e-06

3.15337e-06

4.6686099e-05

4.6686099e-05

5.083787e-06

5.083787e-06

4.5749878e-05

2.23842e-06

2.23842e-06

2.97645e-06

2.97645e-06

1.0278829e-05

1.0278829e-05

2.8390829e-05

2.8390829e-05

1.86535e-06

7.4614e-07

1.11921e-06

0.000175786279

1.488224e-06

1.116168e-06

3.72056e-07

3.563956e-06

3.72056e-07

3.1919e-06

6.003741e-06

6.003741e-06

1.18748e-06

1.18748e-06

2.810742e-06

2.810742e-06

1.547306e-06

3.86826e-07

1.16048e-06

0.000147651062

0.000147651062

1.1533768e-05

1.04176e-05

1.116168e-06

6.715947e-06

6.715947e-06

3.720556e-06

3.720556e-06

2.61149e-06

2.61149e-06

3.83901e-07

3.83901e-07

8.426238e-05

8.426238e-05

2.20925e-07

2.20925e-07

1.956374e-06

1.956374e-06

8.2085081e-05

3.7307e-07

8.1712011e-05

1.145708e-06

1.145708e-06

1.145708e-06

3.72056e-07

7.73652e-07

4.092622e-06

3.72056e-07

3.72056e-07

3.72056e-07

3.72056e-07

3.720566e-06

3.720566e-06

3.720566e-06

3.720566e-06

0.001378223312

2.2379787e-05

2.2379787e-05

4.45621e-07

4.45621e-07

1.936695e-05

1.936695e-05

2.567216e-06

2.567216e-06

5.5027699e-05

4.152235e-06

4.152235e-06

4.152235e-06

7.44112e-07

7.44112e-07

7.44112e-07

7.813174e-06

3.348504e-06

3.348504e-06

1.860282e-06

1.860282e-06

2.604388e-06

2.604388e-06

4.0457898e-05

4.0457898e-05

4.0457898e-05

1.488224e-06

1.488224e-06

1.488224e-06

3.72056e-07

3.72056e-07

3.72056e-07

0.00114793690399999

3.72056e-07

3.72056e-07

3.72056e-07

4.464668e-06

4.464668e-06

4.464668e-06

6.337116e-06

1.860276e-06

1.860276e-06

4.47684e-06

4.47684e-06

6.324948e-06

7.44112e-07

7.44112e-07

4.836724e-06

4.836724e-06

7.44112e-07

7.44112e-07

6.5776197e-05

1.1821056e-05

2.210654e-06

9.610402e-06

1.9454712e-05

7.44112e-07

1.87106e-05

3.2640149e-05

3.2640149e-05

1.86028e-06

1.86028e-06

0.000618616370999998

0.000618616370999998

0.000618616370999998

3.392162e-05

3.392162e-05

2.8625328e-05

3.721576e-06

4.58546e-07

1.11617e-06

3.2107027e-05

7.347544e-06

6.101984e-06

1.24556e-06

1.860282e-06

1.860282e-06

1.2853695e-05

1.0621359e-05

2.232336e-06

1.0045506e-05

1.0045506e-05

4.939731e-06

4.939731e-06

3.169416e-06

1.336863e-06

4.33452e-07

8.75289249999999e-05

8.75289249999999e-05

1.860276e-06

8.40517779999999e-05

1.616871e-06

1.7289174e-05

7.243658e-06

7.243658e-06

1.0045516e-05

1.0045516e-05

6.1372872e-05

2.604394e-06

2.232338e-06

3.72056e-07

3.026264e-06

3.026264e-06

5.5742214e-05

4.652614e-06

7.077114e-06

3.72056e-07

2.381031e-06

4.1259399e-05

0.000169084297

2.604394e-06

2.232338e-06

3.72056e-07

0.000103541029

0.000102231518

1.309511e-06

6.2566818e-05

6.2566818e-05

3.72056e-07

3.72056e-07

3.9801902e-05

2.4040398e-05

2.4040398e-05

1.5761504e-05

1.5761504e-05

4.2638564e-05

9.258494e-06

9.258494e-06

7.266574e-06

6.5471e-07

1.33721e-06

3.338007e-05

1.49228e-05

2.23842e-06

1.268438e-05

1.11921e-06

1.11921e-06

1.733806e-05

1.733806e-05

2.68094019999999e-05

2.5571836e-05

2.5571836e-05

2.5571836e-05

1.237566e-06

1.237566e-06

1.237566e-06

4.563009e-06

4.563009e-06

4.563009e-06

4.563009e-06

1.7317868e-05

6.827624e-06

6.827624e-06

6.827624e-06

1.0044623e-05

1.0044623e-05

1.733808e-06

8.310815e-06

4.45621e-07

4.45621e-07

4.45621e-07

6.1550079e-05

1.9446056e-05

1.9446056e-05

1.9074e-05

3.72056e-07

4.2104023e-05

3.7267295e-05

2.2570861e-05

7.44112e-07

1.86535e-06

1.2086972e-05

4.836728e-06

3.72056e-07

4.464672e-06

9.865284e-06

9.865284e-06

9.865284e-06

7.13376e-06

7.13376e-06

7.13376e-06

2.731524e-06

2.731524e-06

2.731524e-06

1.508616e-06

1.508616e-06

1.508616e-06

1.508616e-06

1.508616e-06

1.508616e-06

0.00111114371499999

3.2433174e-05

3.2433174e-05

3.2433174e-05

3.2433174e-05

3.2433174e-05

0.000636555054999998

8.95735e-07

8.95735e-07

8.95735e-07

8.95735e-07

0.000635659319999998

0.000635659319999998

3.72056e-07

3.72056e-07

2.0416728e-05

7.294176e-06

1.3122552e-05

2.155554e-06

2.155554e-06

3.037844e-06

2.665788e-06

3.72056e-07

0.000253971947

0.000253971947

4.05924e-06

4.07984e-07

2.342116e-06

1.30914e-06

1.7775341e-05

4.3638e-07

1.4864791e-05

2.47417e-06

1.3244871e-05

7.773045e-06

5.471826e-06

0.000299327408

1.8346708e-05

0.000230507282

1.8823737e-05

3.72056e-07

1.13433e-06

1.5356e-06

2.8607695e-05

1.8617542e-05

1.8617542e-05

2.680789e-06

2.23234e-06

4.48449e-07

0.000442155485999998

2.275578e-06

2.275578e-06

2.275578e-06

1.88536e-06

3.90218e-07

0.000438763737999998

1.488224e-06

1.488224e-06

1.488224e-06

9.228854e-05

9.228854e-05

9.228854e-05

5.436807e-06

5.436807e-06

5.436807e-06

3.7607645e-05

3.7607645e-05

3.7607645e-05

0.000294873465999999

0.000294873465999999

0.000294873465999999

2.976444e-06

2.976444e-06

2.976444e-06

4.092612e-06

4.092612e-06

4.092612e-06

1.11617e-06

1.11617e-06

1.11617e-06

1.11617e-06

1.441131e-06

1.441131e-06

1.441131e-06

2.20925e-07

2.20925e-07

2.20925e-07

1.220206e-06

1.220206e-06

1.220206e-06

0.83928396243887

0.370048359720182

0.00142899748099994

0.00142899748099994

0.00142899748099994

0.00142899748099994

4.277013e-06

6.32868e-07

6.32868e-07

3.90218e-07

2.4265e-07

3.644145e-06

8.20653e-07

8.20653e-07

3.7307e-07

3.7307e-07

9.43355e-07

3.72056e-07

5.71299e-07

1.507067e-06

1.507067e-06

0.368615085226189

4.6660641e-05

1.154627e-06

1.154627e-06

2.059477e-06

4.55986e-07

7.2149e-07

8.82001e-07

2.889e-06

2.889e-06

5.176702e-06

2.675702e-06

2.501e-06

2.231345e-05

2.231345e-05

6.888088e-06

6.888088e-06

3.72056e-07

3.72056e-07

5.435185e-06

5.435185e-06

3.72056e-07

3.72056e-07

4.448317e-06

4.448317e-06

1.575196e-06

2.873121e-06

7.875423e-06

3.90218e-07

3.90218e-07

3.847688e-06

1.896603e-06

1.951085e-06

3.637517e-06

2.73591e-06

9.01607e-07

2.60439e-06

2.60439e-06

2.60439e-06

0.0216594395110004

1.7459812e-05

1.7459812e-05

0.0190976832090004

0.00152545878

4.641694e-06

4.55986e-07

0.00295652694999997

6.858899e-06

0.0146037409000003

8.35674e-07

8.35674e-07

2.07316e-06

2.07316e-06

2.436066e-06

2.436066e-06

1.33686e-06

1.33686e-06

0.000113833435

0.000113833435

0.00229897598999998

0.001915842234

8.82001e-07

0.000382251755

0.000124805305

5.85327e-06

4.5980586e-05

7.2971449e-05

7.44112e-07

7.44112e-07

7.44112e-07

1.754786e-06

1.754786e-06

1.754786e-06

0.000139980294

1.4047854e-05

1.28772e-05

1.170654e-06

7.44112e-07

7.44112e-07

2.276646e-06

2.276646e-06

7.771242e-06

9.1871e-07

6.852532e-06

9.12835289999999e-05

9.12835289999999e-05

3.83901e-07

3.83901e-07

3.72056e-07

3.72056e-07

1.6458001e-05

1.6458001e-05

5.875152e-06

5.875152e-06

7.67801e-07

7.67801e-07

1.5072301e-05

1.5072301e-05

1.95109e-06

2.554688e-06

1.0566523e-05

0.0278410740419974

0.0278410740419974

0.0278410740419974

0.0720281764930102

7.85622059999999e-05

7.81719879999999e-05

3.90218e-07

8.26602e-06

5.77192e-06

2.4941e-06

0.0309404513379987

1.815036e-06

7.699384e-06

0.00180628538699995

8.17677e-07

1.523758e-06

1.56087e-06

0.0018340553

7.44112e-07

0.00589726839900023

2.71336e-06

1.257122e-06

0.0213847109329991

8.15968e-07

8.15968e-07

2.427715e-05

3.263541e-06

2.1013609e-05

2.2242429e-05

7.80435e-07

2.1461994e-05

0.0179690095740013

0.00164497221400002

0.0162743730960003

4.9664264e-05

0.0111100735529998

0.000106914049

0.0109286161749998

7.45433289999999e-05

8.196574e-06

2.724742e-06

5.471832e-06

0.00803343179400053

0.0031985984499999

0.00482809872100024

2.025572e-06

1.33686e-06

3.372191e-06

0.00383284988699987

0.00383284988699987

0.246867254916065

3.13915e-06

3.13915e-06

1.187478e-06

3.95826e-07

7.91652e-07

4.45621e-07

4.45621e-07

2.18385e-06

2.18385e-06

0.0311084941899999

3.793859e-06

0.0213553966509999

0.00974930368000004

0.002578163785

0.002576531851

1.631934e-06

0.00398830610300001

0.00013660018

4.55986e-07

0.00385124993700003

0.00189331870999999

6.18783e-07

0.000229885339

0.00166281458799999

0.00124098902900001

1.5657003e-05

2.6673768e-05

0.000109182642

0.000543843132999999

0.000545632482999997

0.001281416747

0.000867824658999996

4.45621e-07

0.000150141464

0.000263005003

0.204769610253079

0.204769610253079

0.412165797497183

0.0749637797790123

2.5563741e-05

1.023085e-06

2.4265e-07

7.80435e-07

3.83901e-07

3.83901e-07

2.4156755e-05

2.2500802e-05

1.655953e-06

0.000843819994999997

3.83901e-07

3.83901e-07

0.00013317998

1.66033e-06

0.00013151965

2.475145e-06

2.475145e-06

8.0848686e-05

3.86826e-07

3.814251e-06

2.0701009e-05

5.59466e-05

3.1754329e-05

3.1754329e-05

0.000345838546

4.97032e-06

0.00021342042

4.078164e-06

0.000123369642

7.719284e-05

1.4454334e-05

1.0592661e-05

5.2145845e-05

0.000136309214

4.1771317e-05

1.5427675e-05

7.9110222e-05

2.7291632e-05

2.7291632e-05

8.545722e-06

3.38403e-06

5.161692e-06

0.000863495342999991

4.9472007e-05

8.20506e-07

1.154124e-06

4.7497377e-05

0.000814023335999991

0.0005506287

4.48449e-07

3.7669e-05

0.000225277187000001

0.0635919071250077

0.00355342441

1.644406e-06

7.816466e-06

0.002944485227

0.000208913982

0.000390564329

0.00298804519199999

0.00298804519199999

0.0570504375230061

0.0570420195950061

1.357961e-06

4.3638e-07

6.623587e-06

0.000542956825

0.000433799670000002

3.90218e-07

5.315477e-06

4.9708125e-05

9.70599e-07

3.90218e-07

3.90218e-07

7.80435e-07

7.80435e-07

6.32868e-07

0.000365252277000002

9.1888e-06

0.000107001079

4.6414735e-05

6.0586344e-05

2.156076e-06

2.156076e-06

0.00762094607500041

0.00762094607500041

0.00762094607500041

0.001475090675

8.1577746e-05

8.1577746e-05

2.5369078e-05

2.5369078e-05

0.000208162299

0.000208162299

0.000888127906000003

0.000184794562999999

1.551276e-06

0.000701782067

7.44112e-07

7.44112e-07

0.000271109534

0.000201228956

6.9112777e-05

7.67801e-07

0.336298430200095

0.00152023574300001

0.00017033108

3.83901e-07

3.0948167e-05

1.968481e-05

0.000119314202

1.345348e-06

1.345348e-06

5.83066379999999e-05

5.83066379999999e-05

0.000161675789

1.209651e-06

0.000160466138

0.000470937524

1.70701e-05

0.000453867424

9.92954700000001e-05

7.67801e-07

9.949244e-06

8.85784250000001e-05

0.000558343894000001

0.000351844955

0.000206498939

0.282099366933032

0.282099366933032

0.282099366933032

1.3743237e-05

2.113151e-06

2.113151e-06

2.27993e-06

2.27993e-06

8.96333e-06

8.96333e-06

3.86826e-07

3.86826e-07

0.0077533977469994

3.324948e-06

2.16447e-06

1.160478e-06

7.67801e-07

7.67801e-07

2.482653e-05

9.158489e-06

4.48449e-07

1.5219592e-05

0.00173940432799998

0.00173940432799998

0.000295310854

0.000295310854

0.00552650994299988

2.520664e-06

8.77754379999999e-05

0.00543621384099989

6.323488e-05

1.44298e-06

7.370449e-06

1.86535e-06

1.76046e-05

3.4951501e-05

9.8673115e-05

2.9222571e-05

6.9450544e-05

1.345348e-06

1.345348e-06

0.0449065608770153

0.000146867693

0.000146867693

2.98456e-06

2.98456e-06

2.4447442e-05

2.295719e-05

1.490252e-06

0.00635629668600016

1.18032e-06

0.00160528513299998

0.00474983123300003

0.0316266367970027

0.00262201896899998

0.0288039602599997

1.290591e-06

0.000138349539

6.1017438e-05

0.00659246923699993

0.00659246923699993

0.000156399916

3.947856e-06

0.00015245206

4.58546e-07

4.58546e-07

1.26431e-06

1.26431e-06

1.26431e-06

3.861353e-06

3.861353e-06

1.170653e-06

2.6907e-06

3.72056e-07

3.72056e-07

3.72056e-07

3.72056e-07

0.000903215461999984

0.000903215461999984

0.000903215461999984

0.000903215461999984

5.77192e-06

5.77192e-06

5.77192e-06

5.05043e-06

5.05043e-06

7.2149e-07

7.2149e-07

0.000805257504999994

0.000805257504999994

0.000805257504999994

0.000805257504999994

0.000805257504999994

0.0562587757980145

0.0562587757980145

0.0562587757980145

7.8301526e-05

2.72143e-05

5.1087226e-05

2.60439e-06

2.60439e-06

0.0553498475790131

0.000100143805

0.0534569612390127

3.317554e-06

2.207566e-06

0.001433762646

0.000353454769

3.720767e-05

1.97913e-06

3.522854e-05

8.68347e-07

8.68347e-07

0.000789946285999995

0.000789946285999995

0.00116550957899999

7.443152e-06

7.443152e-06

7.443152e-06

7.443152e-06

7.443152e-06

2.232338e-06

2.232338e-06

2.232338e-06

2.232338e-06

2.232338e-06

4.45770060000001e-05

4.45770060000001e-05

3.6709349e-05

3.6709349e-05

3.6709349e-05

7.867657e-06

7.867657e-06

7.867657e-06

6.0292686e-05

2.600714e-06

2.600714e-06

2.600714e-06

2.600714e-06

5.471831e-06

5.471831e-06

5.471831e-06

5.471831e-06

2.3632103e-05

2.3632103e-05

2.3632103e-05

2.3632103e-05

1.9596144e-05

1.9596144e-05

1.9596144e-05

1.9596144e-05

8.991894e-06

8.991894e-06

8.991894e-06

8.991894e-06

2.8092159e-05

1.936669e-06

1.936669e-06

1.936669e-06

1.936669e-06

2.5783434e-05

2.5783434e-05

2.5783434e-05

2.5783434e-05

3.72056e-07

3.72056e-07

3.72056e-07

3.72056e-07

6.5467209e-05

2.977464e-06

2.977464e-06

2.977464e-06

2.977464e-06

5.5346433e-05

5.5346433e-05

5.5346433e-05

5.5346433e-05

7.143312e-06

7.143312e-06

7.143312e-06

7.143312e-06

4.096608e-06

4.096608e-06

4.096608e-06

4.096608e-06

4.096608e-06

1.0836822e-05

7.706596e-06

7.44112e-07

7.44112e-07

7.44112e-07

2.06261e-07

2.06261e-07

2.06261e-07

2.988293e-06

2.988293e-06

2.988293e-06

3.76793e-06

3.76793e-06

3.76793e-06

3.130226e-06

3.130226e-06

3.130226e-06

3.130226e-06

0.000393608057999999

6.3822681e-05

3.7307e-07

3.7307e-07

3.7307e-07

2.7511328e-05

2.7511328e-05

1.697098e-05

1.0106896e-05

4.33452e-07

3.5938283e-05

3.5938283e-05

3.5938283e-05

5.2320282e-05

5.2320282e-05

5.2320282e-05

4.9169234e-05

3.151048e-06

0.000269256102999999

2.308731e-06

2.308731e-06

1.860282e-06

4.48449e-07

7.5205943e-05

7.5205943e-05

7.5205943e-05

0.000191741428999999

7.23149659999999e-05

7.23149659999999e-05

3.429967e-06

3.429967e-06

0.000115252384

0.000115252384

7.44112e-07

7.44112e-07

8.208992e-06

8.208992e-06

8.208992e-06

8.208992e-06

5.6156511e-05

5.6156511e-05

5.6156511e-05

5.4332568e-05

9.144798e-06

4.518777e-05

1.823943e-06

1.823943e-06

3.95826e-07

3.95826e-07

3.95826e-07

3.95826e-07

3.95826e-07

1.3746166e-05

1.3746166e-05

1.3746166e-05

1.3746166e-05

1.3746166e-05

2.144988e-05

6.148953e-06

6.148953e-06

6.148953e-06

6.148953e-06

1.5300927e-05

1.5300927e-05

1.5300927e-05

1.5300927e-05

0.000457115157999999

4.2047106e-05

4.2047106e-05

4.2047106e-05

4.2047106e-05

0.000405299911999999

6.805606e-06

6.805606e-06

6.805606e-06

0.000133404642

0.000133404642

0.000133404642

3.7307e-07

3.7307e-07

3.7307e-07

8.86593259999999e-05

8.86593259999999e-05

8.86593259999999e-05

6.766306e-06

6.766306e-06

6.766306e-06

0.000169290962

0.000169290962

0.000169290962

9.76814e-06

9.76814e-06

9.76814e-06

9.76814e-06

8.08436e-07

8.08436e-07

8.08436e-07

8.08436e-07

8.08436e-07

8.08436e-07

1.177564e-06

7.44112e-07

7.44112e-07

7.44112e-07

7.44112e-07

7.44112e-07

4.33452e-07

4.33452e-07

4.33452e-07

4.33452e-07

4.33452e-07

6.229189e-06

6.229189e-06

6.229189e-06

6.229189e-06

6.229189e-06

6.229189e-06

2.4333229e-05

3.72056e-07

3.72056e-07

3.72056e-07

3.72056e-07

3.72056e-07

3.720556e-06

3.720556e-06

3.720556e-06

3.720556e-06

3.720556e-06

2.232338e-06

2.232338e-06

2.232338e-06

2.232338e-06

2.232338e-06

1.8008279e-05

1.8008279e-05

1.8008279e-05

1.8008279e-05

1.8008279e-05

0.000109355483

0.000109355483

7.44112e-07

7.44112e-07

7.44112e-07

7.44112e-07

0.000108611371

0.000102582078

0.000102582078

2.6023402e-05

3.146211e-06

4.654077e-05

2.3493657e-05

3.378038e-06

6.029293e-06

2.976448e-06

2.976448e-06

3.052845e-06

2.23234e-06

3.72056e-07

4.48449e-07

1.1517e-06

1.1517e-06

1.1517e-06

1.1517e-06

1.1517e-06

1.1517e-06

2.246582e-06

2.246582e-06

2.246582e-06

2.246582e-06

1.896208e-06

1.896208e-06

3.50374e-07

3.50374e-07

0.001213652187

3.7044207e-05

3.7044207e-05

3.7044207e-05

3.7044207e-05

3.7044207e-05

1.960807e-05

3.90218e-07

3.90218e-07

3.90218e-07

3.90218e-07

1.9217852e-05

1.9217852e-05

1.9217852e-05

1.116168e-06

1.583342e-05

2.268264e-06

3.72056e-07

3.72056e-07

3.72056e-07

3.72056e-07

3.72056e-07

0.001123782832

0.001123782832

1.2277868e-05

1.2277868e-05

1.2277868e-05

0.000593285421999998

2.604392e-06

2.604392e-06

2.232336e-06

2.232336e-06

0.000585844301999998

0.000585844301999998

2.604392e-06

2.604392e-06

7.80435e-07

7.80435e-07

7.80435e-07

8.90622089999999e-05

8.90622089999999e-05

8.90622089999999e-05

0.000428376897999999

0.000428376897999999

3.1916851e-05

1.4830052e-05

1.2233561e-05

0.000224449887

0.000144946547

1.8974544e-05

1.8974544e-05

1.8974544e-05

4.33452e-07

4.33452e-07

2.010732e-06

7.73652e-07

1.23708e-06

2.658874e-06

2.658874e-06

4.110774e-06

3.90218e-07

3.72056e-07

3.3485e-06

9.760712e-06

1.28403e-06

4.08468e-06

4.392002e-06

1.3870478e-05

2.232336e-06

2.232336e-06

2.232336e-06

2.232336e-06

1.1266086e-05

1.1266086e-05

1.1266086e-05

1.1266086e-05

3.72056e-07

3.72056e-07

3.72056e-07

3.72056e-07

2.9146382e-05

2.9146382e-05

2.9146382e-05

2.9146382e-05

2.9146382e-05

2.9146382e-05

2.9146382e-05
