# Supplementary material for: Succession of bacterial communities on carrion is independent of vertebrate scavengers
Source: PeerJ. 2020 Jun 10;8:e9307. doi: 10.7717/peerj.9307 (PMC7293191; doi:10.7717/peerj.9307)
Supplement: Table S1 — (r2 = 9.402e − 06, p = 0.985). [file peerj-08-9307-s002.docx]

| **Sample ID** | **Group** | **Shannon** | **Sequence counts** |
| --- | --- | --- | --- |
| 0COWd003 | Fresh | 6.18061 | 165305 |
| 0COWd005 | Fresh | 5.353186 | 159361 |
| 0COWd007 | Fresh | 7.218307 | 202077 |
| 0COWd042 | Fresh | 5.971943 | 38737 |
| 0COWd001 | Fresh | 5.12973 | 21968 |
| 0COWd009 | Fresh | 4.834249 | 110733 |
| 0COWd008 | Fresh | 4.908404 | 248873 |
| 0COWd004 | Bloat | 3.939196 | 106475 |
| 0COWd043 | Bloat | 4.038446 | 89452 |
| 0COWd010 | Bloat | 3.301819 | 145313 |
| 0COWd011 | Bloat | 4.399033 | 125426 |
| 0COWd017 | Bloat | 3.518111 | 87972 |
| 0COWd040 | Active Decay | 6.691777 | 127989 |
| 0COWd034 | Active Decay | 5.806441 | 104431 |
| 0COWd039 | Active Decay | 4.815487 | 109123 |
| 0COWd006 | Active Decay | 4.071687 | 135909 |
| 0COWd002 | Active Decay | 4.071197 | 196246 |
| 0COWd030 | Active Decay | 4.563853 | 109030 |
| 0COWd035 | Active Decay | 6.122499 | 124040 |
| 0COWd024 | Active Decay | 5.207748 | 230868 |
| 0COWd038 | Active Decay | 4.68487 | 127641 |
| 0COWd036 | Active Decay | 4.432199 | 103665 |
| 0COWd037 | Active Decay | 4.64366 | 103848 |
| 0COWd027 | Active Decay | 5.719252 | 122032 |
| 0COWd041 | Active Decay | 4.905314 | 123102 |
| 0COWd028 | Active Decay | 5.74467 | 109860 |
| 0COWd025 | Active Decay | 4.553374 | 120303 |
| 0COWd029 | Active Decay | 5.308686 | 116718 |
| 0COWd033 | Active Decay | 4.221587 | 115479 |
| 0COWd031 | Active Decay | 5.476024 | 66001 |
| 0COWd023 | Active Decay | 3.854249 | 106860 |
| 0COWd026 | Active Decay | 5.430422 | 106186 |
| 0COWd032 | Active Decay | 5.253893 | 215544 |
| 0COWd018 | Soil | 7.424502 | 112860 |
| 0COWd012 | Soil | 7.520243 | 119761 |
| 0COWd021 | Soil | 8.348517 | 116795 |
| 0COWd019 | Soil | 8.455025 | 134753 |
| 0COWd022 | Soil | 7.982817 | 117166 |
| 0COWd020 | Soil | 8.578369 | 102439 |
| 0COWd013 | Soil | 8.649757 | 116626 |

*Table S1: Summary table of Shannon alpha diversity index and sequence counts. There was no significant correlation between Shannon diversity and sequence counts (r^2^ =9.402e-06, p =0.985).*
