# Supplementary figures and images for: Succession of bacterial communities on carrion is independent of vertebrate scavengers
Source: PeerJ. 2020 Jun 10;8:e9307. doi: 10.7717/peerj.9307 (PMC7293191; doi:10.7717/peerj.9307)

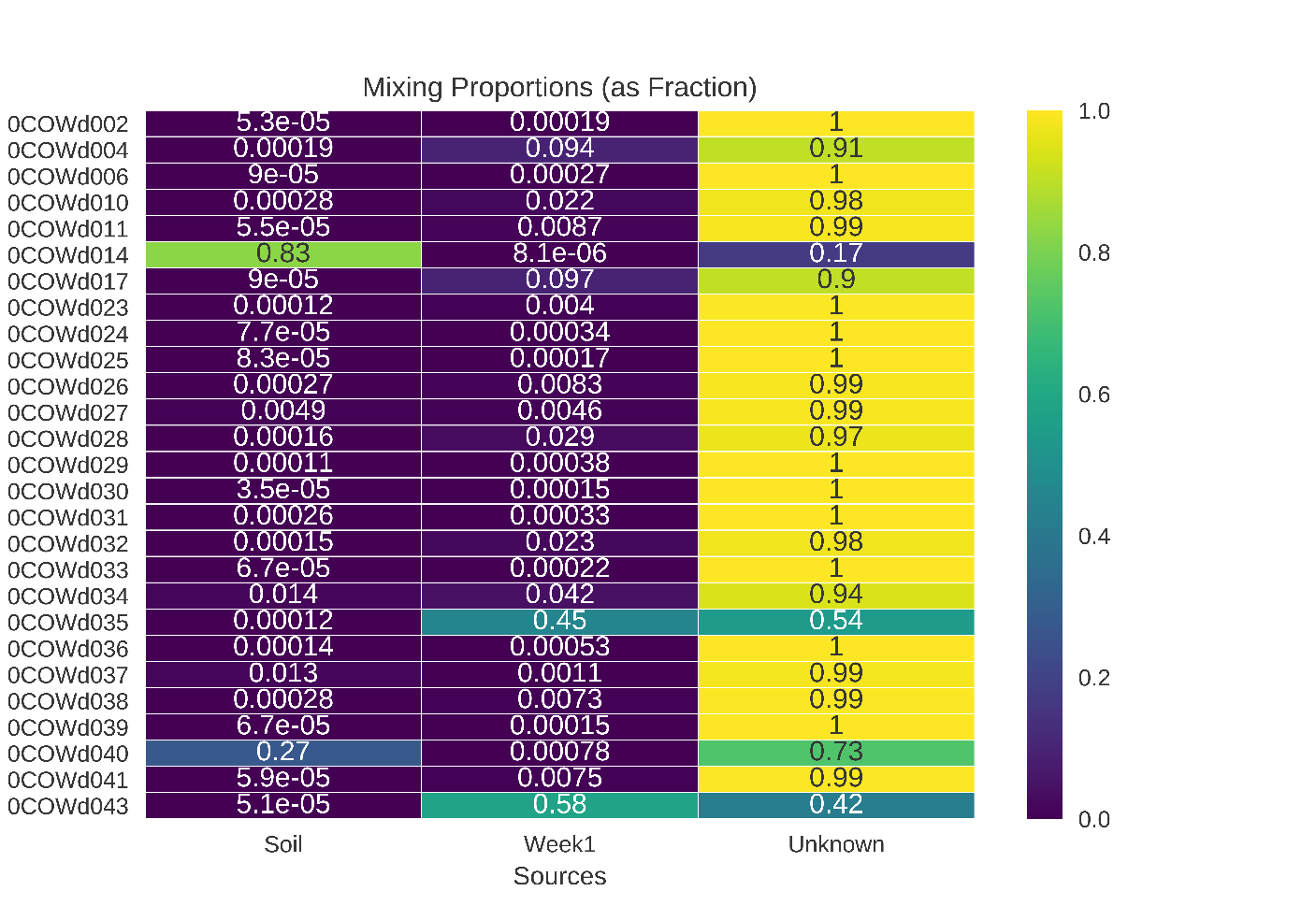

Supplement: Figure S1 — These results reflect a SourceTracker2 analysis without rarefaction. [file peerj-08-9307-s003.png]

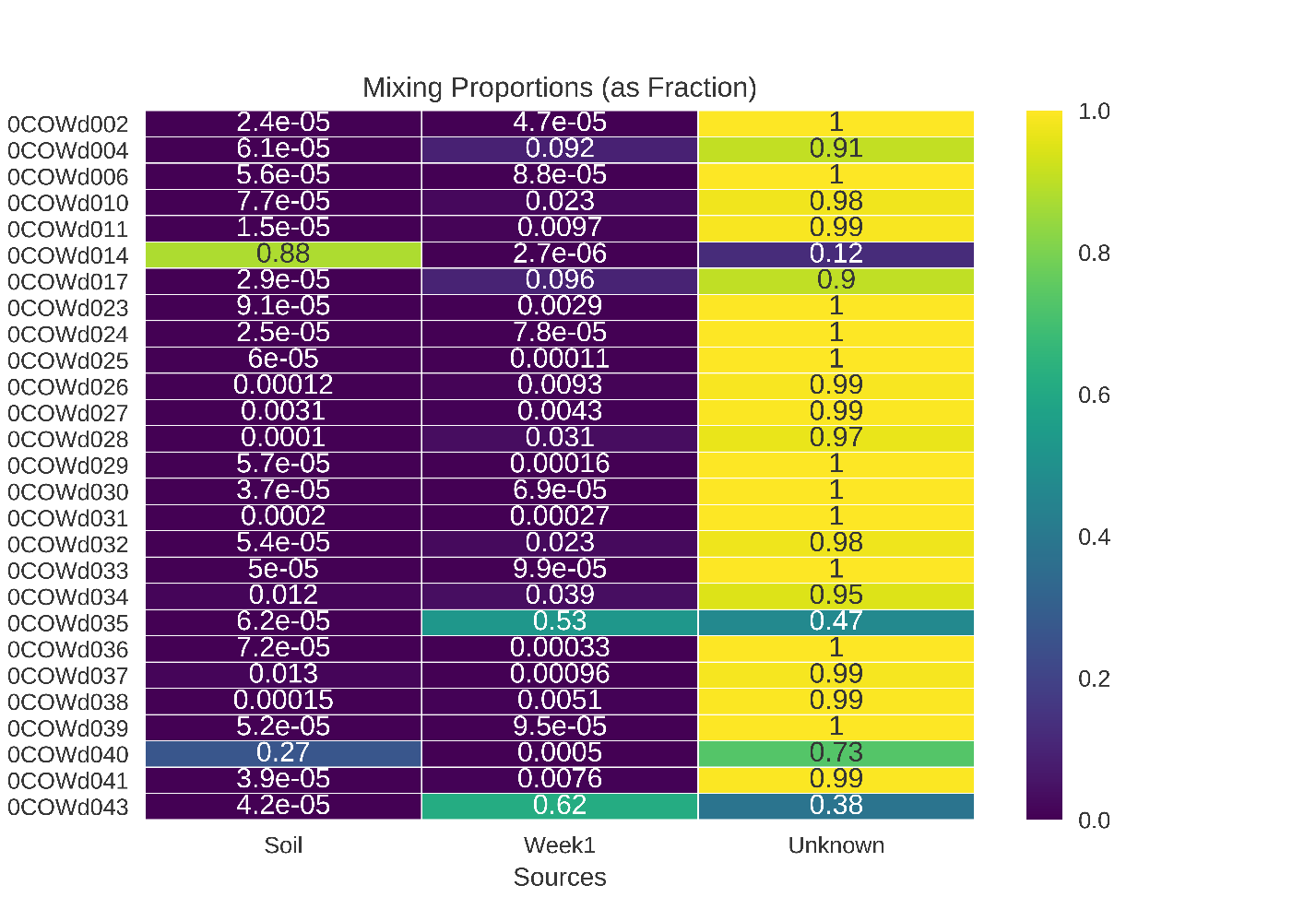

Supplement: Figure S2 — These results reflect a SourceTracker2 analysis after rarefaction down to the smallest dataset. [file peerj-08-9307-s004.png]
